# The role of IL-6-174 G/C polymorphism and intraocular IL-6 levels in the pathogenesis of ocular diseases: a systematic review and meta-analysis

**DOI:** 10.1038/s41598-020-74203-9

**Published:** 2020-10-15

**Authors:** Zulvikar Syambani Ulhaq, Gita Vita Soraya, Budu, Lely Retno Wulandari

**Affiliations:** 1 Department of Biochemistry, Faculty of Medicine and Health Sciences, Maulana Malik Ibrahim State Islamic University of Malang, Batu, East Java 65151 Indonesia; 2 Department of Biochemistry, Faculty of Medicine, Hasanuddin University, Makassar, South Sulawesi Indonesia; 3 Department of Ophthalmology, Faculty of Medicine, Hasanuddin University, Makassar, South Sulawesi Indonesia; 4 Department of Ophthalmology, Faculty of Medicine, Brawijaya University, Malang, East Java Indonesia

**Keywords:** Biomarkers, Eye diseases

## Abstract

Interleukin-6 (IL-6) is one of the key regulators behind the inflammatory and pathological process associated with ophthalmic diseases. The role of IL-6-174 G/C polymorphism as well as intraocular IL-6 levels among various eye disease patients differ across studies and has not been systematically reviewed. Thus, this study aims to provide a summary to understand the relationship between IL-6 and ophthalmic disease. In total, 8,252 and 11,014 subjects for IL-6-174 G/C and intraocular levels of IL-6, respectively, were retrieved from PubMed, Scopus and Web of Science. No association was found between IL-6-174 G/C polymorphisms with ocular diseases. Subgroup analyses revealed a suggestive association between the GC genotype of IL-6-174 G/C with proliferative diabetic retinopathy (PDR). Further, the level of intraocular IL-6 among ocular disease patients in general was found to be higher than the control group [standardized mean difference (SMD) = 1.41, 95% confidence interval (CI) 1.24–1.58, P < 0.00001]. Closer examination through subgroup analyses yielded similar results in several ocular diseases. This study thus indicates that the IL-6-174 G/C polymorphism does not predispose patients to ocular disease, although the GC genotype is likely to be a genetic biomarker for PDR. Moreover, intraocular IL-6 concentrations are related to the specific manifestations of the ophthalmic diseases. Further studies with larger sample sizes are warranted to confirm this conclusion.

## Introduction

Ocular diseases are known to adversely affect the quality of life. The most common ocular diseases such as uveitis, glaucoma, age-related macular degeneration (AMD), diabetic retinopathy (DR) and cataract can cause a spectrum of sequelae ranging from visual impairment to irreversible blindness^1^. Inflammation is a normal biological response towards pathogen invasion or host tissue injury^2^, and has been known to play a crucial role in the development and progression of ocular disease^3^. Although previous studies of the involvement of inflammation has been limited to the pathogenesis of inflammatory disorders such as uveitis and dry eye disease (DED)^3,4^, it is now widely implicated in many other ophthalmic pathologies^1^. Thus, understanding of key inflammatory mechanisms may allow the development of early detection strategies and prompt treatment for ocular disease patients.

The inflammation process is modulated by cytokines via complex cellular interactions^5^. The ocular tissue is no exception, as certain inflammatory cytokines have been implicated in eye disease pathogenesis, with IL-6 playing a prominent role in particular^6^ through its pleiotropic action as both a pro- and anti-inflammatory mediator^7,8^. IL-6 protein expression is regulated by the IL-6 gene located on chromosome 7p21^9^, on which several polymorphisms have been reported. The highly frequent IL-6-174 G/C (rs1800795) polymorphism is of particular importance, due to its functional effect on IL-6 promoter activity, which then influences basal IL-6 levels^10–14^. Several studies have also reported the association between IL-6-174 G/C polymorphism with ocular diseases^15–37^. However, the results are equivocal, and the causal relationship between IL-6 gene mutations and ocular pathologies remain unclear.

Localised expression of IL-6 has been detected in the anterior segment of human eyes, such as within the trabecular meshwork (TM) and endothelial cells of Schlemm’s canal (SC)^38^. In pathologic conditions such as wet AMD, immunohistochemistry of human choroidal fibrovascular tissues show strong positive staining in the stroma and retinal pigment epithelial (RPE) cells^39^, implicating the role of ocular IL-6 production in disease pathogenesis. Elevated IL-6 concentrations have also been found in the serum, tears and intraocular fluids of ocular diseases patients^40–43^, although results of serum IL-6 measurements are often inconsistent compared to those derived from intraocular fluid^43,44^. This implies that aqueous and vitreous humours more reliably reflect the immunological conditions of the eye compared to serum, and hence assessment of intraocular IL-6 levels may be a potential option for early detection of ocular inflammation and assessment of disease progression.

Despite said reports on the implications of both genotypic distributions and intraocular IL-6 level among various ophthalmic patients, results still vary across studies and has not been systematically reviewed to date. Therefore, a systematic review and meta-analysis was conducted from eligible studies to evaluate the association between IL-6-174 G/C polymorphism and to estimate intraocular IL-6 levels among various eye disease patients.

## Results

### Association between IL-6-174 G/C polymorphism with the ocular diseases

**Figure 1:**
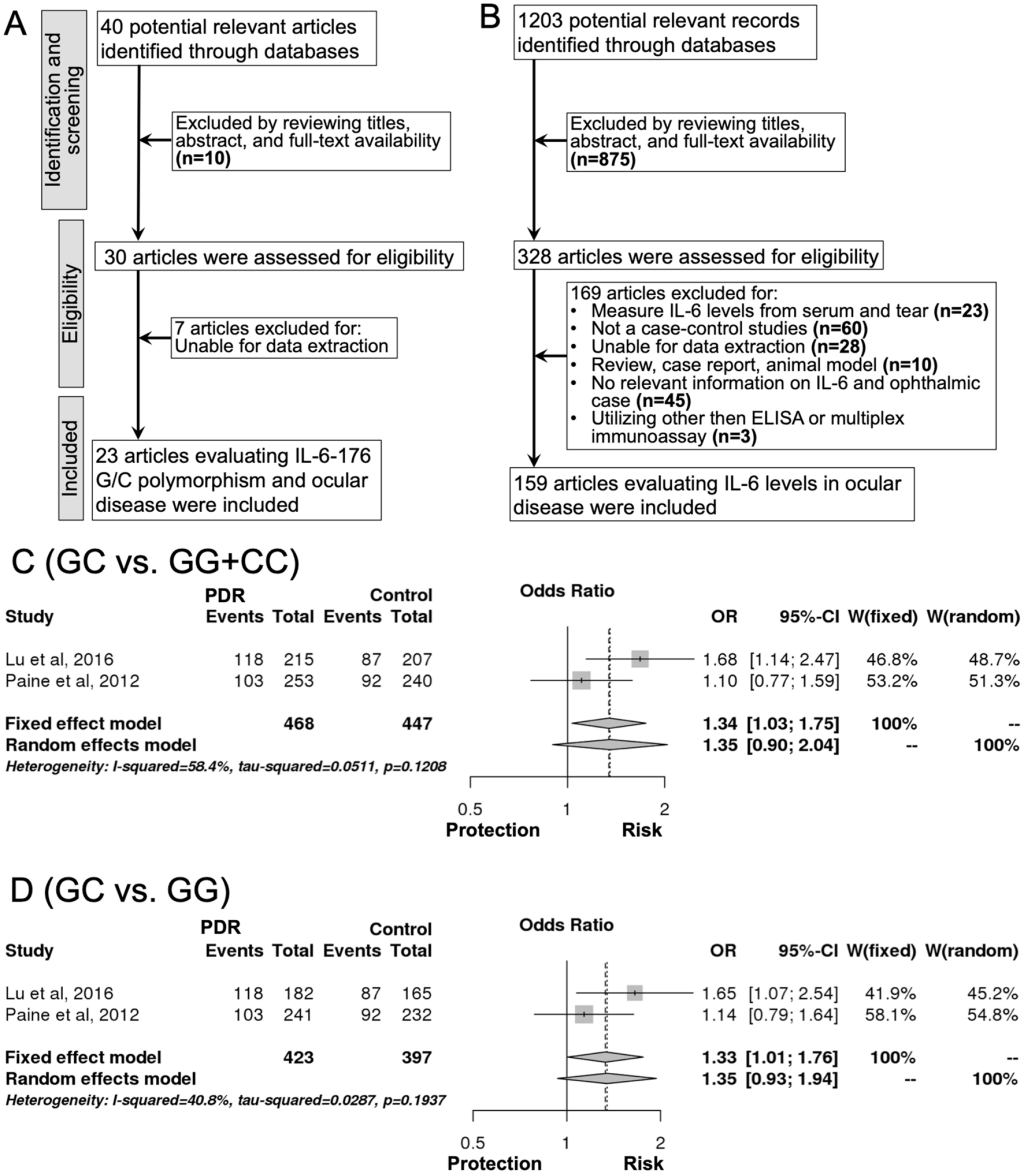
Flow diagram of the study selection process and the association of IL-6-174 G/C polymorphism with diabetic retinopathy. (**A**) Flow diagram for IL-6-174 G/C polymorphism with the ocular diseases; (**B**) Flow diagram for intraocular IL-6 levels with the ocular diseases; (**C**) Association between overdominant model in IL-6-174 G/C polymorphism with proliferative diabetic retinopathy; (**D**) Association between the heterozygous model in IL-6-174 G/C polymorphism with proliferative diabetic retinopathy.

In the literature search, a total of 40 articles were retrieved from databases. Among them, 30 were found to be relevant based on the study criteria. Seven studies were then excluded due to insufficient genotypic or allelic data. Finally, 23 studies investigating the association between IL-6-174 G/C polymorphism and ocular disease were included in this meta-analysis^15–37^ (Fig. 1A).

The 23 studies yielded a total of 8,252 subjects, which were then divided into five groups for analysis, namely glaucoma, retinopathy, surface and intraocular inflammation, vascular occlusion and congenital eye disorders. Peripheral blood sampling was used for DNA extraction followed by polymerase chain reaction (PCR)-genotyping for all the studies included in this meta-analysis. All but 4 of the studies complied with the Hardy–Weinberg equilibrium (HWE, P > 0.05)^21,22,25,28^. Details of the retrieved studies are shown in Supplemental Table S1.

The pooled result of the analysis is shown in Supplemental Table S2. Overall (1a and b), there was no significant association between IL-6-174 G/C polymorphism with the risk of ocular disease in any inheritance model, either with or without the inclusion of the four studies that deviated from the HWE (Supplemental Table S2). Similarly, subgroup analyses classified by ethnicity or racial descent (10, 11) showed no significant association in all inheritance models (Supplemental Table S2). However, subgroup analyses stratified by the type of disease [glaucoma (2), retinopathy (3a and b), DR (4), PDR (5), ocular and intraocular inflammation (6a and b), Graves’ ophthalmology (7), ocular Behcet’s disease (8), and retinal vascular occlusion (9)] revealed marginally significant associations of IL-6-174 G/C polymorphism in only the overdominant (GC vs. CC + GG, odds ratio (OR) = 1.34, 95% CI 1.03–1.75, P = 0.028, I^2^ = 58.45%, Fig. 1C, (Supplemental Table S2) and heterozygous (GC vs. GG, OR = 1.33, 95% CI 1.01–1.76, P = 0.045, I^2^ = 40.79%, Fig. 1D, (Supplemental Table S2) models of PDR subtype (5). Begg’s funnel plot (Supplemental Fig. S1A–G) and Egger’s test were applied, and no publication bias was observed (P_Egger’s test_ > 0.05, Supplemental Table S2). A sensitivity analysis was conducted by eliminating one individual study each time (Supplemental Figs. S2–S5), yet associations detected in pooled analyses remained unchanged in all inheritance models, suggesting robustness of the findings.

### The association between IL-6 levels and overall risk of ocular diseases

A total of 1,203 potentially relevant studies were initially detected. After reviewing the titles, abstract and full-text availability, 875 articles were excluded. Out of the 328 studies subsequently assessed, 169 were rejected because they did not meet the inclusion criteria. Thus, 159 studies were included in this meta-analysis^41–199^ (Fig. 1B), with the characteristics of the studies summarized in Supplemental Table S3. A total of 11,014 subjects were analysed, with the overall (1) pooled results revealing higher IL-6 levels in ocular disease patients than controls (SMD = 1.41, 95% CI 1.24–1.58, P < 0.00001, Supplemental Table S4). Analysis of the 17 subgroups yielded significant associations between intraocular IL-6 levels with glaucoma (2), pseudoexfoliation (PEX) syndrome (3), ocular inflammation 4, 4a1, 4b, 4b1–5), DR (5, 5a–c), macular oedema (MO) (6, 6a, and 6b), retinal vascular occlusion (7, 7a–c), retinal detachment (RD) (11), proliferative vitreoretinopathy (PVR) (12), retinopathy of prematurity (ROP) (13), and Coats’ disease (14). The strong association was not observed in AMD (8, 8a–c), choroidal neovascularization (CNV) (9), pachychoroid spectrum diseases (10), retinitis pigmentosa (RP) (15), epiretinal membrane (ERM) (16) and others (17) (which are consist of pseudophakia, congenital cataract, pseudophakic bullous keratopathy (PBK) and high myopia cataract (HMC)) (Supplemental Table S4). Funnel plots (see Supplemental Figs. S6, S7) and statistical tests (for overall, ocular inflammation, DR, MO, vascular occlusion and AMD: P_Egger’s test_ < 0.05, see Supplemental Table S4) provided evidence for substantial publication bias, as there were several studies with a large concentration of IL-6. A trim and fill method was implemented to correct the bias and recalculate the pooled results. However, the results of the meta-analysis remained identical, suggesting that it was not affected by publication bias.

Because substantial heterogeneity was observed in the overall pooled result (Supplemental Table S4), further analysis was performed based on additional subgroups (ethnicity, type of sample and quantification method) and using meta-regression (year of publication, type of disease, type of sample, ethnicity, quantification method and sample size) to evaluate the source of heterogeneity. The results showed that high IL-6 levels remained significantly associated with the presence of ocular disease in all subgroups except the Middle Eastern ethnicity group (Supplemental Table S5). Moreover, meta-regression analysis showed that the type of disease, quantification method and ethnicity were significantly associated with heterogeneity (Supplemental Table S6). The sensitivity analysis indicated that the association between high IL-6 levels and the presence of ocular disease was not excessively influenced by any particular study (Supplemental Fig. S8).

### The association between IL-6 levels and specific ocular diseases

#### Glaucoma

**Figure 2:**
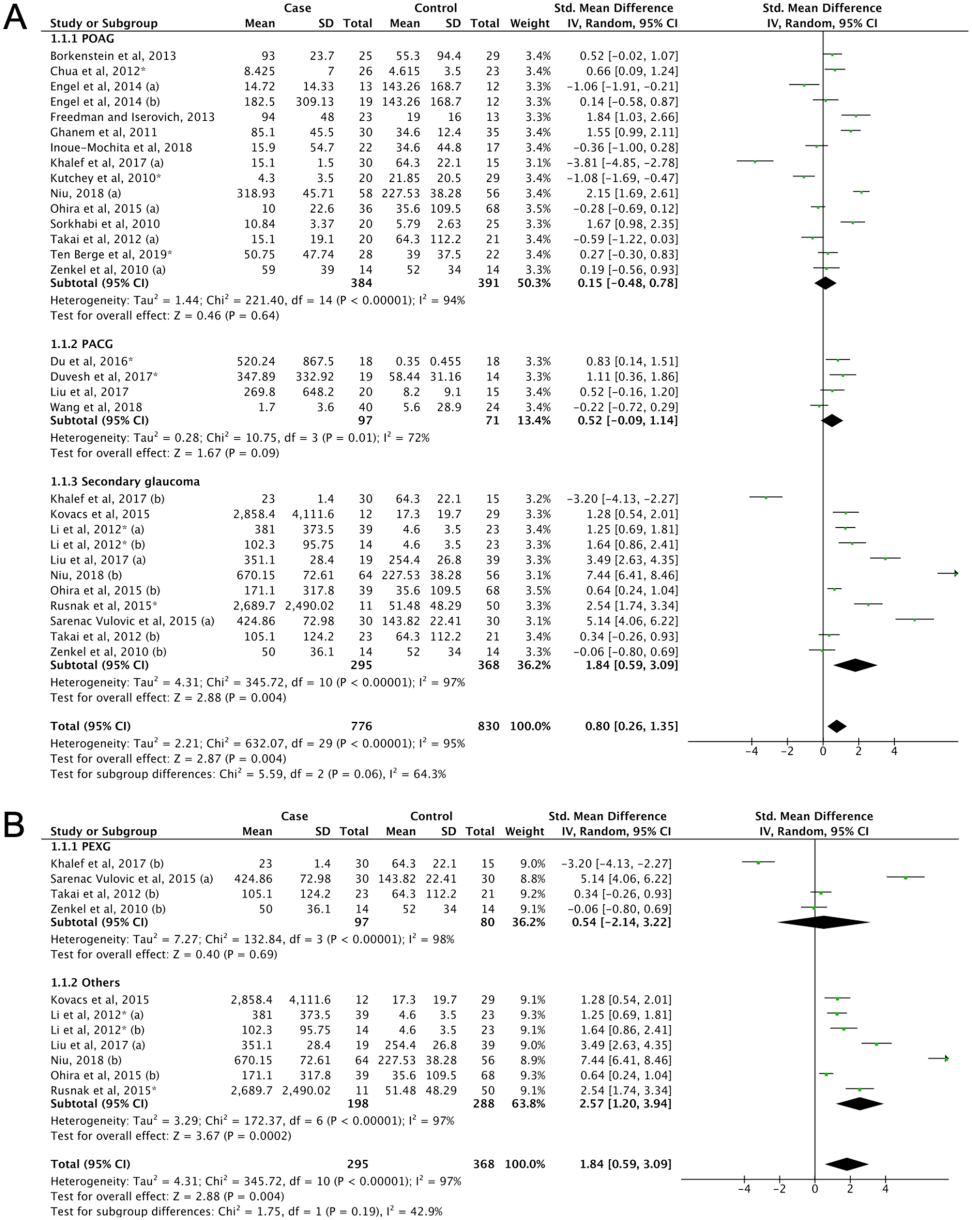
(**A**) Forest plot for pooled SMD and 95% CI for IL-6 levels between glaucoma and control; (**B**) Forest plot for pooled SMD and 95% CI for IL-6 levels between secondary glaucoma and control.

**Figure 3:**
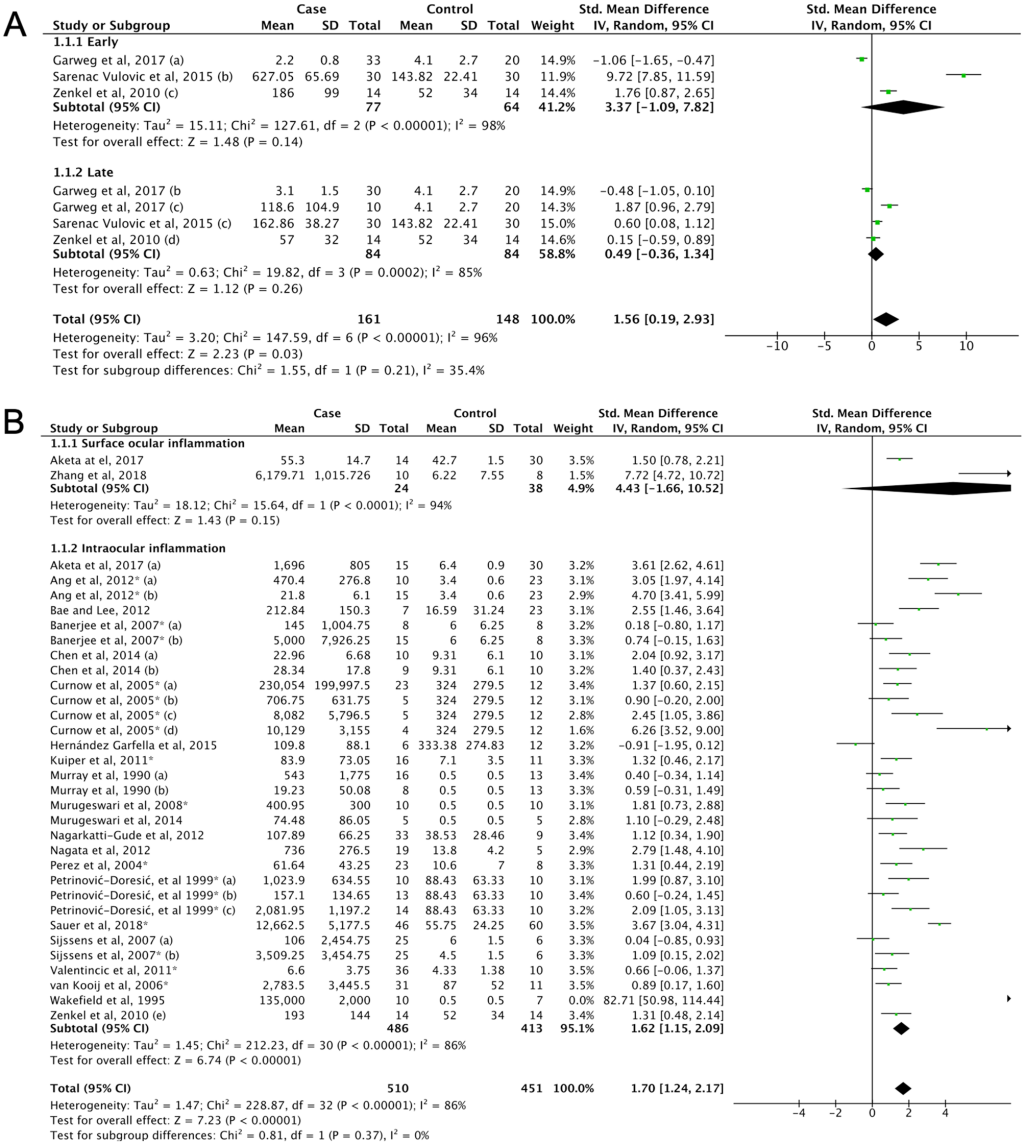
(**A**) Forest plot for pooled SMD and 95% CI for IL-6 levels between PEX syndrome and control; (**B**) Forest plot for pooled SMD and 95% CI for IL-6 levels between the ocular inflammation and control.

Glaucoma (2) was divided into three subgroups, which were primary open-angle glaucoma (POAG) (2a), primary angle-closure glaucoma (PACG) (2b), and secondary glaucoma (2c). Although the pooled result indicated that glaucoma patients tend to have higher IL-6 levels (SMD = 0.8, 95% CI 0.26–1.35, P = 0.004, Fig. 2A, Supplemental Table S4), only secondary glaucoma (which contributed 36.2% towards the total weight) showed a significant increase of IL-6 levels compared to control (SMD = 1.84, 95% CI 0.59–3.09, P = 0.004), but not to POAG or PACG (Fig. 2A, Supplemental Table S4). Further analysis revealed that other types of secondary glaucoma (a pool of neovascular glaucoma (NVG), Posner-Schlossman syndrome (PSS), uveitic glaucoma (UG) and secondary glaucoma post silicone oil tamponade) showed significantly higher IL-6 levels (SMD = 2.57, 95% CI 1.20–3.94, P = 0.004) but pseudoexfoliative glaucoma (PEXG) did not show this association (Fig. 2B). However, the PEX syndrome (3) displayed a marginally significant increase of IL-6 levels (SMD = 1.56, 95% CI 0.19–2.93, P = 0.03) (Fig. 3A, Supplemental Table S4). No significant difference was found regarding the level of IL-6 between early and late PEX syndrome (SMD = 1.69, 95% CI − 1.36–4.74, P = 0.28) (Supplemental Fig. S9). No publication biases were detected in the glaucoma and PEX syndrome groups (Supplemental Fig. S6B–C, Supplemental Table S4).

#### Ocular inflammation

The ocular inflammation group (4) was divided into the surface ocular inflammation (4a) and intraocular inflammation subgroups (4b). In calculating the estimated effect of ocular inflammation, the intraocular inflammation subgroup contributed 95.1% of the total weight. A significantly higher IL-6 level was found in the intraocular inflammation subgroup (SMD = 1.62, 95% CI 1.15–2.09, P < 0.00001), but not in the surface ocular inflammation subgroup (Fig. 3B, Supplemental Table S4). However, additional meta-analysis confirmed that IL-6 levels in tears of patient with surface ocular diseases displayed a significant increase compared to the control subjects (SMD = 2.07, 95% CI 1.67–2.48, P < 0.00001)^200–233^ (Supplemental Fig. S10). Although the funnel plot analysis showed symmetrical distribution of studies and no significant publication bias (Supplemental Fig. S6D), Egger’s test indicated publication bias in the intraocular inflammation group (see Supplemental Table S4). However, since no significant change was observed through the trim and fill method, the meta-analysis results for the subgroup of intraocular inflammation were robust. Further subtyping of intraocular inflammation generally consisted of uveitis patients classified based on the aetiology of uveitis (4b1–5). Overall, each subtype indicated the presence of upregulated IL-6 levels, except the unclassified uveitis subtype (4b1) (SMD = 0.67, 95% CI − 0.45–1.79, P = 0.24) (Supplemental Table S4, Supplemental Fig. S11). However, this result was possibly affected by a single study^76^ that showed oppositional outcome (Supplemental Fig. S11).

#### Diabetic retinopathy

**Figure 4:**
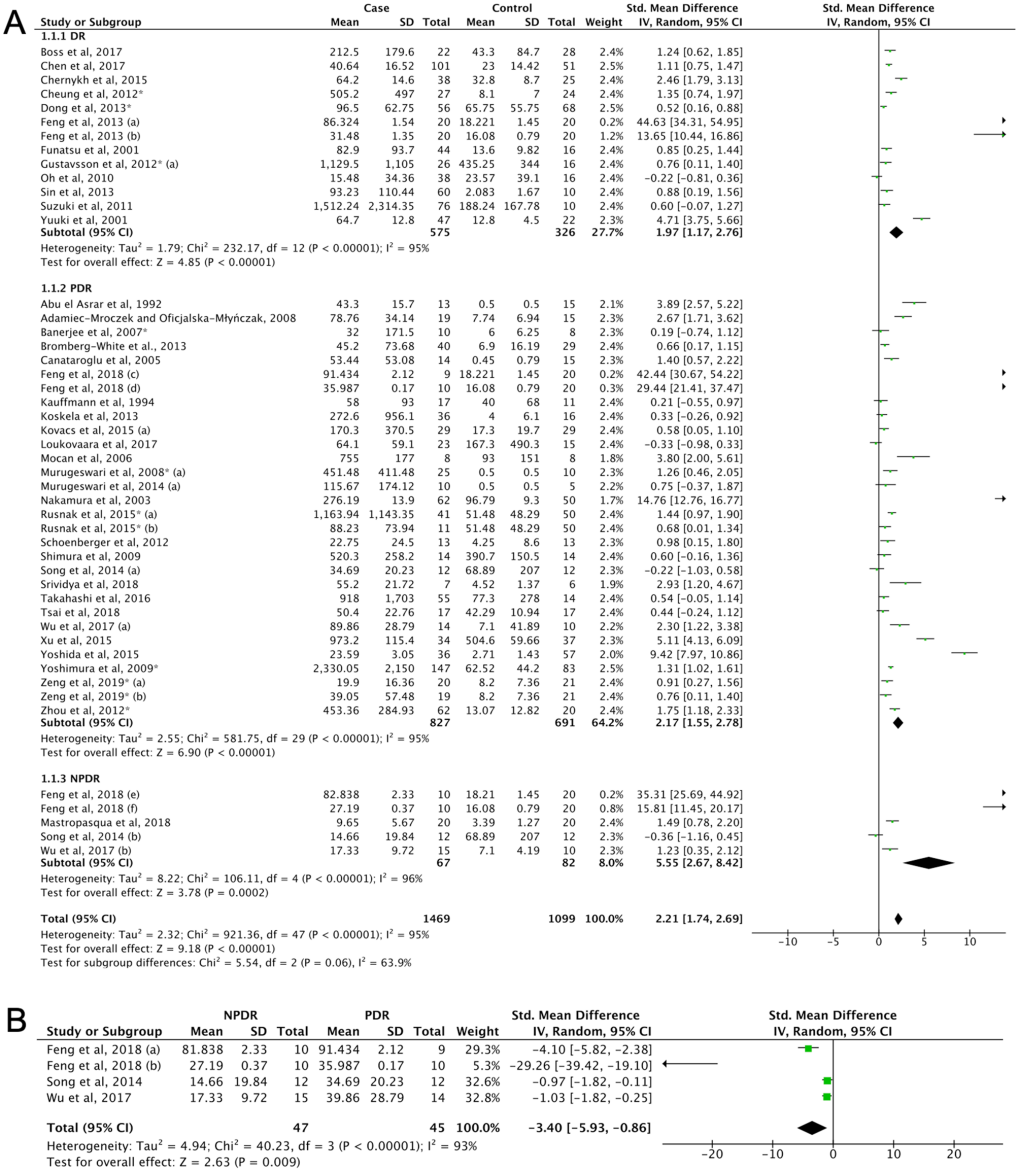
(**A**) Forest plot for pooled SMD and 95% CI for IL-6 levels between DR and control; (**B**) Forest plot for pooled SMD and 95% CI for IL-6 levels between the NPDR and PDR.

Although IL-6 levels in DR (5) has been previously examined to some extent^234^, this present meta-analysis has included additional studies^62,65,73,78,79,82,91,92,96,99,101,103,109–112,114,117–120^. The IL-6 levels were found to be higher in DR than in control group (SMD = 2.21, 95% CI 1.74–2.69, P < 0.00001). Subgroup analyses by clinical classification, yielded the same outcome (unclassified DR (5a), SMD = 1.97, 95% CI 1.17–2.76, P < 0.00001; PDR, SMD = 2.17, 95% CI 1.55–2.78, P < 0.00001) (5b); and non-proliferative diabetic retinopathy (NPDR) (5c), SMD = 5.55, 95% CI 2.67–8.42, P < 0.00001 (Fig. 4A, Supplemental Table S4). Intraocular IL-6 levels were lower in NPDR than PDR (SMD = − 3.40, 95% CI − 5.93 to − 0.86, P = 0.009, Fig. 4B). A discrepancy between the funnel plot (Supplemental Fig. S6E) and Egger’s test was detected (Supplemental Table S4), but the trim and fill method did not leverage the results and the outcome remained similar.

#### Macular oedema

**Figure 5:**
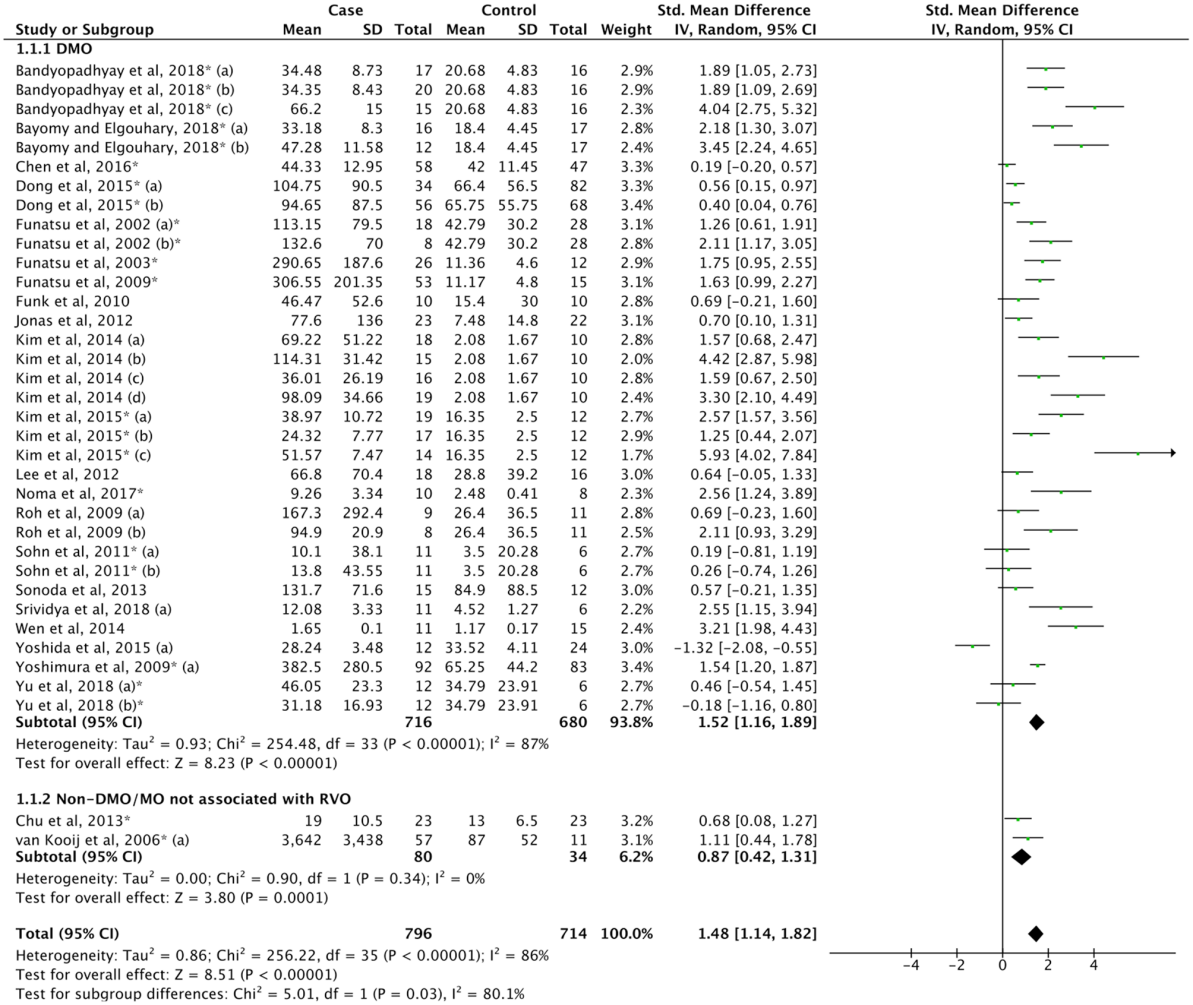
Forest plot for pooled SMD and 95% CI for IL-6 levels between MO and control.

The pooled results showed that the IL-6 levels in patients with MO (6) was higher than the control group (SMD = 1.54, 95% CI 1.20–1.87, P < 0.00001). Because substantial publication bias was reported (P_Egger’s test_ = 0.000, Supplemental Table S4), a trim and fill method was employed, but the result was still identical. Both diabetic macular oedema (DMO) (6a) and non-DMO (6b) subgroups displayed a significant increase of IL-6 levels relative to controls (SMD = 1.52, 95% CI 1.16–1.89, P < 0.00001; SMD = 0.89, 95% CI 0.42–1.31, P = 0.0001, respectively) (Fig. 5, Supplemental Table S4). Network meta-analysis to investigate the level of IL-6 in DMO patterns based on optical coherence tomography (OCT) was pooled from three studies^121,131,132^. The resuslts showed that the IL-6 levels in each group were significantly higher than control. The level of IL-6 did not significantly differ between diffuse retinal thickening (DRT), cystoid macular oedema (CMO), and serous retinal detachment (SRD) groups (Supplemental Table S7).

#### Retinal vascular occlusion

**Figure 6:**
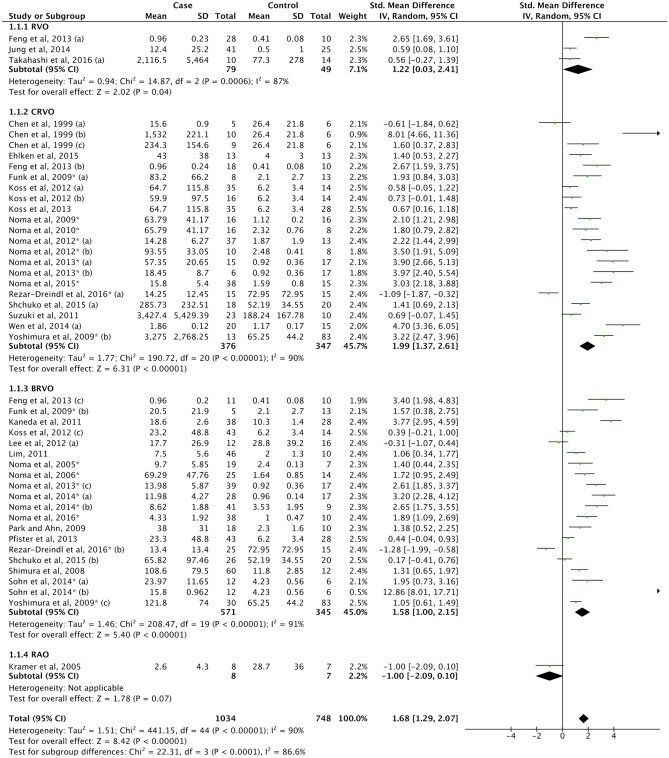
Forest plot for pooled SMD and 95% CI for IL-6 levels between retinal vascular occlusion and control.

Retinal vascular occlusions (7) consists of the retinal vein occlusion (RVO) and retinal artery occlusion (RAO) subgroups. However, since only one study reported the concentration of IL-6 in RAO^165^, results on vascular occlusion in this meta-analysis was mainly generated from the pooled RVO studies (7a) (Fig. 6, Supplemental Table S4). There was a significant increase of IL-6 levels in the vascular occlusion group in comparison to control (SMD = 1.68, 95% CI 1.29–2.07, P < 0.00001). Subgroups stratified as RVO (7a), central retinal vein occlusion (CRVO) (7b), and branch retinal vein occlusion (BRVO) (7c) showed identically significant associations with higher IL-6 levels (Fig. 6, Supplemental Table S4). However, the IL-6 levels was significantly increased in CRVO than BRVO (SMD = 0.75, 95% CI 0.25–1.25, P = 0.003) (Supplemental Fig. S12A). Subgroup analyses performed for CRVO and BRVO groups showed higher IL-6 levels in both CRVO or BRVO patients with MO than those without MO (Supplemental Fig. S12B–C). Further, the trim and fill method did not reveal publication bias present in this meta-analysis (Supplemental Fig. S7A, Supplemental Table S4), and the results remained equivalent.

#### Age-related macular degeneration

**Figure 7:**
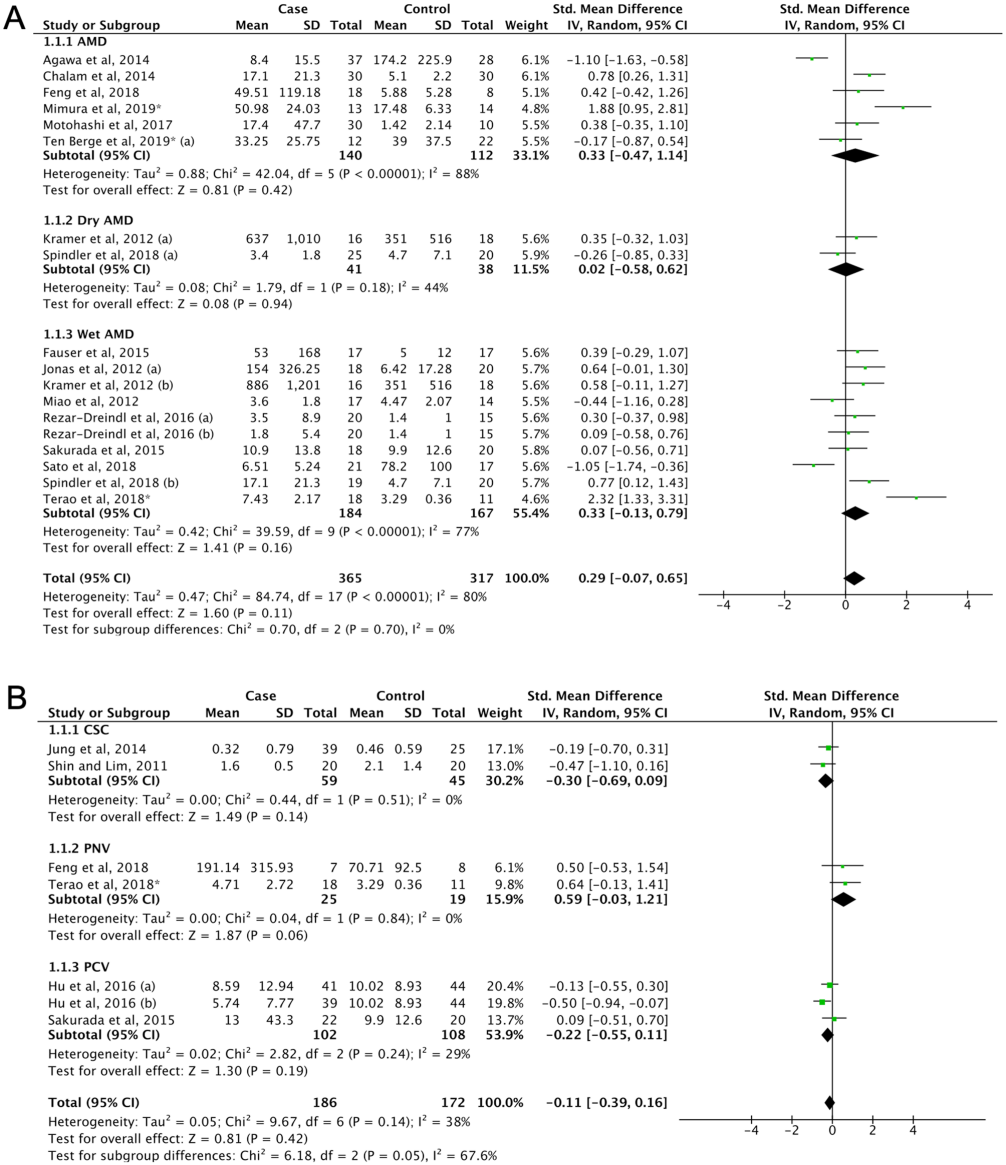
(**A**) Forest plot for pooled SMD and 95% CI for IL-6 levels between AMD and control; (**B**) Forest plot for pooled SMD and 95% CI for IL-6 levels between pachychoroid spectrum diseases and control.

The pooled result showed that IL-6 levels in AMD (8) patients did not differ with controls (SMD = 0.29, 95% CI − 0.07 to 0.65, P = 0.11, Fig. 7A). However, it should be noted that IL-6 levels tend to be higher in the wet AMD subgroup (SMD = 0.33, 95% CI − 0.13 to 0.79, P = 0.16, Fig. 7A). Thus, studies reporting CNV in AMD were then included in subgroup of wet AMD, but the result remained insignificant (SMD = 0.32, 95% CI − 0.03 to 0.66, P = 0.07, Supplemental Fig. S13A). The IL-6 levels were not different between dry and wet AMD (SMD = − 0.60, 95% CI − 1.13 to 0.12, P = 0.10, Supplemental Fig. S13B). No publication biases were reported (Supplemental Fig. S7B, Supplemental Table S4).

#### Pachychoroid spectrum diseases

Pachychoroid spectrum diseases (10) are a group of diseases characterized by choroidal thickening, dilated veins and thinning of the choriocapillaris layer^235^. In this meta-analysis, pachychoroid spectrum diseases were classified into the following three disease groups: central serous chorioretinopathy (CSC), pachychoroid neovasculopathy (PNV) and polypoidal choroidal vasculopathy (PCV). Compared to controls, no significant upregulation of IL-6 levels were found in both the pooled result nor subgroup analyses (Fig. 7B). No publication bias was identified (Supplemental Fig. S7D, Supplemental Table S4).

## Discussion

To date, this study is the first and most comprehensive meta-analysis on the role of IL-6-174 G/C polymorphism and IL-6 levels in the pathogenesis of ophthalmic diseases. The pooled analyses revealed that IL-6-174 G/C polymorphism was not associated with the ocular diseases in any inheritance models, despite increased intraocular and tear IL-6 levels. The first important notion is that the GC genotype of IL-6-174 G/C is marginally associated with PDR. Previous studies have described that the CC genotype has a trend toward a higher frequency of DR^236^, while the prevalence of metabolic syndrome is correlated with the C+ carrier individuals^237^. Despite extensive characterization of IL-6 promoter polymorphisms, conflicting results are still observed in varying populations and diseases. Several studies reported the IL-6-174C allele is associated with higher IL-6 serum concentrations^238,239^, while others state the opposite^13,14^. Despite this discrepancy, individuals with CC genotype exhibit a high body mass index (BMI) and risk of developing type 2 diabetes mellitus (T2DM)^240^. Additionally, a positive correlation between IL-6 levels and insulin resistance (IR) has also been observed in the C+ diabetic carriers^241^. Thus, this study strengthens the notion that IL-6-174C allele may be associated with the risk of developing DR.

The second important observation is that in parallel with GC genotype as a risk factor of PDR development, intraocular IL-6 level was also two-fold higher in DR patients. More importantly, the pooled result revealed that intraocular IL-6 level was higher in the PDR group compared to NPDR, which is in line with previously conducted studies^234^. The upregulation of IL-6 as an upstream transcriptional regulator of inflammation has been detected in the retina of a diabetic model^242^. It has been reported that the IL-6 knockout (KO) mice inhibit leukocyte infiltration to the retinal vessels and vascular leakage by 75% in the diabetic model^243^. The level of IL-6 has been reported linearly associated with an increase of neovascularization activity in PDR^244^. Moreover, a cross-sectional study confirmed a relationship between IR and severity of retinopathy in diabetic patients^245^. Together, these results imply that IL-6 might be critical in mediating insulin-induced vascular remodelling.

**Figure 8:**
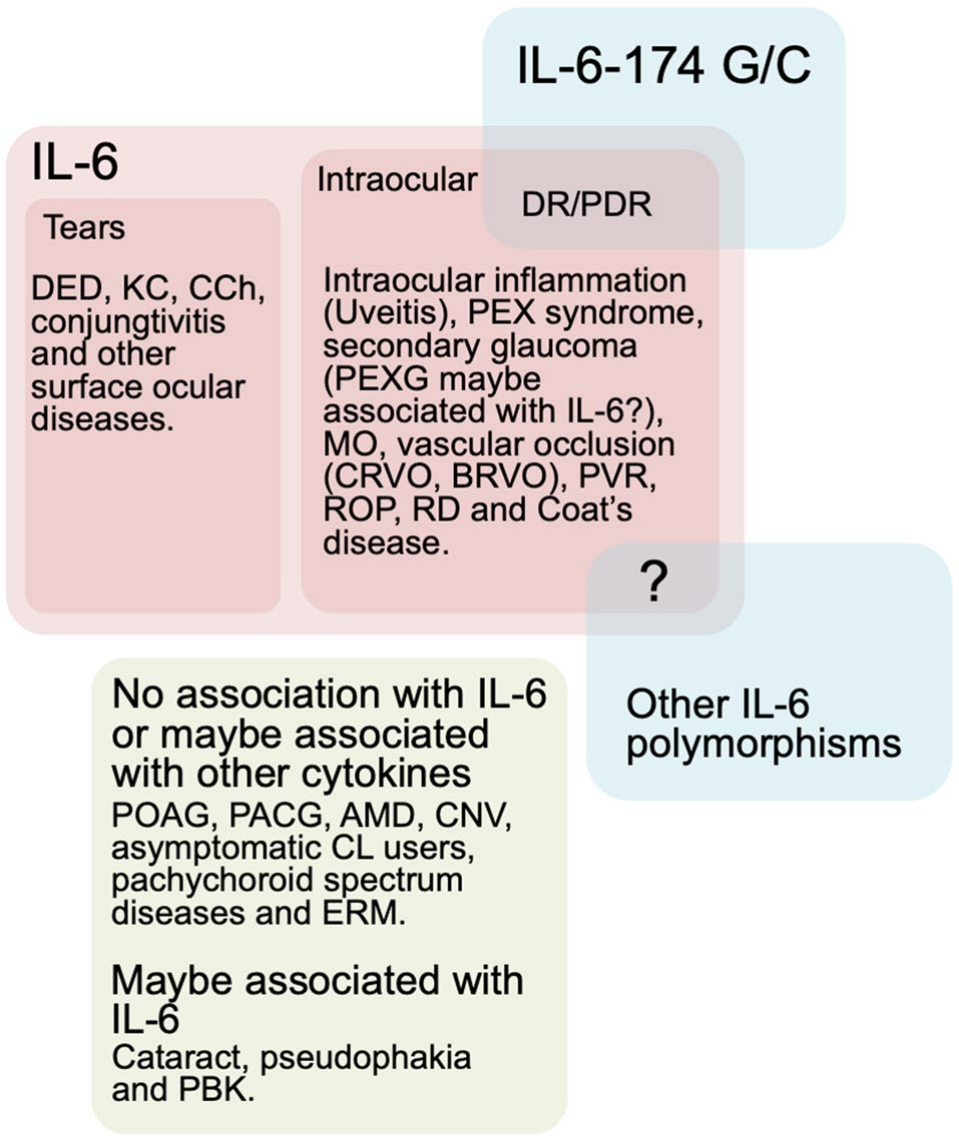
Summary of meta-analysis between IL-6 and ophthalmic diseases. *AMD* age-related macular degeneration, *BRVO* branch retinal vein occlusion, *CCh* conjunctivochalasis, *CL* contact lens, *CNV* choroidal neovascularization, *CRVO* central retinal vein occlusion, *KC* keratoconus, *MO* macular oedema, *DR* diabetic retinopathy, *ERM* epiretinal membrane, *PACG* primary angle-closure glaucoma, *PBK* pseudophakic bullous keratopathy, *PDR* proliferative diabetic retinopathy, *PEX* pseudoexfoliation syndrome, *POAG* primary open-angle glaucoma, *PVR* proliferative vitreoretinopathy, *RD* retinal detachment, *ROP* retinopathy of prematurity, *RP* retinitis pigmentosa.

Our study demonstrated that higher levels of IL-6 were associated with the overall risk of ocular diseases (SMD = 1.41, 95% CI 1.24–1.58, P < 0.00001). Moreover, high concentrations of IL-6 were found specifically in several ocular diseases, such as secondary glaucoma, PEX syndrome, surface ocular and intraocular inflammation, MO, CRVO, BRVO, PVR, ROP and RD, but not to POAG, PACG, AMD, CNV, asymptomatic CL users, pachychoroid spectrum diseases, ERM, cataract, pseudophakia and PBK (summarized in Fig. 8). Our analysis indicate that the upregulation of IL-6 levels in these specific ocular diseases are not necessarily associated with IL-6-174 G/C (rs1800795) polymorphism, therefore implying the potential modulating roles of other single nucleotide polymorphisms (SNPs) in the promoter region of IL-6. In fact, several studies have shown associations between rs1800796, rs1800797, and rs1524107, with surface ocular diseases and glaucoma^27,246,247^. Therefore, it may be useful for future studies to investigate the relationship between IL-6 genetic variants with intraocular IL-6 levels in ophthalmic cases, because it may provide more insight into the specific role of IL-6 in the ocular disease and provide more clarity or precision to the association.

The IL-6 levels tend to be high in glaucoma. However, only secondary glaucoma exhibited significantly increased IL-6 levels in this analysis, and no evidence of increase was found in POAG or PACG. This is possibly due to secondary glaucoma being caused by underlying diseases that are associated with a high IL-6 level, including PEX syndrome, intraocular inflammation, DR, ROP and RD^248^. And in regard to the lack of IL-6 increase in primary glaucoma, it is interesting to note previous reports showing that soluble IL-6 receptor (sIL-6R) but not IL-6 is elevated in the aqueous humour of POAG^49^. This interaction between IL-6 and sIL6R is known to exhibit the inhibitory action of TGF-β-induced up-regulation of fibrotic changes in vitro^49^, suggesting that trans-signalling of IL-6 could be a potential target of POAG treatment. Despite the fact that IL-6 may not directly contribute to OAG pathogenesis, studies confirmed an elevated level of TNFα and IL-8 in the OAG eye^249,250^, which emphasizes that the inflammatory process is involved in the OAG progression.

Although both CRVO and BRVO groups displayed high intraocular IL-6 levels, the increase was higher in CRVO than BRVO. Previous studies measuring other parameters such as ischaemic index and aqueous vascular endothelial growth factor concentration, also showed higher results in CRVO compared to BRVO^251,252^, thereby implicating the presence of more aggressive pathogenesis in CRVO. In terms of IL-6 involvement in RVO, IL-6 expression was found to be essential in modulating vascular inflammation through the upregulation of retinal vascular endothelial growth factor (VEGF)^253^, indicating an indirect role towards increased endothelial permeability via VEGF. Thus, as a consequence, the prognosis of CRVO is less favourable than BRVO. Nonetheless, further detail on the interaction between IL-6-VEGF and its implication on the initiation of retinal vascular disease requires more investigation.

Intraocular inflammation in this study was mainly focused on uveitis. For the analysis, studies evaluating intraocular IL-6 levels from uveitis patients were classified based on the aetiology^254^. Regardless of aetiology, this report clearly demonstrated that intraocular IL-6 levels were significantly increased in uveitis. Although a majority of previous investigations have shown higher serum IL-6 levels among uveitis patients than healthy controls, results of ocular fluid observations are less consistent^255,256^. In addition to the intraocular IL-6 levels, it was also confirmed that tear levels of IL-6 was higher among surface ocular diseases, including conjunctivitis, dry eye disease (DED), keratoconus (KC) and keratitis. Hence, this reflects that inhibition of local immune reaction during active inflammation may be critical for the management of ocular inflammation diseases.

Macular oedema is a leading cause of vision loss and represents an important clinical and public health problem. MO encompasses a pathological condition with intra- or subretinal fluid accumulation in the central retina^257^. Although a variety of pathological conditions could lead to the development of MO, this article will focus on the three main causes: DR, RVO and uveitis. The exact mechanisms of MO remain unclear, but one possible explanation is the involvement of the inflammatory process which has been considered critical towards increasing vascular permeability and subsequent breakdown of the blood-retinal barrier (BRB)^258,259^ . In this study, intraocular levels of IL-6 was significantly higher in DMO than non-DMO/RVO. On closer examination, it was found that patients with MO secondary to CRVO or BRVO showed a higher IL-6 concentration than patients with CRVO or BRVO alone. Indeed, the administration of IL-6 disrupts the integrity of human retinal pigment epithelial cells (ARPE-19) leading to increased cellular permeability concomitant with a low trans endothelial electrical resistance (TEER)^260^. This indicates the potential use of IL-6 as a biomarker and therapeutic target of retinal vascular diseases. To date, several studies and clinical trials utilizing the human anti-IL-6R antibody (Tocilizumab and Sarilumab) for ocular diseases have been introduced^261–267^. A phase II clinical trial for Tocilizumab (clinicaltrials.gov identifier NCT03554161) in the treatment of refractory Behcet's uveitis is also enrolling patients. Together, both treatments have shown clinical benefit in the management of non-infectious uveitis and refractory uveitis-related MO.

Although the number of pooled studies were relatively small, increased IL-6 level was also observed in RD, PVR, ROP and Coats’ disease. All these conditions share common similarity in terms of subretinal fluid accumulation. The possible role of IL-6 lies in its ability to promote vascular leakage in the retina, which may further escalate the inflammatory response within the eye. An interesting finding has indicated that trans-signalling IL-6 pathways predominantly occur in the ocular fluid of the RD model^268^. Other studies have confirmed significantly higher IL-6 concentration in the PVR than RD eye^269,270^. Overall, these results support the findings of this study, confirming the important role of IL-6 in the progression and severity of ocular diseases.

There was no evidence of IL-6 upregulation in AMD, CNV and pachychoroid spectrum diseases. However, IL-8 + 781 C/T (rs2227306) polymorphism in parallel with elevated IL-8 levels were associated with wet AMD^271^. Intraocular concentrations of IL-6 and IL-8 have been reported to be closely associated with the volume of MO and size of active CNV^175,182^. Moreover, IL-6 and IL-8 are significantly upregulated in the chronic CSC group compared with the acute CSC or PNV groups^272^. Therefore, further studies are needed to elucidate the role of IL-6 in the aetiology of AMD, CNV and pachychoroid spectrum diseases.

The strength of this study is on the large number of eligible studies collected for the meta-analysis, deeming the results presented as more conclusive. Thus, IL-6 should be considered as a promising marker and alternative approach to determine the presence of ophthalmic disorders. However, the analysis performed on several ocular disease subgroups used relatively small sizes, and so further studies are still needed to confirm these results. Several limitations of this study includes the limited availability of published papers on IL-6 polymorphism, which may lessen the precision of the effect estimate. Secondly, since ocular disease aetiology are multifactorial, other factors including genetic, hormonal, and environmental status need to be examined. Considering the above-mentioned limitations, these findings should be interpreted with caution.

In summary, this present meta-analysis suggests that IL-6-174 G/C polymorphism does not predispose patients to the ocular disease, but the intraocular IL-6 concentrations are related to the specific manifestations of the ophthalmic diseases. Another important point to note is that the GC genotype of IL-6-174 G/C polymorphism may serve as a potential biomarker of PDR. However, due to the complexity of PDR aetiologies and pathogenesis, multi-modality assessments combining genetic analysis with retinal imaging as well as non-genetic predispositions and other related biomarkers need to be performed in order to evaluate the risk and progression of DR. Overall, this study supports the notion that IL-6 plays a pivotal role in the pathogenesis of ophthalmic disorders. It is expected that more studies will become available to verify this conclusion.

## Methods

### Search strategy and study selection

The current meta-analysis was performed according to the Preferred Reporting Items for Systematic Reviews and Meta-Analysis (PRISMA) guideline^273^. A literature search was conducted from PubMed, Scopus and Web of Science databases up to October 2019 using the following keywords or terms, and their combination such as “IL-6 or interleukin-6”, “IL-6-174 G/C”, “cytokine”, “inflammation”, “polymorphism” and “eye disease/ophthalmology”. No language restrictions were imposed. In addition, reference lists of retrieved articles were manually screened to identify additional or potentially missing eligible studies. Studies were included based on fulfilment of the following criteria: (1) case–control study evaluating the association of IL-6-174 G/C or intraocular IL-6 levels with ocular disease; (2) genotype or allele frequencies or distributions in both case and control groups were provided; (3) IL-6 levels were quantified from aqueous or vitreous humour samples utilising ELISA (enzyme-linked immunosorbent assay) or multiplex bead immunoassay or immunoarray. If the level of IL-6 was not presented as mean and standard deviation (SD), estimated values were calculated as a previously described^274,275^. Animal studies, case reports, reviews, abstracts and incomplete studies were excluded. Two investigators (Z.U. and G.S.) independently selected the studies for final inclusion on the basis of these criteria. Disagreements between the two investigators were resolved and achieved after further discussion with the other investigators (B and L.W.)

### Data extraction

Data were extracted as follows: (1) name of the first author; (2) year of publication; (3) country of origin; (4) number of cases and controls; (5) age; (6) number of genotypes in cases and controls and (7) IL-6 levels in aqueous or vitreous humour from cases and controls.

### Data synthesis and analysis

Meta-analysis for IL-6 gene polymorphism was performed for two or more studies. Genotypic frequency of IL-6 gene polymorphism was tested for deviation from the Hardy–Weinberg equilibrium (HWE) in the control subjects. The genetic association was assessed using different genetic models, including allelic (a vs. A), recessive (aa vs. Aa + AA), dominant (aa + Aa vs. AA), over dominant (Aa vs. aa + AA), homozygous (aa vs. AA), heterozygous (Aa vs. AA), and Aa vs. aa models. The association between IL-6-174 G/C polymorphism with the ocular disease was calculated by the pooled OR and 95% CI. Z test was assessed to evaluate the significance of the pooled effect size. Heterogeneity among studies was evaluated using Q test and I^2^ statistic. A significant Q-statistic (P < 0.10) indicated heterogeneity across studies. The I^2^ values indicated no (0–24.9%), low (25–49.9%), moderate (50–74.9%) or high (75–100%) heterogeneity. The random-effect model (REM) was used if heterogeneity existed; otherwise, the fixed-effect model (FEM) was used^250,276–287^. Subgroup analysis was stratified by type of disease and ethnicity or racial descent.

Meta-analysis to evaluate IL-6 levels in the ocular disease was performed by REM to allow heterogeneity. Pooled SMD with 95% CI was used to assess the IL-6 levels between the patients with ocular disease and the controls. Subgroup and meta-regression analyses were also performed to investigate potential sources of heterogeneity. A sensitivity test was performed by sequentially omitting one study each time to evaluate the stability of the results. Begg’s funnel plots and Egger’s regression test were used to investigate the publication bias within studies. When a publication bias was detected, trim and fill analysis was performed to assess the number of missing studies. The Newcastle–Ottawa Scale (NOS) was utilised to assess the quality of case–control studies^288^ (Supplemental Table S9), with a maximum score of 9 for each study. A study scoring than 5 was judged at a high risk of bias^289^. All analyses were performed using MetaGenyo^290^, RevMan ver 5.3, Meta-Essential 1.4^291^, and Open Meta-Analyst^292^. Statistical tests were 2-sided and used a significance threshold of p < 0.05.

## Supplementary information

Supplementary Information.

## Data Availability

All data generated or analysed are included in this article.
