# Supplementary material for: The role of IL-6-174 G/C polymorphism and intraocular IL-6 levels in the pathogenesis of ocular diseases: a systematic review and meta-analysis
Source: Sci Rep. 2020 Oct 15;10:17453. doi: 10.1038/s41598-020-74203-9 (PMC7566646; doi:10.1038/s41598-020-74203-9)
Supplement: Supplementary file 1 — Supplementary Information. [file 41598_2020_74203_MOESM1_ESM.pdf]

Supplemental materials

The role of IL-6-174 G/C polymorphism and intraocular IL-6 levels in the pathogenesis of ocular diseases: a systematic review and meta-analysis

Zulvikar Syambani Ulhaq[1]*, Gita Vita Soraya[2], Budu[3], Lely Retno Wulandari[4].

[1]Department of Biochemistry, Faculty of Medicine and Health Sciences, Maulana Malik Ibrahim State Islamic University of Malang, Batu, East Java, Indonesia.

[2]Department of Biochemistry, Faculty of Medicine, Hasanuddin University, Makassar, South Sulawesi, Indonesia.

[3]Department of Ophthalmology, Faculty of Medicine, Hasanuddin University, Makassar, South Sulawesi, Indonesia.

[4]Department of Ophthalmology, Faculty of Medicine, Brawijaya University, Malang, East Java, Indonesia.

Number of supplementary materials: 10 supplemental tables, 13 supplemental figures, and 1 appendix

***Corresponding author:**

Zulvikar Syambani Ulhaq

Department of Biochemistry, Faculty of Medicine and Health Sciences, Maulana Malik Ibrahim State Islamic University of Malang, Batu, East Java, 65151, Indonesia.

Email: zulhaq@kedokteran.uin-malang.ac.id

Supplemental Table S1. Characteristics of individual studies for the association between IL-6-174 G/C polymorphism and ocular disease

No.	Study, year	Country	Gender (f/m)	Age[a]	Genotype distribution						P_{HWE}[b]
					Case			Control			
					GG	GC	CC	GG	GC	CC	
**Glaucoma**											
1	Lin et al, 2014	China	NTG: 123/117<br>Control: 140/122	NTG: 63.2 ± 10.2<br>Control: 61.3 ± 11.4	231	18	0	241	21	0	0.499
2	Wang et al, 2017	Taiwan	NTG: NA<br>Control: NA	NA	108	20	0	94	34	0	0.668
3	Wu et al, 2016	China	POAG: 89/131<br>Control: 102/146	POAG: 58.09 ±12.065<br>Control: 9.37 ± 11.92	219	1	0	245	1	0	0.975
4	Zimmermann et al, 2013	Austria	POAG: 111/80<br>Control: 191 (nd)	POAG: 71.5 ± 9.8<br>Control: 70.9 ± 9.6	63	98	30	67	87	37	0.362
**Retinopathy**											
5	Goverdhan et al, 2008	UK	AMD: 312/166<br>Control: 306/249	AMD: 78.8 ± 7.9<br>Control: 69.0 ± 9.7	156	223	83	178	270	105	0.884
6	Lu et al, 2016	China	PDR: 215 (nd)<br>Control: 207 (nd)	PDR: 58.3 ± 2.7<br>Control: 60.6 ± 3.9	64	118	33	78	87	42	0.553
7	Masood et al, 2007	UK	CMO+Control = 54 (nd)	NA	9	5		8	18		NA
8	Myśliwska et al, 2009	Poland	DR: 106/114<br>Control: 80/90	DR: 16.1 ± 1.7/ 18.8 ± 2.1<br>Control: 16.9 ± 5.2	12	24	0	51	68	51	0.009
9	Paine et al, 2012	India	PDR: 133/120<br>Control: 118/112	PDR: 52 ± 15.0<br>Control: 54 ± 2.0	138	103	12	140	92	8	0.125
10	Sanabria Ruiz-Colmenares et al, 2006	Spain	RD+PVR: 21/37<br>Control: 14/32	RD+PVR: 62.1/ 58.1<br>Control: 36.9	28	19	11	16	26	4	0.148
**Ocular and Introcular inflammation**											
11	Anvari et al, 2009	Iran	Graves' ophthalmology: 34/16<br>Control: 140 (nd)	NA	29	17	28	42	93	4	0.000
12	Bednarczuk et al, 2004	Poland	Graves' ophthalmology: 108 (nd)<br>Control: 186 (nd)	Graves' ophthalmology: 21/37<br>Control: 14/32	35	55	21	58	101	27	0.110

No.	Study, year	Country	Gender (f/m)	Age[a]	Genotype distribution						P_{HWE}[b]
					Case			Control			
					GG	GC	CC	GG	GC	CC	
13	Carnt et al, 2012	Australia	Contact lens keratitis: 90 (nd)<br>Control: 185 (nd)	NA	36	48	16	66	85	34	0.473
14	Cordeiro et al, 2013	Brazil	Toxoplasmic retinochoroiditis: 58/39<br>Control: 42/41	Toxoplasmic retinochoroiditis: 32.2 ± 12.7<br>Control: 34.71 ± 10.45	69	22	6	74	4	5	0.000
15	Dilek et al, 2009	Turkey	Ocular Behcet's disease: 97 (nd)<br>Control: 122 (nd)	NA	54	39	4	75	37	10	0.091
16	Ewald et al, 2015	Austria	Uveitis: 97/110<br>Control: 69/128	Uveitis: 44.81 ± 14.70/ 30.82 ± 16.66<br>Control: 35.56 ± 12.10/37.75 ± 4.02	59	126	33	75	93	29	0.984
17	Gedvilaite et al, 2019	Lithuania	Optic neuritis: 42/21<br>Control: 480/274	Optic neuritis: 34<br>Control: 53	33	27	12	170	373	211	0.833
18	Na et al, 2011	Korea	non-Sjogren dry eye: 251 (nd)<br>Control: 109 (nd)	NA	251	0	0	108	1	0	0.961
19	Sen et al, 2011	India	Eales' disease: 24/97<br>Control: 40/183	Eales' disease: 30.6 ± 10.7<br>Control: 32.26 ± 9.6	91	25	5	135	75	13	0.549
20	Taalat et al, 2014	Egypt	Ocular Behcet's disease: 16/71<br>Control: 96 (nd)	Ocular Behcet's disease: 34.37 ± 10.36<br>Control: NA	47	8	0	76	21	0	0.231
**Retinal vascular occlusion**											
21	Steinbrugger et al, 2009	Austria	RVO: 228/170<br>Control: 206/149	RVO: 67.1 ± 11.0<br>Control: 68.3 ± 13.8	130	197	71	115	174	66	0.989
22	Weger et al, 2004	Austria	RAO: 77/105<br>Control: 135/172	RAO: 69.1 ± 11.3<br>Control: 70.9 ± 11.4	64	99	19	107	139	61	0.197

No.	Study, year	Country	Gender (f/m)	Age[a]	Genotype distribution						P_{HWE}[b]
					Case			Control			
					GG	GC	CC	GG	GC	CC	
**Congenital eye disorders**											
**Hereditary stromal corneal dystrophies**											
23	Kucherenko et al, 2013	Kyiv	NA	NA	12	29	30	30	39	19	0.350

[a]Age was expressed as mean or mean ± SD, or otherwise indicated, [b]P for Hardy–Weinberg equilibrium (HWE) test in controls.

AMD, age-related macular degeneration; CMO, cystoid macular oedema; DR, diabetic retinophathy; NTG, normal tension glaucoma, NA, not available; ND, not define; POAG, primary open-angle glaucoma; PDR, proliferative diabetic retinopathy; PVR, proliferative vitreoretinopathy; RAO, retinal artery occlusion; RD, retinal detachment; RVO, retinal vein occlusion.

Supplemental Table S2. Meta-analysis for the association between IL-6-174 G/C polymorphism and ocular disease

Genetic model	Group	Number of studies	Test of association[a]				Test of heterogeneity		P Egger's test
			OR	95% CI	P	Model	P	I^2 (%)	
Allele model (C vs. G)	Overall* (1a)	22	0.96	0.84 – 1.12	0.649	Random	0.0001	67.83	0.686
	Overall** (1b)	19	0.94	0.82 – 1.08	0.372	Random	0.0004	60.18	0.423
	Glaucoma (2)	4	0.87	0.69 – 1.11	0.265	Fixed	0.4082	0	0.682
	Retinopathy* (3a)	5	0.97	0.86 – 1.10	0.645	Fixed	0.1004	48.52	0.485
	Retinopathy** (3b)	4	1.01	0.89 – 1.15	0.887	Fixed	0.6841	0	0.657
	Diabetic retinopathy* (4)	3	0.91	0.61 – 1.34	0.627	Random	0.0226	73.60	0.166
	PDR (5)	2	1.10	0.90 – 1.35	0.331	Fixed	0.6873	0	NA
	Ocular and Intraocular inflammation* (6a)	10	0.98	0.7 – 1.32	0.890	Random	0.0001	77.59	0.719
	Ocular and Intraocular inflammation** (6b)	8	0.83	0.62 – 1.11	0.216	Random	0.0009	71.64	0.296
	Graves' ophthalmology* (7)	2	1.34	0.86 – 2.09	0.191	Random	0.0926	64.65	NA
	Ocular Behcet's disease (8)	2	0.95	0.64 – 1.40	0.782	Random	0.3218	0	NA
	Retinal ascular occlusion (9)	2	0.92	0.78 – 1.08	0.283	Fixed	0.2868	11.86	NA
	Asian** (10)	8	0.88	0.69 – 1.12	0,311	Random	0.0837	44.25	0.222
	Caucasian** (11)	10	0.98	0.82 – 1.17	0,831	Random	0.0002	71.62	0.732
Recessive model (CC vs. GC+GG)	Overall* (1a)	17	1.02	0.74 – 1.41	0.871	Random	0.0001	74.19	0.679
	Overall** (1b)	14	0.91	0.72 – 1.14	0.435	Random	0.0176	49.78	0.736
	Glaucoma (2)	4	0.77	0.46 – 1.32	0.347	Fixed	1	0	0.001
	Retinopathy* (3a)	5	0.94	0.55 – 1.61	0.841	Random	0.0412	59.81	0.797
	Retinopathy** (3b)	4	0.94	0.73 – 1.21	0.632	Fixed	0.2200	32.05	0.299
	Diabetic retinopathy* (4)	3	0.64	0.21 – 1.98	0.439	Random	0.0324	70.84	0.638
	PDR) (5)	2	0.84	0.54 – 1.30	0.435	Fixed	0.1837	43.43	NA
	Ocular and Intraocular inflammation* (6a)	8	1.16	0.61 – 2.22	0.642	Random	0.0001	80.67	0.710
	Ocular and Intraocular inflammation** (6b)	6	0.85	0.64 – 1.13	0.281	Fixed	0.2937	18.44	0.603
	Graves' ophthalmology* (7)	2	5.10	0.36 – 72.26	0.227	Random	0.0001	94.30	NA
	Ocular Behcet's disease (8)	2	0.48	0.15 – 1.59	0.230	Fixed	1	NA	NA
	Retinal ascular occlusion (9)	2	0.69	0.35 – 1.37	0.289	Random	0.0378	76.82	NA
	Asian** (10)	4	0.77	0.52 – 1.13	0.189	Fixed	0.4671	0	0.874
	Caucasian** (11)	10	0.95	0.72 – 1.25	0,737	Random	0.0071	60.21	0.474

Genetic model	Group	Number of studies	Test of association[a]				Test of heterogeneity		P Egger's test
			OR	95% CI	P	Model	P	I^2 (%)	
Dominant model (CC+GC vs. GG)	Overall* (1a)	22	0.95	0.78 – 1.15	0.631	Random	0.0001	65.37	0.616
	Overall** (1b)	19	0.93	0.76 – 1.13	0.490	Random	0.0001	64.24	0.394
	Glaucoma (2)	4	0.87	0.64 – 1.18	0.374	Fixed	0.2585	25.52	0.804
	Retinopathy* (3a)	5	1.03	0.86 – 1.24	0.678	Fixed	0.2120	31.42	0.636
	Retinopathy** (3b)	4	1.04	0.87 1.25	0.602	Fixed	0.1340	46.23	0.791
	Diabetic retinopathy* (4)	3	1.22	0.95 – 1.57	0.123	Fixed	0.4852	0	0.535
	PDR (5)	2	1.27	0.97 – 1.66	0.076	Fixed	0.4653	0	NA
	Ocular and Intraocular inflammation* (6a)	10	0.88	0.58 – 1.34	0.562	Random	0.0001	78.89	0.804
	Ocular and Intraocular inflammation** (6b)	8	0.79	0.50 – 1.23	0.303	Random	0.0001	77.81	0.461
	Graves' ophthalmology* (7)	2	0.84	0.57 – 1.23	0.364	Fixed	0.3366	0	NA
	Ocular Behcet's disease (8)	2	1.05	0.66 – 1.66	0.230	Fixed	1	NA	NA
	Retinal ascular occlusion (9)	2	0.99	0.78 – 1.25	0.916	Fixed	0.9954	0	NA
	Asian** (10)	8	0.89	0.63 – 1.27	0,544	Random	0.0133	60.50	0.355
	Caucasian** (11)	10	0.97	0.74 – 1.26	0,833	Random	0.0002	71.53	0.993
Overdominant model (GC vs. CC+GG)	Overall* (1a)	22	0.95	0.75 – 1.20	0.678	Random	0.0001	77.75	0.394
	Overall** (1b)	19	0.97	0.82 – 1.16	0.762	Random	0.0008	57.98	0.085
	Glaucoma (2)	4	0.95	0.71 – 1.28	0.738	Fixed	0.1225	48.15	0.852
	Retinopathy* (3a)	5	1.17	0.77 – 1.80	0.464	Random	0,0008	79.01	0.838
	Retinopathy** (3b)	4	1.01	0.68 – 1.51	0.943	Random	0.0062	75.76	0.621
	Diabetic retinopathy* (4)	3	1.62	1.00 – 2.61	0.047	Random	0.0438	68.04	0.366
	PDR (5)	2	1.34	1.03 – 1.75	0.028	Fixed	0.1208	58.45	NA
	Ocular and Intraocular inflammation* (6a)	10	0.82	0.50 – 1.36	0.446	Random	0.0001	85.19	0.687
	Ocular and Intraocular inflammation** (6b)	8	0.88	0.62 – 1.25	0.503	Random	0.0058	64.84	0.275
	Graves' ophthalmology* (7)	2	0.35	0.07 – 1.91	0.228	Random	0.0001	94.39	NA
	Ocular Behcet's disease (8)	2	1.04	0.43 – 2.55	0.923	Random	0.0871	65.84	NA
	Retinal ascular occlusion (9)	2	1.16	0.93 – 1.46	0.193	Fixed	0.1454	52.82	NA
	Asian** (10)	8	0.93	0.63 – 1.39	0.752	Random	0.0028	67.78	0.353
	Caucasian** (11)	10	1.00	0.82 – 1.22	0.940	Random	0.0191	54.58	0.228

Genetic model	Group	Number of studies	Test of association[a]				Test of heterogeneity		P Egger's test
			OR	95% CI	P	Model	P	I^2 (%)	
Homozygous model (CC vs. GG)	Overall* (1a)	17	1.02	0.74 – 1.41	0.913	Random	0.0001	69.40	0.778
	Overall** (1b)	14	0.93	0.71 – 1.23	0.619	Random	0.0036	57.84	0.805
	Glaucoma (2)	4	0.86	0.48 – 1.56	0.624	Fixed	1	0	0.462
	Retinopathy* (3a)	5	0.96	0.72 – 1.26	0.753	Fixed	0.172	37.38	0.683
	Retinopathy** (3a)	4	0.99	0.75 – 1.31	0.922	Fixed	0.6624	0	0.055
	Diabetic retinopathy* (4)	3	0.81	0.28 – 2.33	0.696	Random	0.0578	64.93	0.493
	PDR (5)	2	1.09	0.67 – 1.76	0.738	Fixed	0.4019	0	NA
	Ocular and Intraocular inflammation* (6a)	8	1.05	0.55 – 2.01	0.880	Random	0.0001	78.07	0.786
	Ocular and Intraocular inflammation** (6b)	6	0.75	0.43 – 1.30	0.320	Random	0.0136	65.11	0.736
	Graves' ophthalmology* (7)	2	3.43	0.46 – 25.84	0.231	Random	0.0028	88.85	NA
	Ocular Behcet's disease (8)	2	0.56	0.17 – 1.86	0.341	Fixed	1	NA	NA
	Retinal ascular occlusion (9)	2	0.78	0.55 – 1.10	0.159	Fixed	0.1066	61.59	NA
	Asian** (10)	4	0.91	0.60 – 1.37	0.661	Fixed	0.4586	0	0.616
	Caucasian** (11)	10	0.95	0.67 – 1.35	0.793	Random	0.0009	68.13	0.550
Heterozygous model (GC vs. GG)	Overall* (1a)	22	0.95	0.76 – 1.19	0.650	Random	0.0001	71.14	0.520
	Overall** (1b)	19	0.94	0.78 – 1.16	0.610	Random	0.0001	62.66	0.248
	Glaucoma (2)	4	0.90	0.65 – 1.23	0.494	Fixed	0.1882	37.31	0.795
	Retinopathy* (3a)	5	1.09	0.78 – 1.52	0.625	Random	0.0375	60.70	0.896
	Retinopathy** (3b)	4	1.03	0.71 – 1.51	0.862	Random	0.0235	68.36	0.681
	Diabetic retinopathy* (4)	3	1.35	1.04 – 1.75	0.026	Fixed	0.4127	0	0.710
	PDR (5)	2	1.33	1.01 – 1.76	0.045	Fixed	0.1937	40.79	NA
	Ocular and Intraocular inflammation* (6a)	10	0.84	0.51 – 1.37	0.480	Random	0.0001	82.42	0.775
	Ocular and Intraocular inflammation** (6a)	8	0.81	0.52 – 1.27	0.375	Random	0.0002	75.70	0.386
	Graves' ophthalmology* (7)	2	0.50	0.15 – 1.66	0.257	Random	0.0063	86.58	NA
	Ocular Behcet's disease (8)	2	1.02	0.44 – 2.35	0.964	Random	0.1089	61.09	NA
	Retinal ascular occlusion (9)	2	1.07	0.83 – 1.38	0.590	Fixed	0.5115	0	NA
	Asian** (10)	8	0,92	0.62 – 1.36	0.691	Random	0.0049	65.60	0.403
	Caucasian** (11)	10	0,98	0.76 – 1.26	0.876	Random	0.0015	66.33	0.652

Genetic model	Group	Number of studies	Test of association[a]				Test of heterogeneity		P Egger's test
			OR	95% CI	P	Model	P	I^2 (%)	
GC vs. CC	Overall* (1a)	22	1.02	0.92 – 1.13	0.670	Fixed	0.7570	0	0.685
	Overall** (1b)	19	1.03	0.92 – 1.14	0.568	Fixed	1	0	0.348
	Glaucoma (2)	4	1.06	0.63 – 1.78	0.706	Fixed	0.9960	0	0.312
	Retinopathy* (3a)	5	1.06	0.84 – 1.33	0.515	Fixed	0.4330	0	0.743
	Retinopathy** (3b)	4	1.02	0.77 – 1.34	0.829	Fixed	0.7440	0	0.470
	Diabetic retinopathy* (4)	3	1.15	0.67 – 1.97	0.268	Fixed	0.3070	15.41	0.330
	PDR (5)	2	1.07	0.19 – 5.97	0.625	Fixed	0.5210	0	NA
	Ocular and Intraocular inflammation* (6a)	10	0.96	0.78 – 1.18	0.637	Fixed	0.3880	5.81	0.962
	Ocular and Intraocular inflammation** (6b)	8	1.03	0.82 – 1.29	0.794	Fixed	1	0	0.888
	Graves' ophthalmology* (7)	2	0.62	0.00 – 130.98	0.257	Random	0.0030	78.82	NA
	Ocular Behcet's disease (8)	2	1.12	0.03 – 36.96	0.688	Fixed	0.8310	0	NA
	Retinal ascular occlusion (9)	2	1.08	0.27 – 4.27	0.459	Fixed	0.4330	0	NA
	Asian** (10)	8	1.06	0.82 – 1.36	0.604	Fixed	0.9990	0	0.656
	Caucasian** (11)	10	1.02	0.89 – 1.17	0.713	Fixed	0.9540	0	0.176

[a]If the P value for Q-statistic was < 0.10 or the I^2 value ≥ 50% random-effect model was used, otherwise fixed-effect model was adopted. OR, odds ratio; CI, confidence interval; HWE, Hardy–Weinberg equilibrium; NA, not available; PDR, proliferative diabetic retinopathy. *all relevant articles were included; **articles that deviate from the HWE were excluded.

Supplemental Table S3. Summary of the studies for IL-6 levels in the intraocular fluid (aqueous and vitreous humor) between patients with ocular disease and the control subjects

No.	Study, year	Country	Age[a]	Case		Control	
				n	IL-6 (pg/mL, mean ± SD)	n	IL-6 (pg/mL, mean ± SD)
**Glaucoma**							
**1. Primary open-angle glaucoma (POAG)**							
1	Borkenstein et al, 2013	Austria	POAG: 76.4 ± 8.9<br>Control: 77.3 ± 9.4	25	9.3 ± 23.7	29	55.3 ± 94.4
2	Chua et al, 2012*	Singapore	POAG: 69.9 ± 7.6<br>Control: 66.5 ± 11.8	26	8.425 ± 7.0	23	4.615 ± 3.5
3	Engel et al, 2014 (a)	Germany	POAG: 65.5 ± 8.6<br>Control: 74.0 ± 10.3	13	14.71 ± 14.33	12	143.26 ± 168.70
4	Engel et al, 2014 (b)	Germany	POAG+S: 71.7 ± 8.8<br>Control: 74.0 ± 10.3	19	182.50 ± 309.13	12	143.26 ± 168.70
5	Freedman and Iserovich, 2013	USA	POAG: 74<br>Control 63	23	94.0 ± 48.0	13	19.0 ± 16.0
6	Ghanem et al, 2011	Egypt	POAG: 61.51 ± 4.91<br>Control: 60.35 ± 4.71	30	85.1 ± 45.5	35	34.6 ± 12.4
7	Inoue-Mochita et al, 2018	Japan	POAG: 62.4 ± 11.2<br>Control: 80.5 ± 5.1	22	15.9 ± 54.7	17	34.6 ± 44.8
8	Khalef et al, 2017 (a)	Egypt	POAG: 65<br>Control: 63	30	15.1 ± 1.5	15	64.3 ± 22.1
9	Kutchey et al, 2010*	USA	POAG: 71.3 ± 9.9<br>Control: 68.0 ± 7.3	20	4.3 ± 3.5	29	21.85 ± 20.5
10	Niu, 2018 (a)	China	POAG: 50.15 ± 19.12<br>Control: 54.58 ± 12.28	58	318.93 ± 45.71	56	227.53 ± 38.28
11	Ohira et al, 2015 (a)	Japan	POAG: 68.9 ± 10.9<br>Control: 78.3 ± 6.7	36	10.0 ± 22.6	68	35.6 ± 109.5
12	Sorkhabi et al, 2010	Iran	POAG: 71.1 ± 6.3<br>Control: 70.8 ± 6.5	20	10.84 ± 3.37	25	5.79 ± 2.63
13	Takai et al, 2012 (a)	Japan	POAG: 73.4 ± 11.5<br>Control: 75.2 ± 5.3	20	15.1 ± 19.1	21	64.3 ± 112.2
14	Ten Berge et al, 2019*	Netherland	POAG: 71 ± 18.25<br>Control: 57.25 ± 16.5	28	50.75 ± 47.75	22	39.0 ± 37.5
15	Zenkel et al, 2010 (a)	Germany	POAG: 69.5 ± 5.9<br>Control: 75.1 ± 4.8	14	59.0 ± 39.0	14	52.0 ± 34.0
**2. Primary angle-closure glaucoma (PACG)**							
16	Du et al, 2016*	China	PACG/Control: 63.80 ± 6.76	18	520.24 ± 867.5	18	0.35 ± 0.455
17	Duvesh et al, 2017*	India	PACG: 60.9<br>Control: 60.7	19	347.89 ± 332.92	14	58.44 ± 31.16
18	Liu et al, 2017	China	PACG: 62.1 ± 6.5<br>Control: 70.2 ± 7.7	20	269.8 ± 648.2	15	8.2 ± 9.1
19	Wang et al, 2018	China	PACG: 57.3 ± 10.8<br>Control: 74.0 ± 5.5	40	1.7 ± 3.6	24	5.6 ± 28.9
**3. Secondary glaucoma**							
20	Khalef et al, 2017 (b)	Egypt	PEXG: 67<br>Control: 63	30	23 ± 1.4	15	64.3 ± 22.1
21	Kovacs et al, 2015	USA	NVG: 68.5 ± 9.5<br>Control: 70.1 ± 9.9	12	2858.4 ± 4111.6	29	17.3 ± 19.7
22	Li et al, 2012* (a)	Singapore	PSS, CMV-: 48.4 ± 13.2<br>Control: 66.3 ± 14.99	39	381 ± 373.5	23	4.6 ± 3.5
23	Li et al, 2012* (b)	Singapore	PSS, CMV+: 49.7 ± 14.3<br>Control: 66.3 ± 14.99	14	102.3 ± 95.75	23	4.6 ± 3.5

No.	Study, year	Country	Age[a]	Case n	Case IL-6 (pg/mL, mean ± SD)	Control n	Control IL-6 (pg/mL, mean ± SD)
24	Liu et al, 2017 (a)	China	Secondary glaucoma post silicone oil tamponade: 49.7 ± 14.3<br>Control: 66.3 ± 14.99	19	351.1 ± 28.4	39	254.4 ± 26.8
25	Niu, 2018 (b)	China	NVG: 53.74 ± 18.21<br>Control: 54.58 ± 12.28	64	670.15 ± 72.61	56	227.53 ± 38.28
26	Ohira et al, 2015 (b)	Japan	UG: 60.1 ± 14.8<br>Control: 78.3 ± 6.7	39	171.1 ± 317.8	68	35.6 ± 109.5
27	Rusnak et al, 2015*	Czech Republic	PDR+NVG: NA<br>Control: NA	11	2689.7 ± 2490.02	50	51.48 ± 48.29
28	Sarenac Vulovic et al, 2015 (a)	Serbia	PEXG: 77.2 ± 3.9<br>Control: 63.4 ± 4.2	30	424.86 ± 72.98	30	143.82 ± 22.41
29	Takai et al, 2012 (b)	Japan	PEXG: 79.4 ± 4.5<br>Control: 75.2 ± 5.3	23	105.1 ± 124.2	21	64.3 ± 112.2
30	Zenkel et al, 2010 (b)	Germany	PEXG: 76.7 ± 7.8<br>Control: 75.1 ± 4.8	14	50.0 ± 36.1	14	52.0 ± 34.0
**Pseudoexfoliation (PEX) syndrome**							
31	Garweg et al, 2017 (a)	Switzerland	Early PEX: 81.19<br>Control: 68.32	33	2.2 ± 0.8	20	4.1 ± 2.7
32	Garweg et al, 2017 (b)	Switzerland	Late PEX: 79.30<br>Control: 68.32	30	3.1 ± 1.5	20	4.1 ± 2.7
33	Garweg et al, 2017 (c)	Switzerland	Late PEX+Luxation: 84.24<br>Control: 68.32	10	118.6 ± 104.9	20	4.1 ± 2.7
34	Sarenac Vulovic et al, 2015 (b)	Serbia	Early PEX: 73.4 ± 6.5<br>Control: 63.4 ± 4.2	30	627.05 ± 65.69	30	143.82 ± 22.41
35	Sarenac Vulovic et al, 2015 (c)	Serbia	Late PEX: 73.4 ± 6.5<br>Control: 63.4 ± 4.2	30	162.86 ± 38.37	30	143.82 ± 22.41
36	Zenkel et al, 2010 (c)	Germany	Early PEX: 75.8 ± 8.1<br>Control: 75.1 ± 4.8	14	186.0 ± 99.0	14	52.0 ± 34.0
37	Zenkel et al, 2010 (d)	Germany	Late PEX: 75.5 ± 7.8<br>Control: 75.1 ± 4.8	14	57.0 ± 32.0	14	52.0 ± 34.0
**Ocular inflammation**<br>**1. Surface ocular inflammation**							
38	Aketa at el, 2017	Japan	Ocular surface disease: 62.4 ± 13.7<br>Control: 75.6 ± 7.0	14	55.3 ± 14.7	30	42.7 ± 1.5
39	Zhang et al, 2018	China	Fungal keratitis: 49. 30 ± 17.02<br>Control: 24.88 ± 12.12	10	6179.71 ± 1015.726	8	6.22 ± 7.55
**2. Intraocular inflammation**<br>**2a. Uveitis[a]**							
40	Banerjee et al, 2007* (a)	USA	Uveitis: NA<br>Control: NA	8	145 ± 1004.75	8	6 ± 6.25
41	Hernández Garfella et al, 2015	Spain	Uveitis: 47.83 ± 11.37<br>Control: 72.16 ± 5.76	6	109.8 ± 88.1	12	333.38 ± 274.83
42	Petrinović-Doresić, et al 1999* (c)	Croatia	Exogenous uveitis: 54.25 ± 13.25<br>Control: 69.3 ± 5.25	14	2081.95 ± 1197.2	10	88.43 ± 63.33

No.	Study, year	Country	Age[a]	Case		Control	
				n	IL-6 (pg/mL, mean ± SD)	n	IL-6 (pg/mL, mean ± SD)
43	Sijssens et al, 2007 (a)	Netherland	Uveitis: 9.6<br>Control: 3.3	25	106 ± 3454.75	6	6.0 ± 1.5
44	Sijssens et al, 2007* (b)	Netherland	Uveitis: 9.41 ± 3.26<br>Control: NA	25	3509.25 ± 3454.75	6	4.5 ± 1.5
45	van Kooij et al, 2006*	Netherland	Uveitis: 50<br>Control: 66	31	3783.5 ± 3445.5	11	87 ± 52
46	Wakefield et al, 1995	USA	Uveitis: 45.8<br>Control: 55.3	10	135000 ± 2000	7	0.00 ± 0.00
**2b. Infectious**							
47	Ang et al, 2012* (a)	Singapore	TAU: 41.1 ± 20.9<br>Control: 66.3 ± 15.0	10	470.4 ± 276.8	23	3.4 ± 0.6
48	Curnow et al, 2005* (c)	UK	Herpes viral uveitis: 40.2<br>Control: 73.3	5	8082 ± 5796.5	12	324 ± 279.5
49	Murray et al, 1990 (a)	Netherland	TU: 57.9 ± 14.4<br>Control: 75.1 ± 4.8	8	19.23 ± 50.08	13	0.00 ± 0.00
50	Sauer et al, 2018*	France	Endophthalmitis: 73.2 ± 9.9<br>Control: 71.9 ± 12	46	12662.5 ± 5177.5	60	55.75 ± 24.25
51	Zenkel et al, 2010 (e)	Germany	Resolved herpetic uveitic: 57.9 ± 14.4<br>Control: 75.1 ± 4.8	14	193.0 ± 144.0	14	52.0 ± 34.0
**2c. Inflammatory**							
52	Ang et al, 2012* (b)	Singapore	IU: 55.8 ± 13.7<br>Control: 66.3 ± 15.0	15	21.8 ± 6.1	23	3.4 ± 0.6
53	Bae and Lee, 2012	Korea	Ocular behcet’s disease: 39.3 ± 11.9<br>Control: NA	7	212.84 ± 150.30	23	16.59 ± 31.42
54	Chen et al, 2014 (a)	China	Inflammatory cataract secondary to VKH: 47.10 ± 9.78<br>Control: 49.90 ± 10.59	10	22.96 ± 6.68	10	9.31 ± 6.10
55	Chen et al, 2014 (b)	China	Inflammatory cataract secondary to Behcet’s disease: 41.11 ± 7.22<br>Control: 49.90 ± 10.59	9	28.34 ± 17.8	10	9.31 ± 6.10
56	Curnow et al, 2005* (a)	UK	IU: 40.2<br>Control: 73.3	23	230054 ± 199997.5	12	324 ± 279.5
57	Curnow et al, 2005* (d)	UK	Behcet’s uveitis: 40.2<br>Control: 73.3	4	10129 ± 3155	12	324 ± 279.5
58	Murugeswari et al, 2008*	India	ED: 29.3 ± 6.1<br>Control: 61.8 ± 8.1	10	400.95 ± 300	10	0.00 ± 0.00
59	Murugeswari et al, 2014	India	ED: 31 ± 10<br>Control: 60.4 ± 8.2	5	74.48 ± 86.05	5	0.00 ± 0.00
60	Nagata et al, 2012	Japan	Sarcoid uveitis: 66.8 ± 2.2<br>Control: 69.5 ± 3.1	19	736 ± 276.5	5	13.8 ± 4.2
61	Petrinović-Doresić, et al 1999* (a)	Croatia	HLA-B27$^+$ anterior uveitis: 57.9 ± 14.4<br>Control: 69.3 ± 5.25	10	1023.9 ± 634.55	10	88.43 ± 63.33

No.	Study, year	Country	Age[a]	Case n	Case IL-6 (pg/mL, mean ± SD)	Control n	Control IL-6 (pg/mL, mean ± SD)
62	Petrinović-Doresić, et al 1999* (b)	Croatia	Endogenous idiopathic anterior uveitis: 67.1 ± 11.5 Control: 69.3 ± 5.25	13	157.1 ± 134.65	10	88.43 ± 63.33
63	Valentincic et al, 2011*	Netherland	IU: 36 Control: 40	36	6.6 ± 3.75	10	4.33 ± 1.38
**2d. Pseudo-uveitis**							
64	Aketa et al, 2017 (a)	Japan	Iris damage: 62.4 ± 13.7 Control: 75.6 ± 7.0	14	1696 ± 805	30	6.4 ± 0.9
65	Banerjee et al, 2007* (b)	USA	LIU: NA Control: NA	15	5000 ± 7926.25	8	6 ± 6.25
66	Nagarkatti-Gude et al, 2012	Netherland	Uveal melanoma: 57.9 ± 2.6 Control: NA	33	107.89 ± 66.25	9	38.53 ± 28.46
**2e. Ophthalmologic entities**							
67	Curnow et al, 2005* (b)	UK	FHC: 40.2 Control: 73.3	5	706.75 ± 631.75	12	324 ± 279.5
68	Kuiper et al, 2011*	Netherland	BSCR: 61 ± 13 Control: 73 ± 12	16	83.9 ± 73.05	11	7.1 ± 3.5
69	Murray et al, 1990 (b)	Netherland	FHC: 57.9 ± 14.4 Control: 75.1 ± 4.8	16	543 ± 1775	13	0.00 ± 0.00
70	Perez et al, 2004*	USA	Pars planitis: NA Control: NA	23	61.64 ± 43.25	8	10.6 ± 7
**Diabetic retinopathy (DR)** **1. DR[a]**							
71	Boss et al, 2017	USA	DR: 67.8 Control: 61.5	22	212.5 ± 179.6	28	43.3 ± 84.7
72	Chen et al, 2017	China	DR: 60.7 ± 7.6 Control: 61.1 ± 7.4	101	40.64 ± 16.52	51	23.00 ± 14.42
73	Chernykh et al, 2015	Russia	DR: 50.5 ± 3.2 Control: 53.5 ± 2.6	38	64.2 ± 14.6	25	32.8 ± 8.7
74	Cheung et al, 2012*	Singapore	DR: 67.4 ± 10.7 Control: 64.9 ± 17.5	27	505.2 ± 497	24	8.1 ± 7
75	Dong et al, 2013*	China	DR: 68.92 ± 8.12 Control: 65.71 ± 7.35	56	96.5 ± 62.75	68	65.75 ± 55.75
76	Feng et al, 2018 (a)	China	5 yr DR: 58.5 ± 8.9 Control: 58.4 ± 5.3	20	86.324 ± 1.54	20	18.221 ± 1.45
77	Feng et al, 2018 (b)	China	10 yr DR: 59.3 ± 7.2 Control: 58.6 ± 7.5	20	31.480 ± 1.35	20	16.080 ± 0.79
78	Funatsu et al, 2001	Japan	DR: 67.4 Control: 72.8	44	82.9 ± 93.7	16	13.6 ± 9.82
79	Gustavsson et al, 2012* (a)	Sweden	DR: 53.75 ± 14.25 Control: 63 ± 10.5	26	1129.5 ± 1105	27	435.25 ± 344
80	Oh et al, 2010	Korea	DR: NA Control: NA	38	15.48 ± 34.36	16	23.57 ± 39.10
81	Sin et al, 2013	Korea	DR+DMO: NA Control: NA	60	93.23 ± 110.44	10	2.083 ± 1.67
82	Suzuki et al, 2011	Japan	DR: 57.6 ± 13.6 Control: 70.2 ± 7.3	76	1512.24 ± 2314.35	10	188.24 ± 167.78
83	Yuuki et al, 2001	Japan	DVR: NA Control: NA	47	64.7 ± 12.8	22	12.8 ± 4.5
**1a. Proliferative diabetic retinopathy (PDR)**							
84	Abu el Asrar et al, 1992	Egypt	PDR: 51 Control: NA	13	43.3 ± 15.7	15	0.00 ± 0.00

No.	Study, year	Country	Age[a]	Case		Control	
				n	IL-6 (pg/mL, mean ± SD)	n	IL-6 (pg/mL, mean ± SD)
85	Adamiec-Mroczek and Oficjalska-Młyńczak, 2008	Poland	PDR: 63.97 ± 9.0<br>Control: 63.00 ± 14.58	19	78.76 ± 34.14	15	7.74 ± 6.94
86	Banerjee et al, 2007*	USA	PDR: NA<br>Control: NA	10	32 ± 171.5	8	6 ± 6.25
87	Bromberg-White et al, 2013	USA	PDR: 71 ± 7<br>Control: 58 ± 11	40	45.2 ± 73.68	29	6.9 ± 16.19
88	Canataroglu et al, 2005	Turkey	PDR: 56.1<br>Control: 41.1	14	53.44 ± 53.08	15	0.45 ± 0.79
89	Feng et al, 2018 (c)	China	5 yr PDR: NA<br>Control: 58.4 ± 5.3	9	91.434 ± 2.12	20	18.221 ± 1.45
90	Feng et al, 2018 (d)	China	10 yr PDR: NA<br>Control: 58.6 ± 7.5	10	35.987 ± 0.17	20	16.080 ± 0.79
91	Kauffmann et al, 1994	Netherland	PDR: 56.3 ± 3.9<br>Control: 63 ± 6.9	17	58 ± 93	11	40 ± 68
92	Koskela et al, 2013	Finland	PDR: 59.4 ± 14.3<br>Control: 66.6 ± 9.3	36	272.6 ± 956.1	16	4.0 ± 6.1
93	Kovacs et al, 2015 (a)	USA	PDR: 65.0 ± 13.6<br>Control: 70.1 ± 9.9	29	170.3 ± 370.5	29	17.3 ± 19.7
94	Loukovaara et al, 2017	Finland	PDR: 45.1 ± 3.1<br>Control: 64.27 ± 1.87	23	64.1 ± 59.1	15	167.3 ± 490.3
95	Mocan et al, 2006	Turkey	PDR: 59 ± 11<br>Control: 54 ± 15	8	755 ± 177	8	93 ± 151
96	Murugeswari et al, 2008* (a)	India	PDR: 54.9 ± 8.9<br>Control: 61.8 ± 8.1	25	451.48 ± 411.48	10	0.00 ± 0.00
97	Murugeswari et al, 2014 (a)	India	PDR: 54.2 ± 7.8<br>Control: 60.4 ± 8.2	10	115.67 ± 174.12	5	0.00 ± 0.00
98	Nakamura et al, 2003	Japan	PDR: 54.89 ± 12.1<br>Control: 61.69 ± 11.7	62	276.19 ± 13.9	50	96.79 ± 9.3
99	Rusnak et al, 2015* (a)	Czech Republic	PDR: NA<br>Control: NA	41	1163.94 ± 1143.35	50	51.48 ± 48.29
100	Rusnak et al, 2015* (b)	Czech Republic	PDR+VB: NA<br>Control: NA	11	88.23 ± 73.94	50	51.48 ± 48.29
101	Schoenberger et al, 2012	USA	PDR: 56 ± 10<br>Control: 69 ± 6	13	22.75 ± 24.5	13	4.25 ± 8.6
102	Shimura et al, 2009	Japan	PDR+PRP/control: 64.4 ± 5.9	14	520.3 ± 258.2	14	390.7 ± 150.5
103	Song et al, 2014 (a)	Korea	PDR: 49.2 ± 5.8<br>Control: 50.3 ± 4.6	12	34.69 ± 20.23	12	68.89 ± 207
104	Srividya et al, 2018	India	PDR: NA<br>Control: NA	7	55.20 ± 21.72	6	4.52 ± 1.37
105	Takahashi et al, 2016	Japan	PDR: NA<br>Control: NA	55	918.0 ± 1703.0	14	77.3 ± 278.0
106	Tsai et al, 2018	Germany	PDR: 63.1 ± 12.2<br>Control: 74.6 ± 8.7	17	50.40 ± 22.76	17	42.29 ± 10.94
107	Wu et al, 2017 (a)	China	PDR: 67<br>Control: 72	14	39.86 ± 28.79	10	7.10 ± 41.89
108	Xu et al, 2015	China	PDR: 52.12 ± 1.47<br>Control: 52.14 ± 2.06	34	973.2 ± 115.4	37	504.6 ± 59.66
109	Yoshida et al, 2015	Japan	PDR: 56.8 ± 10.5<br>Control: NA	36	23.59 ± 3.05	57	2.71±1.43
110	Yoshimura et al, 2009*	Japan	PDR: 55.8 ± 12.5<br>Control: 66.4 ± 7.8	147	2330.05 ± 2150	83	65.25 ± 44.2

No.	Study	Country	Age[a]	Case		Control	
				n	IL-6 (pg/mL, mean ± SD)	n	IL-6 (pg/mL, mean ± SD)
111	Zeng et al, 2019* (a)	China	PDR+VH: 56.28 ± 8.73<br>Control: 58.40 ± 2.03	20	19.9 ± 16.36	21	8.2 ± 7.36
112	Zeng et al, 2019* (b)	China	PDR+TRD: 55.63 ± 7.64<br>Control: 58.40 ± 2.03	19	39.05 ± 57.48	21	8.2 ± 7.36
113	Zhou et al, 2012*	China	PDR: 62.79 ± 8.97<br>Control: 60.12 ± 9.58	62	453.36 ± 284.93	20	13.07 ±12.82
**1b. Non-proliferative diabetic retinopathy (NPDR)**							
114	Feng et al, 2018 (e)	China	5 yr NPDR: NA<br>Control: 58.4 ± 5.3	10	81.838 ± 2.33	20	18.221 ± 1.45
115	Feng et al, 2018 (f)	China	10 yr NPDR: NA<br>Control: 58.6 ± 7.5	10	27.190 ± 0.37	20	16.080 ± 0.79
116	Mastropasqua et al, 2018	Italy	NPDR+DMO: 63.4 ± 7.3<br>Control: NA	20	9.65 ± 5.67	20	3.39 ± 1.27
117	Song et al, 2014 (b)	Korea	NPDR: 49.2 ± 5.8<br>Control: 50.3 ± 4.6	12	14.66 ± 19.84	12	68.89 ± 207.00
118	Wu et al, 2017 (b)	China	NPDR: 66<br>Control: 72	15	17.33 ± 9.72	10	7.10 ± 4.19
**Macular oedema (MO)**<br>**1. Diabetic macular oedema (DMO)**							
119	Bandyopadhyay et al, 2018* (a)	India	DRT: 59.29 ± 4.70<br>Control: 60.56 ± 6.81	17	34.48 ±8.73	16	20.68 ± 4.83
120	Bandyopadhyay et al, 2018* (b)	India	CMO: 61.2 ± 6.46<br>Control: 60.56 ± 6.81	20	34.35 ± 8.43	16	20.68 ± 4.83
121	Bandyopadhyay et al, 2018* (c)	India	SRD: 62.6 ± 6.19<br>Control: 60.56 ± 6.81	15	56.2 ± 15.0	16	20.68 ± 4.83
122	Bayomy and Elgouhary, 2018* (a)	Egypt	Focal DMO: 53.2 ± 2.6<br>Control: 52.5 ± 3.2	16	33.18 ± 8.3	17	18.4 ± 4.45
123	Bayomy and Elgouhary, 2018* (b)	Egypt	Diffuse DMO: 51.5 ± 3.3<br>Control: 52.5 ± 3.2	12	47.28 ± 11.58	17	18.4 ± 4.45
124	Chen et al, 2016*	China	DMO: NA<br>Control: NA	58	44.33 ± 12.95	47	42 ± 11.45
125	Dong et al, 2015* (a)	China	DMO: 67.6 ± 8.06<br>Control: 64.8 ± 6.33	34	104.75 ± 90.5	82	66.4 ± 56.5
126	Dong et al, 2015* (b)	China	DMO: 68.92 ± 8.12<br>Control: 65.71 ± 7.35	56	94.65 ± 87.5	68	65.75 ± 55.75
127	Funatsu et al, 2002 (a)*	Japan	Focal DMO/Control: 62.5 ± 9.8	18	113.15 ± 79.50	28	42.79 ± 30.2
128	Funatsu et al, 2002 (b)*	Japan	Diffuse MO+CMO/Control: 62.5 ± 9.8	8	132.6 ± 70	28	42.79 ± 30.2
129	Funatsu et al, 2003*	Japan	DMO: 64.2 ± 16.8<br>Control: 62.8 ± 13.6	26	290.65 ± 187.6	12	11.36 ± 4.60
130	Funatsu et al, 2009*	Japan	DMO: 61.2 ± 7.2<br>Control: 61.7 ± 6.5	53	306.55 ± 201.35	15	11.17 ± 4.8
131	Funk et al, 2010	Austria	DMO: 67.9 ± 10.1<br>Control: 69.9 ± 3.5	10	46.47 ± 52.6	10	15.4 ± 30
132	Jonas et al, 2012	Germany	DMO: 70.9 ± 9.3<br>Control: 74.8 ± 10.8	23	77.6 ± 136	22	7.48 ± 14.8
133	Kim et al, 2014 (a)	Korea	DRT: 61.8 ± 6.9<br>Control: 61.3 ± 6.4	27	69.22 ± 51.22	10	2.08 ± 1.67

No.	Study, year	Country	Age[a]	Case		Control	
				n	IL-6 (pg/mL, mean ± SD)	n	IL-6 (pg/mL, mean ± SD)
134	Kim et al, 2014 (b)	Korea	CMO: 60.1 ± 11.1<br>Control: 61.3 ± 6.4	18	114.31 ± 31.42	10	2.08 ± 1.67
135	Kim et al, 2014 (c)	Korea	SRD: 58.1 ± 13.3<br>Control: 61.3 ± 6.4	15	36.01 ± 26.19	10	2.08 ± 1.67
136	Kim et al, 2014 (d)	Korea	Combined: 57.3 ± 13.1<br>Control: 61.3 ± 6.4	16	98.09 ± 34.66	10	2.08 ± 1.67
137	Kim et al, 2015* (a)	Korea	DRT: 66.5 ± 10.3<br>Control: 66.6 ± 8.0	19	38.97 ± 10.72	12	16.35 ± 2.5
138	Kim et al, 2015* (b)	Korea	CMO: 62.4 ± 7.9<br>Control: 66.6 ± 8.0	17	24.32 ± 7.77	12	16.35 ± 2.5
139	Kim et al, 2015* (c)	Korea	SRD: 65.0 ± 10.2<br>Control: 66.6 ± 8.0	14	51.57 ± 7.47	12	16.35 ± 2.5
140	Lee et al, 2012	Korea	DMO: 54.8 ± 12.3<br>Control: 66.9 ± 7.3	18	66.8 ± 70.4	16	28.8 ± 39.2
141	Noma et al, 2017*	Japan	DMO: 64.3 ± 10.3<br>Control: 66.7 ± 4.7	37	14.28 ± 6.27	13	2.87 ± 1.90
142	Roh et al, 2009 (a)	Korea	CSMO1: 58.7 ± 6.6<br>Control: 63.6 ± 10.1	9	167.3 ± 292.4	11	26.4 ± 36.5
143	Roh et al, 2009 (b)	Korea	CSMO2: 66.5 ±7.5<br>Control: 63.6 ± 10.1	8	94.9 ± 70.9	11	26.4 ± 36.5
144	Sohn et al, 2011* (a)	Korea	DMO: 54.45 ± 10.22<br>Control: 63.33 ± 14.02	11	10.1 ± 38.1	6	3.5 ± 20.28
145	Sohn et al, 2011* (b)	Korea	DMO: 54.45 ± 10.22<br>Control: 63.33 ± 14.02	11	13.8 ± 43.55	6	3.5 ± 20.28
146	Sonoda et al, 2013	Japan	DMO: 62.6 ± 8.4<br>Control: 63.5 ± 7.3	15	131.7 ± 71.6	12	84.9 ± 88.5
147	Srividya et al, 2018 (a)	India	DMO: NA<br>Control: NA	11	12.08 ± 3.33	6	4.52 ± 1.37
148	Wen et al, 2015	China	DMO: 57.9 ± 11.76<br>Control: 58.9 ± 4.66	11	1.65 ± 0.10	15	1.17 ± 0.17
149	Yoshida et al, 2015 (a)	Japan	DMO: NA<br>Control: NA	12	28.24 ± 3.48	24	33.52 ± 4.11
150	Yoshimura et al, 2009* (a)	Japan	DMO: 63.0 ± 8.6<br>Control: 66.4 ± 7.8	92	382.5 ± 280.5	83	65.25 ± 44.2
151	Yu et al, 2018 (a)*	Korea	DMO: 54.45 ± 10.22<br>Control: 63.33 ± 14.02	12	46.05 ± 23.3	6	34.79 ± 23.91
152	Yu et al, 2018 (b)*	Korea	DMO: 54.45 ± 10.22<br>Control: 63.33 ± 14.02	12	31.18 ± 16.93	6	34.79 ± 23.91
**2. Non-diabetic macular oedema (Non-DMO) or MO not associated with etinal vascular occlusion (RVO)**							
153	Chu et al, 2013*	China	Non-DMO: 73.6 ± 7.43<br>Control: 70.8 ± 8.47	23	19 ± 10.5	23	13 ± 6.5
154	van Kooij et al, 2006* (a)	Netherland	CMO: 57<br>Control: 43	57	3642 ± 3438	11	87 ± 52
**Retinal vascular occlusion**<br>**1. Retinal vein occlusion (RVO)[b]**							
155	Feng et al, 2013 (a)	China	RVO: 55.28 ± 18.56/57.73 ± 11.35<br>Control: 74.00 ± 12.17	28	0.96 ± 0.23	10	0.41 ± 0.08

No.	Study, year	Country	Age[a]	Case		Control	
				n	IL-6 (pg/mL, mean ± SD)	n	IL-6 (pg/mL, mean ± SD)
156	Jung et al, 2014	Korea	RVO: 58.6 ± 23.0<br>Control: 59.2 ± 6.3	41	12.4 ± 25.2	25	0.5 ± 1.0
157	Takahashi et al, 2016 (a)	Japan	RVO: NA<br>Control: NA	10	2116.5 ± 5464.0	14	77.3 ± 278.0
**1a. Central retinal vein occlusion (CRVO)**							
158	Chen et al, 1999 (a)	Taiwan	CRVO: 63.7 ± 2.7<br>Control: 70.9 ± 3.3	5	15.6 ± 0.9	6	26.4 ± 21.8
159	Chen et al, 1999 (b)	Taiwan	CRVO+NVI: 67.8 ± 5.4<br>Control: 70.9 ± 3.3	10	1532 ± 221.1	6	26.4 ± 21.8
160	Chen et al, 1999 (c)	Taiwan	CRVO+regressed NVI: 67.8 ± 5.4<br>Control: 70.9 ± 3.3	9	234.3 ± 154.6	6	26.4 ± 21.8
161	Ehlken et al, 2015	Germany	CRVO: 74.6 ± 9.7<br>Control: 69.5 ± 9.8	13	43 ± 38	13	4 ± 3
162	Feng et al, 2013 (b)	China	CRVO: 55.28 ± 18.56<br>Control: 74.00 ± 12.17	18	0.96 ± 0.24	10	0.41 ± 0.08
163	Funk et al, 2009* (a)	Austria	CRVO: 58.2 ± 11.5<br>Control: 66.2 ± 5.3	8	83.2 ± 66.2	13	2.1 ± 2.7
164	Koss et al, 2012 (a)	Germany	CRVO: 62.9 ± 5.6<br>Control: 63.9 ± 5.82	35	64.7 ± 115.8	14	6.2 ± 3.4
165	Koss et al, 2012 (b)	Germany	H-CRVO: 62.9 ± 5.6<br>Control: 63.9 ± 5.82	16	59.9 ± 97.5	14	6.2 ± 3.4
166	Koss et al, 2013	Germany	CRVO: 71.1 ± 11.7<br>Control: 66.2 ± 7.9	35	64.7 ± 115.8	28	6.2 ± 3.4
167	Noma et al, 2009*	Japan	CRVO+MO: 69.7 ± 9.4<br>Control: 65.1 ± 6.5	27	63.68 ± 25.43	16	1.12 ± 0.20
168	Noma et al, 2010*	Japan	CRVO+MO: 70.3 ± 9.7<br>Control: 68.0 ± 6.2	16	65.79 ± 41.17	8	2.32 ± 0.76
169	Noma et al, 2012* (a)	Japan	CRVO+SRD: 69 ± 9<br>Control: 68.0 ± 6.2	10	93.55 ± 33.05	8	2.48 ± 0.41
170	Noma et al, 2012* (b)	Japan	CRVO+CMO: 70 ± 9<br>Control: 68.0 ± 6.2	10	9.26 ± 3.34	8	2.48 ± 0.41
171	Noma et al, 2013* (a)	Japan	I-CRVO+MO: 71.2 ± 6.9<br>Control: 68.1 ± 4.9	15	57.35 ± 20.65	17	0.92 ± 0.36
172	Noma et al, 2013* (b)	Japan	NI-CRVO+MO: 71.2 ± 6.9<br>Control: 68.1 ± 4.9	6	18.45 ± 8.70	17	0.92 ± 0.36
173	Noma et al, 2015*	Japan	CRVO+MO: 72.2 ± 10.7<br>Control: 67.9 ± 3.2	38	15.8 ± 5.4	15	1.59 ± 0.80
174	Rezar-Dreindl et al, 2016* (a)	Austria	CRVO: 70.4 ± 9.8<br>Control: NA	15	14.25 ± 12.45	15	72.95 ± 72.95
175	Shchuko et al, 2015 (a)	Russia	CRVO: 60.7 ± 7.5<br>Control: 60 ± 6.1	18	285.73 ± 232.51	20	52.19 ± 34.55
176	Suzuki et al, 2011	Japan	CRVO: 67.2 ± 15.1<br>Control: 70.2 ± 7.3	23	3427.40 ± 5429.39	10	188.24 ± 167.78
177	Wen et al, 2015 (a)	China	CRVO+MO: 57.91 ± 14.8<br>Control: 58.9 ± 4.66	20	1.86 ± 0.12	15	1.17 ± 0.17

No.	Study, year	Country	Age[a]	Case		Control	
				n	IL-6 (pg/mL, mean ± SD)	n	IL-6 (pg/mL, mean ± SD)
178	Yoshimura et al, 2009* (b)	Japan	CRVO: 71.6± 10.8<br>Control: 66.4 ± 7.8	13	3275 ± 2768.25	83	65.25 ± 44.2
**1b. Branch retinal vein occlusion (BRVO)**							
179	Feng et al, 2013 (c)	China	BRVO: 57.73 ± 11.35<br>Control: 74.00 ± 12.17	11	0.96 ± 0.20	10	0.41 ± 0.08
180	Funk et al, 2009* (b)	Austria	BRVO: 62.5 ± 7.9<br>Control: 66.2 ± 5.3	5	20.5 ± 21.9	13	2.1 ± 2.7
181	Kaneda et al, 2011	Japan	BRVO: 67.1 ± 1.8<br>Control: 71.4 ± 1.8	38	18.6 ± 2.6	28	10.3 ± 1.4
182	Koss et al, 2012 (c)	Germany	BRVO: 62.9 ± 5.6<br>Control: 63.9 ± 5.82	43	23.2 ± 48.8	14	6.2 ± 3.4
183	Lee et al, 2012 (a)	Korea	BRVO+MO: 61.3 ± 11.3<br>Control: 66.9 ± 7.3	12	17.7 ± 26.9	16	28.8 ± 39.2
184	Lim, 2011	Korea	BRVO: 66.5 ± 11.1<br>Control: 67.7 ± 6.7	46	7.5 ± 5.6	10	2.0 ± 1.3
185	Noma et al, 2005*	Japan	BRVO+MO: 64.5 ± 9.9<br>Control: 67.7 ± 6.7	19	9.7 ± 5.85	7	2.4 ± 0.13
186	Noma et al, 2006*	Japan	BRVO+MO: 64.3 ± 10.4<br>Control: 64.4 ± 6.7	25	69.29 ± 47.76	14	1.64 ± 0.85
187	Noma et al, 2013* (c)	Japan	BRVO+MO: 69.2 ± 9.6<br>Control: 68.8 ± 8.4	39	13.98 ± 5.87	21	0.92 ± 0.17
188	Noma et al, 2014* (a)	Japan	BRVO+MO: 71.2 ± 7.8<br>Control: 68.9 ± 6.0	28	11.98 ± 4.27	17	0.96 ± 0.14
189	Noma et al, 2014* (b)	Japan	BRVO+MO: 67.4 ± 11.0<br>Control: 65.0 ± 4.9	41	8.82 ± 1.88	9	3.53 ± 1.95
190	Noma et al, 2016*	Japan	BRVO: 69.6 ± 11.9/ 64.1 ± 7.4<br>Control: NA	38	4.33 ± 1.92	12	1.0 ± 0.47
191	Park and Ahn, 2009	Korea	BRVO: 57.2 ± 7.9<br>Control: 62.6 ± 9.5	18	38 ± 31	10	2.3 ± 1.6
192	Pfister et al, 2013	Germany	BRVO: 69.7 ± 12.9<br>Control: 66.2 ± 7.9	43	23.3 ± 48.8	28	6.2 ± 3.4
193	Rezar-Dreindl et al, 2016* (b)	Austria	BRVO: 67.9 ± 12.1<br>Control: NA	25	13.4 ± 13.4	15	72.95 ± 72.95
194	Shchuko et al, 2015 (b)	Russia	BRVO: 60.7 ± 7.5<br>Control: 60 ± 6.1	26	65.82 ± 97.46	20	52.19 ± 34.55
195	Shimura et al, 2008	Japan	BRVO+MO: 62.88 ± 5.59<br>Control: 63.9 ± 5.82	60	108.6 ± 79.5	12	11.8 ± 2.85
196	Sohn et al, 2014* (a)	Korea	BRVO+MO (1): 59 ± 9<br>Control: 67.75 ± 8.25	12	23.97 ± 11.65	6	4.23 ± 0.56
197	Sohn et al, 2014* (b)	Korea	BRVO+MO (2): 61.5 ± 8.5<br>Control: 67.75 ± 8.25	12	15.80 ± 9.62	6	4.23 ± 0.56
198	Yoshimura et al, 2009* (c)	Japan	BRVO: 69.7 ± 10.7<br>Control: 66.4 ± 7.8	30	121.8 ± 74	83	65.25 ± 44.2

No.	Study, year	Country	Age[a]	Case		Control	
				n	IL-6 (pg/mL, mean ± SD)	n	IL-6 (pg/mL, mean ± SD)
**2. Retinal artery occlusion (RAO)**							
199	Kramer et al, 2005	Israel	RAO: 67.1 ± 1.8<br>Control: 71.4 ± 1.8	8	2.6 ± 4.3	7	28.7 ± 36.0
**Age-related macular degeneration (AMD)**<br>**1. AMD[a]**							
200	Agawa et al, 2014	Japan	AMD: 71.2 ± 8.1<br>Control: 67.8 ± 12.2	37	8.4 ± 15.5	28	174.2 ± 225.9
201	Chalam et al, 2014	USA	AMD: 73.7 ± 8.6<br>Conrol: 72.8 ± 7.0	30	17.1 ± 21.3	30	5.1 ± 2.2
202	Feng et al, 2018	China	AMD: 68.78 ± 8.23<br>Control: 76.12 ± 5.30	18	49.51 ± 119.18	8	70.71 ± 91.50
203	Mimura et al, 2019*	Japan	AMD: 75.7±5.4<br>Control: 77.1±8.1	13	50.98 ± 24.03	14	17.48 ± 6.33
204	Motohashi et al, 2017	Japan	AMD: 76.3 ± 5.5<br>Control: 73.7 ± 4.9	30	17.4 ± 47.7	10	1.42 ± 2.14
205	Ten Berge et al, 2019* (a)	Netherland	AMD: 82.5 ± 6.5<br>Control: 57.25 ± 16.5	12	33.25 ± 25.75	22	39 ± 37.5
**2. Dry AMD**							
206	Kramer et al, 2012 (a)	Isarel	Moderate AMD: 79 ± 8<br>Control: 75 ± 8	16	637 ± 1010	18	351 ± 516
207	Spindler et al, 2018 (a)	Switzerland	Dry AMD: 83.5 ± 6.9<br>Control: 74.7 ± 5.6	25	3.4 ± 1.8	20	4.7 ± 7.1
**3. Wet AMD**							
208	Fauser et al, 2015	Germany	Wet AMD: 81 ± 7<br>Control: 79 ± 7	17	53 ± 168	17	5 ± 12
209	Jonas et al, 2012 (a)	Germany	Wet AMD: 80.8 ± 6.4<br>Control: 77.0 ± 9.9	18	154 ± 327.25	20	6.42 ± 17.28
210	Kramer et al, 2012 (b)	Isarel	Advanced AMD: 79 ± 8<br>Control: 75 ± 8	16	886 ± 1201	18	351 ± 516
211	Miao et al, 2012	China	Wet AMD: 68.6 ± 12<br>Control: 62.4 ± 14.5	17	3.6 ± 1.8	14	4.47 ± 2.07
212	Rezar-Dreindl et al, 2016 (a)	Austria	Wet AMD (1) : 77 ± 7.1<br>Control: NA	20	3.5 ± 8.9	15	1.4 ± 1.0
213	Rezar-Dreindl et al, 2016 (b)	Austria	Wet AMD (2) : 75 ± 7.5<br>Control: NA	20	1.8 ± 5.4	15	1.4 ± 1.0
214	Sakurada et al, 2015	Japan	Wet AMD: 74.6 ± 9.6<br>Control: 73.0 ± 6.3	18	10.9 ± 13.8	20	9.9 ± 12.6
215	Sato et al, 2018	Japan	Wet AMD: 69.0 ±10.2<br>Control: 73.7 ± 9.43	21	6.51 ± 5.24	17	78.2 ± 100
216	Spindler et al, 2018 (b)	Switzerland	Treated wet AMD: 84.9 ± 5.1<br>Control: 74.7 ± 5.6	19	17.1 ± 21.3	20	4.7 ± 7.1
217	Terao et al, 2018*	Japan	Wet AMD: 73.08 ± 1.83<br>Control: 73.5 ± 4	18	7.43 ± 2.17	11	3.29 ± 0.36

No.	Study, year	Country	Age[a]	Case		Control	
				n	IL-6 (pg/mL, mean ± SD)	n	IL-6 (pg/mL, mean ± SD)
**Choroidal neovascularization (CNV)**							
218	Fan et al, 2018	China	Idiopathic CNV: 33.25 ± 8.69<br>Control: NA	32	3.49 ± 2.41	9	4.43 ± 1.77
219	Miao et al, 2012 (a)	China	Idiopathic CNV: 33.7±10.4<br>Control: 62.4 ± 14.5	7	3.1 ± 1.5	14	4.47 ± 2.07
220	Miao et al, 2012 (b)	China	CNV (Pathologic Myopia): 49.4±10<br>Control: 62.4 ± 14.5	10	4.9 ± 2.0	14	4.47 ± 2.07
221	Roh et al, 2009 (c)	Korea	Naive CNV (AMD): 66.0 ± 16.2<br>Control: 66.0 ± 7.5	19	14.5 ± 13.1	10	20.9 ± 30.5
222	Roh et al, 2009 (d)	Korea	Reccurent CNV (AMD): 71.4 ± 5.1<br>Control: 66.0 ± 7.5	14	46.9 ± 59.5	10	20.9 ± 30.5
223	Roh et al, 2009 (e)	Korea	Regressed CNV (AMD): 64.5 ± 5.9<br>Control: 66.0 ± 7.5	17	46.4 ± 70.5	10	20.9 ± 30.5
224	Roh et al, 2010	Korea	CNV (AMD): 66.0 ± 6.2<br>Control: 63.4 ± 7.0	10	73.5 ± 172.1	9	9.5 ± 14.6
**Pachychoroid spectrum diseases<br>1. Central serous chorioretinopathy (CSC)**							
225	Jung et al, 2014	Korea	CSC: 48.77 ± 7.71<br>Control: NA	39	0.32 ± 0.79	25	0.46 ± 0.59
226	Shin and Lim, 2011	Korea	CSC: 46.4 ± 8.7<br>Control: 49.8 ± 10.7	20	1.6 ± 0.5	20	2.1 ± 1.4
**2. Pachychoroid neovasculopathy (PNV)**							
227	Feng et al, 2018	China	PNV: 73.43 ± 8.01<br>Control: 76.12 ± 5.30	7	191.14 ± 315.93	8	70.71 ± 91.50
228	Terao et al, 2018*	Japan	PNV: 67.7 ± 3.95<br>Control: 73.5 ± 4	18	4.71 ± 2.72	11	3.29 ± 0.36
**3. Polypoidal choroidal vasculopathy (PCV)**							
229	Hu et al, 2016 (a)	Australia	Refractory PCV: 62.59 ± 7.92<br>Control: 73.0 ± 6.3	41	8.59 ± 12.94	44	10.02 ± 8.93
230	Hu et al, 2016 (b)	Australia	Stable PCV: 62.56 ± 9.67<br>Control: 73.0 ± 6.3	39	5.74 ± 7.77	44	10.02 ± 8.93
231	Sakurada et al, 2015	Japan	PCV: 72.6 ± 9.2<br>Control: 73.0 ± 6.3	22	13.0 ± 43.4	20	9.9 ± 12.6
**Retinal detachment (RD)**							
232	Asensio-Sánchez et al, 2015	Spain	RD: 64.8 ± 11.4<br>Control: 68.7 ± 11.7	20	122.4 ± 16	20	46 ± 23
233	Garweg et al, 2019	Switzerland	RD: 59.0 14.6/66.9 ± 12.0<br>Control: 66.7 ± 9.1	71	121.9 ± 343.5	26	9.5 ± 20.2
234	Kunikata et al, 2013	Japan	RRD: NA<br>Control: NA	19	261 ± 674	17	21 ± 23
235	Takahashi et al, 2016	Japan	RRD: 61.2 ± 9.3<br>Control: NA	28	517.0 ± 1931.5	14	77.3 ± 278.0
236	Yoshimura et al, 2009*	Japan	RRD: 61.4 ± 10.5<br>Control: 66.4 ± 7.8	63	4203.55 ± 3837.75	83	65.25 ± 44.2

No.	Study, year	Country	Age[a]	Case n	Case IL-6 (pg/mL, mean ± SD)	Control n	Control IL-6 (pg/mL, mean ± SD)
**Proliferative vitreoretinopathy (PVR)**							
237	Benerje et al, 2007*	USA	PVR: NA<br>Control: NA	8	975 ± 3545.5	8	6 ± 6.25
238	Canataroglu et al, 2005 (a)	Turkey	PVR: 47<br>Control: 41.1	10	99.96 ± 138.67	15	0.45 ± 0.79
239	Canataroglu et al, 2005 (b)	Turkey	Traumatic PVR: 26.2<br>Control: 41.1	10	236.20 ± 107.11	15	0.45 ± 0.79
240	Kauffmann et al, 1994	Netherland	PVR: 60.2 ± 3.4<br>Control: 63 ± 6.9	15	1147 ± 1832	11	40 ± 68
241	Yamamoto et al, 2003	Japan	PVR: NA<br>Control: NA	9	888.6 ± 1012.4	8	28.1 ± 17.1
**Retinopathy of prematurity (ROP)**							
242	Sato et al, 2009* (a)	Japan	Active ROP: NA<br>Control: NA	12	97.7 ± 35.2	6	1.35 ± 1.0
243	Sato et al, 2009* (b)	Japan	Inactive ROP: NA<br>Control: NA	15	188.33 ± 132.18	6	1.35 ± 1.0
**Coats' disease**							
244	Feng et al, 2017* (a)	China	Coats' disease (child): 6.45 ± 1.97<br>Control: 3.50 ± 0.96	12	28.17 ± 15.44	6	6.60 ± 2.12
245	Feng et al, 2017* (b)	China	Coats' disease (adult): 32.75 ± 9.36<br>Control: 62.59 ± 4.19	8	58.96 ± 46.57	10	7.64 ± 4.64
**Retinitis pigmentosa (RP)**							
246	Ten Berge et al, 2019*	Netherland	RP: 53.25 ± 15.25<br>Control: 57.25 ± 16.5	25	37.25 ± 34.75	22	39 ± 37.5
247	Yoshida et al, 2013	Japan	RP: 64.7 ± 12.2<br>Control: 67.4 ± 7.5	14	21.1 ± 14.0	20	3.4 ± 0.00
**Epiretinal membrane (ERM)**							
248	Takahashi et al, 2016	Japan	ERM: NA<br>Control: NA	9	112.0 ± 303.75	14	77.3 ± 278.0
249	Zandi et al, 2016	Switzerland	ERM: 73 ± 8.5<br>Control: 71 ± 8.2	31	5.33 ± 3.30	30	0.80 ± 1.55
**Others**							
250	Jakobsson et al, 2015	Sweden	Pseudophakia: 71.9 ± 8.43<br>Control: 68.3 ± 7.31	40	18.9 ± 20.5	33	18.4 ± 19.0
251	Sauer et al., 2016	France	Congentital cataract: 3.8 months<br>Control: 72	18	655.1 ± 356.5	20	67.0 ± 54.2
252	Suzuki et al, 2019	Japan	PBK: 73.8 ± 10.5<br>Control: 75.9 ± 7.6	62	2127 ± 495	33	85 ± 69
253	Zhu et al, 2016 (a)[c]	China	HMC: 61.83 ± 8.69<br>Control: 63.35 ± 9.26	45	6.23 ± 1.50	35	19.78 ± 22.75
254	Zhu et al, 2016 (b)[c]	China	HMC: 61.83 ± 8.69<br>Control: 63.35 ± 9.26	15	6.77 ± 4.01	15	0.00 ± 0.00

[a]Age was expressed as mean or mean ± SD; [b]Did not stratify based on clinical classification or etiology; [c]quantified with both methods. * Indicate the mean and SD were estimated from median, range, and the size of the sample as previously described[1,2]. BSCR, birdshot chorioretinopathy/birdshot uveitis/HLA-A29 uveitis; CMO, cystoid macular oedema; CMV, cytomegalovirus; CSMO, clinically significant macular oedema; DRT, diffuse retinal thickening; DVR, diabetic vitreoretinopathy; ED, Eales disease; FHC, fuchs' heterochromic cyclitis; H-CRVO, hemi-central retinal vein occlusion; HLA, human leukocyte antigen; HMC, high myopia cataract; I-CRVO, ischemic-central retinal vein occlusion; IU, Idiopathic uveitis; LIU, lens-induced uveitis; NA, not available; NI-CRVO, non-ischemic-central retinal vein occlusion; NVG, neovascular glaucoma; NVI, iris neovascularization; PBK, pseudophakic bullous keratopathy; PEXG, pseudoexfoliative glaucoma; PRP, pan-retinal photocoagulation; PSS, posner-schlossman

syndrome; RRD, rhegmatogenous retinal detachment; SRD, serous retinal detachment; TAU, tuberculosis associated uveitis, TRD, tractional retinal detachment, TU, toxoplasma uveitis; UG, uveitic glaucoma; VB, vitreous bleeding; VH, vitreous hemorrhage.

Supplemental Table S4. Meta-analysis for the association between intraocular IL-6 levels and ocular disease stratified by the type of disease

No.	Group	No. of study	Subjects	Method[a]	Effect estimate SMD [95% CI]	Heterogeneity P	Heterogeneity I^2 (%)	Assoc. (P)	P Egger's test
1	Overall	254	11014	Random	1.41 [1.24, 1.58]	<0.00001	92	<0.00001	0.000
2	Glaucoma	30	1606	Random	0.80 [0.26, 1.35]	<0.00001	95	0.004	0.169
2a	POAG	15	775	Random	0.15 [-0.48, 0.78]	<0.00001	94	0.64	0.228
2b	PACG	4	168	Random	0.52 [-0.09, 1.14]	0.01	72	0.09	0.066
2c	Secondary glaucoma	11	663	Random	1.84 [0.59, 3.09]	<0.00001	97	0.004	0.164
3	PEX syndrome	7	309	Random	1.56 [0.19, 2.93]	<0.00001	96	0.03	0.051
4	Ocular inflammation	33	892	Random	1.75 [1.25, 2.25]	<0.00001	87	<0.00001	0.004
4a	Surface ocular inflammation	2	62	Random	4.43 [-1.66, 10.52]	<0.00001	94	0.15	NA
4b	Intraocular inflamation	31	899	Random	1.62 [1.15, 2.09]	<0.00001	86	<0.00001	0.003
4b1	Uveitis*	7	179	Random	0.67 [-0.45, 1.79]	<0.00001	87	0.24	0.063
4b2	Infectious	5	213	Random	2.22 [0.94, 3.49]	<0.00001	90	0.0007	0.595
4b3	Inflammatory	12	301	Random	2.03 [1.35, 2.72]	<0.00001	79	<0.00001	0.002
4b4	Pseudo-uveitis	3	110	Random	1.81 [0.17, 3.45]	<0.0001	90	0.03	0.415
4b5	Ophthalmologic entities	4	96	Random	1.05 [0.59, 1.51]	0.62	0	<0.00001	0.384
5	Diabetic retinopathy	48	2568	Random	2.21 [1.74, 2.69]	<0.00001	95	<0.00001	0.000
5a	DR*	13	901	Random	1.97 [1.17, 2.76]	<0.00001	95	<0.00001	0.001
5b	PDR	30	1518	Random	2.17 [1.55, 2.78]	<0.00001	95	<0.00001	0.000
5c	NPDR	5	149	Random	5.55 [2.67, 8.42]	<0.00001	96	0.0002	0.025
6	Macular oedema	36	1510	Random	1.48 [1.14, 1.82]	<0.00001	86	<0.00001	0.000
6a	DMO	34	196	Random	1.52 [1.16, 1.89]	<0.00001	87	<0.00001	0.000
6b	Non-DMO	2	114	Random	0.87 [0.42, 1.31]	0.0001	0	0.34	NA
7	Retinal vascular occlusion	45	1782	Random	1.68 [1.29, 2.07]	<0.00001	90	<0.00001	0.000
7a	RVO	3	128	Random	1.22 [0.03, 2.41]	0.0006	87	0.04	0.453
7b	CRVO	21	723	Random	1.99 [1.37, 2.61]	<0.00001	90	<0.00001	0.008
7c	BRVO	20	916	Random	1.58 [1.00, 2.15]	<0.00001	90	<0.00001	0.006
8	AMD	18	682	Random	0.29 [-0.07, 0.65]	<0.00001	80	0.11	0.021
8a	AMD*	6	252	Random	0.33 [-0.47, 1.14]	<0.00001	88	0.42	0.285
8b	Dry AMD	2	79	Random	0.02 [-0.58, 0.62]	<0.00001	44	0.94	NA
8c	Wet AMD	10	351	Random	0.33 [-0.13, 0.79]	<0.00001	77	0.16	0.110
9	CNV	7	209	Random	0.07 [-0.29, 0.43]	0.16	35	0.72	0.729
10	Pachychoroid spectrum diseases	7	358	Random	-0.11 [-0.39, 0.16]	0.14	38	0.42	0.99
11	RD	5	361	Random	1.23 [0.31, 2.15]	<0.00001	92	0.008	0.144
12	PVR	5	109	Random	1.27 [0.42, 2.11]	0.005	73	0.003	0.063
13	ROP	2	32	Random	2.27 [0.68, 3.86]	0.10	62	0.005	NA
14	Coats' disease	2	36	Random	1.59 [0.79, 2.38]	0.98	0	<0.0001	NA
15	RP	2	81	Random	0.92 [-1.02, 2.86]	0.0001	93	0.35	NA
16	ERM	2	84	Random	0.95 [-0.63, 2.53]	0.002	89	0.24	NA
17	Others	5	326	Random	1.77 [-0.17, 3.72]	<0.00001	98	0.07	0.052

Assoc., test of association. *Did not stratify based on clinical classification or etiology. AMD, age-related macular degeneration; BRVO, branch retinal vein occlusion; CNV, choroidal neovascularization; CRVO, central retinal vein occlusion; DMO, dibetic macular oedema; DR, diabetic retinopathy; ERM, epiretinal membrane; NPDR, non-proliferative diabetic retinopathy; PACG, primary angle-closure glaucoma; PDR, proliferative diabetic retinopathy; PEX, pseudoexfoliation syndrome; POAG, primary open-angle glaucoma; PVR, proliferative vitreoretinopathy; RD, retinal detachment; ROP, retinopathy of prematurity; RP, retinitis pigmentosa; RVO, retinal vein occlusion.

Supplemental Table S5. Meta-analysis for the association between intraocular IL-6 levels and ocular disease stratified by ethinicity, type of sample, and quantification method.

Contrast	Group	No. of study	Method	Effect estimate SMD [95% CI]	Heterogeneity P	Heterogeneity I^2 (%)	Assoc. (P)
Ethnicity	Asian	164	Random	1.72 [1.49, 1.95]	0.000	94	<0.001
	Caucasian	85	Random	0.94 [0.70, 1.18]	0.000	89	<0.001
	Middle eastern	5	Random	0.03 [-2.59, 2.65]	0.000	98	0.981
Type of sample	Aqueous humor	180	Random	1.30 [1.10, 1.51]	0.000	92	<0.001
	Vitreous humor	74	Random	1.68 [1.38, 1.97]	0.000	92	<0.001
Quantification method	ELISA	80	Random	2.48 [2.03, 2.94]	0.000	95	<0.001
	Multiplex bead immunoassay or immunoarray	174	Random	1.04 [0.87, 1.21]	0.000	90	<0.001

Assoc., test of association. ELISA, enzyme-linked immunosorbent assay.

Supplemental Table S6. Meta-regression analysis with the aid of year of publication, type of disease, type of sample, quantification method, ethnicity, and sample size on IL-6 levels in ocular disease

Variable	Method	Coefficients	95% CI	P-value
Year of publication	Random	-0.004	[-0.053, 0.046]	0.883
Type of disease	Random	0.810	[0.092, 1.527]	0.027
Type of sample	Random	0.275	[-0.305, 0.854]	0.353
Quantification method	Random	1.388	[0.826, 1.951]	<0.001
Ethnicity	Random	1.019	[0.573, 1.464]	0.004
Sample size	Random	0.003	[-0.006, 0.012]	0.515

- Type of disease: 1-Glaucoma; 2-PEX (pseudoexfoliation) syndrome; 3-Ocular inflammation; 4-Diabetic retinopathy; 5-Macular oedema; 6-Vascular occlusion; 7-AMD (age-related macular degeneration); 8-CNV (choroidal neovascularization); 9- Pachychoroid spectrum diseases; 10-RD (retinal detachment); 11-PVR (proliferative vitreoretinopathy); 12-ROP (retinopathy of prematurity); 13- Coats' disease; 14-RP (retinitis pogmentosa); 15-ERM (epiretinal membrane); 16-Others.
- Type of sample: 1-Aqueous humor; 2-Vitreous humor.
- Quantification method: 1-ELISA (enzyme-linked immunosorbent assay) ; 2-Multiplex bead immunoassay or immunoarray.
- Ethnicity: 1-Asian; 2-Caucasian; 3-Middle Eastern.

Supplemental Table S7. Network meta-analysis for DMO pattern based on optical coherence tomography (OCT)

**DRT**			
0.17 [-1.22 – 1.55]	**CMO**		
-0.75 [-2.33 – 0.82]	-0.89 [-4.31 – 2.54]	**SRD**	
3.74 [1.30 – 6.18]*	2.36 [0.92 – 3.80]*	1.97 [1.43 – 2.51]*	**Control**

*P < 0.05. CMO, cystoid macular oedema; DRT, diffuse retinal thickening; SRD, serous retinal detachment.

Meta-analysis to estimate IL-6 levels in tears of patient with surface ocular diseases

A literature search was conducted from PubMed, Scopus and Web of Science databases up to December 2019 using the following keywords and their combination such as "IL-6 or interleukin-6", "cytokine", "inflammation", and "surface eye disease/ophthalmology". For study selection, data extraction and statistical analysis see the method section of this article. A total of 140 relevant studies were initially screened. After reviewing the titles, abstract, duplications and availability full-text of abstract, 29 articles were excluded. One hundred ten studies were further evaluated, 80 were excluded (unable to extract the data (n=21), not a case-control study (n=29), not related with surface ocular disease (n=14), review and animal model (n=4), other methods, measuring other than IL-6, collecting sample other than tears (n=9). Therefore, 34 studies were included in this meta-analysis (**Supplemental Table S8**). IL-6 levels in tears of patients with surface ocular disease displayed a significant increase compared to the control subjects (SMD = 2.07, 95% CI = 1.67 – 2.48, P < 0.00001, **Supplemental Fig. S9**). Although publication bias existed (PEgger's test < 0.05) and the trim and fill method was used, the results remain identical.

Supplemental Table S8. Summary of the studies for the IL-6 levels in tears between patients with surface ocular disease and the control subjects

No.	Study, year	Country	Age[a]	Case		Control	
				n	IL-6 (pg/mL, mean ± SD)	n	IL-6 (pg/mL, mean ± SD)
1	Acere et al, 2008	Chile	CCh: 62 ± 7.1<br>Control: 40 ± 12.21	25	51.78 ± 81.53	18	8.15 ± 2.73
			Blepharitis: 44 ± 9.8<br>Control: 40 ± 12.21	13	16.53 ± 1.26	18	8.15 ± 2.73
			Hyposecretive DED: 56 ± 12.63<br>Control: 40 ± 12.21	20	16.52 ± 10.58	18	8.15 ± 2.73
			Allergic eye disease: 43 ± 12.7<br>Control: 40 ± 12.21	19	33.11 ± 8.66	18	8.15 ± 2.73
2	Benitez-del-Castillo et al, 2018	Spain	DED: 52 ± 15<br>Control: 50 ± 12	30	18.0 ± 6.4	36	12.1 ± 8.6
3	Chao et al, 2018*	USA	Asymptomatic CL/Control: between 18-40	9	17.3 ± 1.025	9	16.1 ± 1.025
4	Yüksel Elgin et al, 2015	Turkey	Asymptomatic CL (a): 25.67 ± 7.75<br>Control: 25.75 ± 8.24	9	2643.7 ± 259.49	9	2634.49 ± 251.99
			Asymptomatic CL (b): 24.78 ± 5.95<br>Control: 25.75 ± 8.24	9	2387.43 ± 58.74	9	2634.49 ± 251.99
5	Erdogan-Poyraz et al, 2009	Turkey	CCh1-3: 63.4 ± 6.9<br>Control: 63.1 ± 6.3	16<br>21<br>14	14.9 ± 2.3<br>32.4 ± 14.0<br>184.4 ± 155.4	10<br>10<br>10	13.9 ± 1.1<br>13.9 ± 1.1<br>13.9 ± 1.1
6	Fodor et al, 2006	Hungary	Bacterial conjunctivitis	11	366 ± 296	52	110 ± 142
			Corneal foreign body	7	109 ± 72	52	110 ± 142
7	Gonzalez-Perez et al, 2012	Spain	Asymptomatic CL (a): 25.4 ±65.04<br>Control: 26.72 ± 5.12	28	2.6 ± 0.6	32	2.2 ± 0.5
			Asymptomatic CL (b): 27.00 ± 7.38<br>Control: 26.72 ± 5.12	32	4.7 ± 1.2	32	2.2 ± 0.5
8	Jackson et al, 2016	Australia	DED: 43.4 ± 2.0<br>Control: 40.6 ± 3.6	62	52.8 ± 18.1	15	45.1 ± 6.2
9	Jun et al, 2011	USA	VKC: 43<br>Control: 33	18	282.7 ± 467.7	11	89.5 ± 83.5
10	Jung et al, 2016	Korea	MGD: 69.8 ± 10.4<br>Control: 65.8 ± 9.93	15	87.53 ± 41.46	35	47.94 ± 31.07

No.	Study, year	Country	Age[a]	Case		Control	
				n	IL-6 (pg/mL, mean ± SD)	n	IL-6 (pg/mL, mean ± SD)
11	Lam et al, 2009	USA	DTS: 55.2 ± 16.4<br>Control: 45 ± 17.3	30	238.0 ± 278.2	14	26.5 ± 21.8
12	Landsend et al, 2018	Norway	Aniridia+MGD: 34.9 ± 18.7<br>Control: range 31.2 ± 13.9	35	6.5 ± 6.6	21	1.7 ± 1.8
13	Lee et al, 2013	Korea	(SS) DED: 55.9 ± 9.96<br>Control: 52.8 ± 13.19	40	19220 ± 20110	35	6970 ± 6730
			(Non-SS) DED: 55.4 ± 12.44<br>Control: 52.8 ± 13.19	40	12120 ± 13540	35	6970 ± 6730
14	Lema and Durán, 2005*	Spain	KC: 22.4 ± 6.5<br>Control: 22.6 ± 6.6	28	29.0 ± 1.5	20	9.5 ± 0.775
15	Lema et al, 2009*	Spain	KC: 27.1 ± 8.1<br>Control: 22.6 ± 6.6	30	22.8 ± 0.5	20	9.5 ± 0.775
			Subclinical KC: 27.1 ± 8.1<br>Control: 22.6 ± 6.6	30	22.1 ± 0.425	20	9.5 ± 0.775
16	Leonardi et al, 1998	Italy	VKC tarsal: 12.8 ± 1.0<br>Control: NA	10	28.0 ± 43.9	14	0.00 ± 0.00
			VKC limbal: 12.8 ± 1.0<br>Control: NA	9	58.5 ± 161.3	14	0.00 ± 0.00
17	Massingale et al, 2009	USA	DED: 52.8 ± 10.8<br>Control: 50.3 ± 9.4	7	1625.7 ± 430.9	7	632.3 ± 167.9
18	Matsumura et al, 2017	Japan	LDO: 26.9 ± 28.7 months<br>Control: NA	15	666 ± 532	15	28.0 ± 46.3
19	McDonnell et al, 2017	USA	DED: 63.2 ± 10.5<br>Control: 61.1 ± 11.2	217	147.45 ± 476.145	67	104.72 ± 290.994
20	Mrugacz et al, 2017	Poland	DED: 44.21<br>Control: 44.85	32	37790 ± 11280	34	6470 ± 2100
21	Nivenius et al, 2011*	Sweden	AKC: 46<br>Control: NA	11	778 ± 25.5	5	286 ± 10
			SAC: NA<br>Control: NA	5	613 ± 15.25	5	286 ± 10
22	Oray and Toker, 2013	Turkey	VKC/Control: 11.3 ± 4.3	21	236.2 ± 307.4	15	106.6 ± 99.4
23	Pflugfelder et al, 1999	USA	(SS) keratoconjunctivitis sicca: 64 ± 8.3<br>Control: 26 ± 3.8	10	3690 ± 3200	10	290 ± 500
24	Saeki et al., 2017	Japan	SBS: 37.3 ± 16.6<br>Control: 31.2 ± 10.2	22	15.8 ± 15.8	13	8.3 ± 8.3
			AC: 28.9 ± 14.5<br>Control: 31.2 ± 10.2	14	11.5 ± 11.1	13	8.3 ± 8.3
			AKC: 27.1 ± 13.6<br>Control: 31.2 ± 10.2	14	21.2 ± 22.8	13	8.3 ± 8.3
			VKC: 18.0 ± 5.8<br>Control: 31.2 ± 10.2	21	48.2 ± 38.3	13	8.3 ± 8.3
25	Sakimoto et al, 2014	Japan	noninfectious corneal ulcer: NA<br>Control: NA	16	1394 ± 1399	12	17.5 ± 20.3
26	Santacruz et al, 2015	Mexico	Fungal keratitis: 49 ± 19<br>Control: 52 ± 19	14	4172 ± 1873	48	25 ± 17

No.	Study, year	Country	Age[a]	Case		Control	
				n	IL-6 (pg/mL, mean ± SD)	n	IL-6 (pg/mL, mean ± SD)
	Santacruz et al, 2015		Gram-positive bacterial keratitis: 65 ± 19<br>Control: 52 ± 19	8	758 ± 1166	48	25 ± 17
			Gram-negative bacterial keratitis: 51 ± 19<br>Control: 52 ± 19	6	1596 ± 971	48	25 ± 17
27	Shetty et al, 2015	India	KC: NA<br>Control: NA	12	4050 ± 730	10	2650 ± 890
28	Shetty et al, 2017	India	KC: 28.8 ± 2<br>Control: 28.0 ± 3	7	2413 ± 489	6	49 ± 21
29	Sorkhabi et al, 2014	Iran	KC: 24.09 ± 6.50<br>Control: 24.43 ± 4.55	42	17.49 ± 1.92	30	13.81 ± 1.71
30	Tai et al, 2019*	Malaysia	ARC: 27.56 ± 14.90<br>Control: 29.75 ± 10.20	25	1018.62 ± 373	20	74.36 ± 82.06
31	Tishler et al, 1999	Israel	(SS) DED: 55.3 ± 6.3<br>Control: 60.1 ± 9.9	12	88.6 ± 16.2	12	42.1 ± 10.6
32	Ujhelyi et al, 2012*	Hungary	GO: 43.4 ± 15.2<br>Control: 38.6 ± 13.8	54	752.51 ± 35.65	24	457.9 ± 21.44
33	Van Acker et al, 2019	Belgium	Primary pterygia/control: 52 ± 4	14	27.0 ± 8.0	37	17.0 ± 2.1
			Reccurent pterygia/control: 52 ± 4	7	41.5 ± 13.5	37	17.0 ± 2.1
			Pinguecula/Control: 52 ± 4	5	30.8 ± 16.0	37	17.0 ± 2.1
34	Zhao et al, 2018	China	(SS) DED: NA<br>Control: NA	20	9.46 ± 9.16	15	3.79 ± 4.31
			(non-SS) DED: NA<br>Control: NA	20	6.78 ± 7.63	15	3.79 ± 4.31
			Evaporative DED/MGD: NA<br>Control: NA	15	9.19 ± 9.98	15	3.79 ± 4.31

[a]Age was expressed as mean or mean ± SD. *Indicate the mean and SD were estimated from median, range, and the size of the sample as previously described[1,2]. AC, allergic conjunctivitis; AKC, atopic keratoconjunctivitis; ARC, allergic rhinoconjunctivitis; CCh, conjunctivochalasis; CL, contact lens; DED, dry eye disease; DTS, dysfunctional tear syndrome; GO, graves' orbitopathy; KC, keratoconus; LDO, lacrimal duct obstruction; MGD, meibomian gland dysfunction; NA, not available; SAC, seasonal allergic conjunctivitis; SBS, sick building syndrome; SS, sjogren syndrome; TAO, thyroid-associated ophthalmopathy/orbitopathy; VKC, vernal keratoconjunctivitis.

Supplemental Table S9. Quality assessments of included case-control studies using the Newcastle Ottawa Scale[3]

A. IL-6-174 G/C polymorphism

No.	Study, year	Selection				Comparability		Exposure			Total no. of stars
		1	2	3	4	1 (a)	1 (b)	1 (a)	2	3	
1	Lin et al, 2014	*	*	*	*	*	*	*	*	*	9
2	Wang et al, 2017	*	-	-	-	*	*	*	*	*	6
3	Wu et al, 2016	*	*	*	*	*	*	*	*	*	9
4	Zimmermann et al, 2013	*	*	*	*	*	*	*	*	*	9
5	Goverdhan et al, 2008	*	*	*	*	*	*	*	*	*	9
6	Lu et al, 2016	*	*	*	*	*	*	*	*	*	9
7	Masood et al, 2007	*	*	*	*	*	*	*	*	*	9
8	Myśliwska et al, 2009	*	*	*	*	*	*	*	*	*	9
9	Paine et al, 2012	*	*	*	*	*	*	*	*	*	9
10	Sanabria Ruiz-Colmenares et al, 2006	*	*	*	*	*	*	*	*	*	9
11	Anvari et al, 2009	*	*	*	*	*	-	*	*	*	8
12	Bednarczuk et al, 2004	*	*	*	*	*	*	*	*	*	9
13	Carnt et al, 2012	*	*	*	*	*	-	*	*	*	8
14	Cordeiro et al, 2013	*	*	*	*	*	*	*	*	*	9
15	Dilek et al, 2009	*	*	*	*	*	*	*	*	*	9
16	Ewald et al, 2015	*	*	*	*	*	-	*	*	*	8
17	Gedvilaite et al, 2019	*	*	*	*	*	*	*	*	*	9
18	Na et al, 2011	*	*	*	*	*	-	*	*	*	8
19	Sen et al, 2011	*	*	*	*	*	*	*	*	*	9
20	Taalat et al, 2014	*	*	*	*	*	*	*	*	*	9
21	Steinbrugger et al, 2009	*	*	*	*	*	*	*	*	*	9
22	Weger et al, 2004	*	*	*	*	*	*	*	*	*	9
23	Kucherenko et al, 2013	*	*	*	*	*	-	*	*	*	8

"-" means no star was assigned.

B. IL-6 levels

No.	Study, year	Selection				Comparability		Exposure			Total no. of stars
		1	2	3	4	1 (a)	1 (b)	1 (a)	2	3	
1	Borkenstein et al, 2013	*	*	*	*	*	*	*	*	*	9
2	Chua et al, 2012	*	*	*	*	*	*	*	*	*	9
3	Engel et al, 2014	*	*	*	*	*	*	*	*	*	9
4	Freedman and Iserovich, 2013	*	*	*	*	-	*	*	*	*	8
5	Ghanem et al, 2011	*	*	*	*	*	*	*	*	*	9
6	Inoue-Mochita et al, 2018	*	*	*	*	*	-	*	*	*	8
7	Khalef et al, 2017	*	*	*	*	*	*	*	*	*	9
8	Kutchey et al, 2010	*	*	*	*	*	*	*	*	*	9
9	Niu, 2018	*	*	*	*	*	*	*	*	*	9
10	Ohira et al, 2015	*	*	*	*	*	-	*	*	*	8
11	Sorkhabi et al, 2010	*	*	*	*	*	*	*	*	*	9
12	Takai et al, 2012	*	*	*	*	*	-	*	*	*	8
13	Ten Berge et al, 2019	*	*	*	*	*	-	*	*	*	8
14	Zenkel et al, 2010	*	*	*	*	*	*	*	*	*	9
15	Du et al, 2016	*	*	-	-	*	-	*	*	*	6
16	Duvesh et al, 2017	*	*	*	*	*	*	*	*	*	9
17	Liu et al, 2017	*	*	*	*	*	-	*	*	*	8
18	Wang et al, 2018	*	*	*	*	*	-	*	*	*	8
19	Kovacs et al, 2015	*	*	*	*	*	*	*	*	*	9
20	Li et al, 2012	*	*	*	*	*	-	*	*	*	8
21	Liu et al, 2017	*	*	*	*	*	*	*	*	*	9
22	Rusnak et al, 2015	*	*	*	*	*	-	*	*	*	8

No.	Study, year	Selection				Comparability		Exposure			Total no. of stars
		1	2	3	4	1 (a)	1 (b)	1 (a)	2	3	
23	Sarenac V. et al, 2015	*	*	*	*	*	-	*	*	*	8
24	Garweg et al, 2017	*	*	*	*	*	-	*	*	*	8
25	Aketa at el, 2017	*	*	*	*	*	*	*	*	*	9
26	Zhang et al, 2018	*	*	*	*	*	-	*	*	*	8
27	Banerjee et al, 200	*	*	*	*	*	-	*	*	*	8
28	Hernández Garfella et al, 2015	*	*	*	*	*	-	*	*	*	8
29	Petrinović-Doresić, et al 1999	*	*	*	*	*	-	*	*	*	8
30	Sijssens et al, 20	*	*	*	*	*	*	*	*	*	9
31	van Kooij et al, 2006	*	*	*	*	*	*	*	*	*	9
32	Wakefield et al, 1995	*	*	*	*	*	-	*	*	*	8
33	Ang et al, 201	*	*	*	*	-	-	*	*	*	7
34	Curnow et al, 2005	*	*	*	*	*	-	*	*	*	8
35	Murray et al, 1990	*	*	*	*	*	-	*	*	*	8
36	Sauer et al, 2018	*	*	*	*	*	*	*	*	*	9
37	Bae and Lee, 2012	*	*	*	*	*	-	*	*	*	8
38	Chen et al, 2014	*	*	*	*	*	*	*	*	*	9
39	Murugeswari et al, 2008	*	*	*	*	*	-	*	*	*	8
40	Murugeswari et al, 2014	*	*	*	*	*	-	*	*	*	8
41	Nagata et al, 2012	*	*	*	*	*	*	*	*	*	9
42	Valentincic et al, 2011	*	*	*	*	*	*	*	*	*	9
43	Aketa et al, 2017 (a)	*	*	-	*	*	*	*	*	*	8
44	Nagarkatti-Gude et al, 2012	*	*	*	*	*	-	*	*	*	8
45	Kuiper et al, 2011	*	*	*	*	*	-	*	*	*	8
46	Perez et al, 2004	*	*	*	*	*	*	*	*	*	9
47	Boss et al, 2017	*	*	*	*	*	*	*	*	*	9
48	Chen et al, 2017	*	*	*	*	*	*	*	*	*	9
49	Chernykh et al, 2015	*	*	*	*	*	*	*	*	*	9
50	Cheung et al, 2012	*	*	*	*	*	*	*	*	*	9
51	Dong et al, 2013	*	*	*	*	*	*	*	*	*	9
52	Feng et al, 2018	*	*	*	*	*	*	*	*	*	9
53	Funatsu et al, 2001	*	*	*	*	*	-	*	*	*	8
54	Gustavsson et al, 2012	*	*	*	*	*	*	*	*	*	9
55	Oh et al, 2010	*	*	*	*	*	-	*	*	*	8
56	Sin et al, 2013	*	*	*	*	*	-	*	*	*	8
57	Suzuki et al, 2011	*	*	*	*	*	-	*	*	*	8
58	Yuuki et al, 2001	*	*	*	*	*	-	*	*	*	8
59	Abu el Asrar et al, 1992	*	*	*	*	*	-	*	*	*	8
60	Adamiec-Mroczek and Oficjalska-Młyńczak, 2008	*	*	*	*	*	*	*	*	*	9
61	Bromberg-White et al, 2013	*	*	*	*	*	-	*	*	*	8
62	Canataroglu et al, 2005	*	*	*	*	*	-	*	*	*	8
63	Kauffmann et al, 1994	*	*	-	-	*	-	*	*	*	6
64	Koskela et al, 2013	*	*	*	*	*	*	*	*	*	9
65	Loukovaara et al, 2017	*	*	*	*	*	-	*	*	*	8
66	Mocan et al, 2006	*	*	*	*	*	*	*	*	*	9
67	Nakamura et al, 2003	*	*	*	*	*	*	*	*	*	9
68	Schoenberger et al, 2012	*	*	*	*	*	-	*	*	*	8
69	Shimura et al, 2009	*	*	-	-	*	-	*	*	*	6
70	Song et al, 2014	*	*	*	*	*	*	*	*	*	9
71	Srividya et al, 2018	*	*	*	*	*	-	*	*	*	8
72	Takahashi et al, 2016	*	*	*	*	*	-	*	*	*	8
73	Tsai et al, 2018	*	*	*	*	*	*	*	*	*	9

No.	Study, year	Selection				Comparability		Exposure			Total no. of stars
		1	2	3	4	1 (a)	1 (b)	1 (a)	2	3	
74	Wu et al, 2017	*	*	*	*	*	-	*	*	*	8
75	Xu et al, 2015	*	*	*	*	*	*	*	*	*	9
76	Yoshida et al, 2015	*	*	*	*	*	-	*	*	*	8
77	Yoshimura et al, 2009	*	*	*	*	*	-	*	*	*	8
78	Zeng et al, 2019	*	*	*	*	*	*	*	*	*	9
79	Zhou et al, 2012	*	*	*	*	*	*	*	*	*	9
80	Mastropasqua et al, 2018	*	*	*	*	*	-	*	*	*	8
81	Bandyopadhyay et al, 2018	*	*	*	*	*	*	*	*	*	9
82	Bayomy and Elgouhary, 2018	*	*	*	*	*	*	*	*	*	9
83	Chen et al, 2016	*	*	*	*	*	*	*	*	*	9
84	Dong et al, 2015	*	*	*	*	*	*	*	*	*	9
85	Funatsu et al, 2002	*	*	*	*	*	-	*	*	*	8
86	Funatsu et al, 2003	*	*	*	*	*	*	*	*	*	9
87	Funatsu et al, 2009	*	*	*	*	*	*	*	*	*	9
88	Funk et al, 2010	*	*	*	*	*	*	*	*	*	9
89	Jonas et al, 2012	*	*	*	*	*	*	*	*	*	9
90	Kim et al, 2014	*	*	*	*	*	*	*	*	*	9
91	Kim et al, 2015	*	*	*	*	*	*	*	*	*	9
92	Lee et al, 2012	*	*	*	*	*	-	*	*	*	8
93	Noma et al, 2017	*	*	*	*	*	*	*	*	*	9
94	Roh et al, 2009	*	*	*	*	*	-	*	*	*	8
95	Sohn et al, 2011	*	*	*	*	*	*	*	*	*	9
96	Sonoda et al, 2013	*	*	*	*	*	-	*	*	*	8
97	Wen et al, 2015	*	*	*	*	*	*	*	*	*	9
99	Yu et al, 2018	*	*	*	*	*	*	*	*	*	9
100	Chu et al, 2013	*	*	*	*	*	*	*	*	*	9
101	Feng et al, 2013	*	*	*	*	*	-	*	*	*	8
102	Jung et al, 2014	*	*	*	*	*	*	*	*	*	9
103	Chen et al, 1999	*	*	*	*	*	*	*	*	*	9
104	Ehlken et al, 2015	*	*	*	*	*	*	*	*	*	9
105	Funk et al, 2009	*	*	*	*	*	*	*	*	*	9
106	Koss et al, 2012	*	*	*	*	*	*	*	*	*	9
107	Koss et al, 2013	*	*	*	*	*	*	*	*	*	9
108	Noma et al, 2009	*	*	*	*	*	*	*	*	*	9
109	Noma et al, 2010	*	*	*	*	*	*	*	*	*	9
110	Noma et al, 2012	*	*	*	*	*	*	*	*	*	9
111	Noma et al, 2013	*	*	*	*	*	*	*	*	*	9
112	Noma et al, 2015	*	*	*	*	*	*	*	*	*	9
113	Rezar-Dreindl et al, 2016	*	*	*	*	*	*	*	*	*	9
114	Shchuko et al, 2015	*	*	*	*	*	*	*	*	*	9
115	Kaneda et al, 2011	*	*	*	*	*	*	*	*	*	9
116	Lim, 2011	*	*	*	*	*	*	*	*	*	9
117	Noma et al, 2005	*	*	*	*	*	*	*	*	*	9
118	Noma et al, 2006	*	*	*	*	*	*	*	*	*	9
119	Noma et al, 2013 (a)	*	*	*	*	*	*	*	*	*	9
120	Noma et al, 2014	*	*	*	*	*	*	*	*	*	9
121	Noma et al, 2014 (a)	*	*	*	*	*	*	*	*	*	9
122	Noma et al, 2016	*	*	*	*	*	*	*	*	*	9
123	Park and Ahn, 2009	*	*	*	*	*	*	*	*	*	9
124	Pfister et al, 2013	*	*	*	*	*	*	*	*	*	9
125	Shimura et al, 2008	*	*	*	*	*	*	*	*	*	9
126	Sohn et al, 2014	*	*	*	*	*	*	*	*	*	9
127	Kramer et al, 2005	*	*	*	*	*	*	*	*	*	9
128	Agawa et al, 2014	*	*	*	*	*	*	*	*	*	9
129	Chalam et al, 2014	*	*	*	*	*	*	*	*	*	9

No.	Study, year	Selection				Comparability		Exposure			Total no. of stars
		1	2	3	4	1 (a)	1 (b)	1 (a)	2	3	
130	Feng et al, 2018 (a)	*	*	*	*	*	*	*	*	*	9
131	Mimura et al, 2019	*	*	*	*	*	*	*	*	*	9
132	Motohashi et al, 2017	*	*	*	*	*	*	*	*	*	9
133	Ten Berge et al, 2019	*	*	*	*	*	*	*	*	*	9
134	Spindler et al, 2018	*	*	*	*	*	*	*	*	*	9
135	Fauser et al, 2015	*	*	*	*	*	*	*	*	*	9
136	Jonas et al, 2012	*	*	*	*	*	*	*	*	*	9
137	Miao et al, 2012	*	*	*	*	*	-	*	*	*	8
138	Sakurada et al, 2015	*	*	*	*	*	*	*	*	*	9
139	Sato et al, 2018	*	*	*	*	*	*	*	*	*	9
140	Terao et al, 2018	*	*	*	*	*	*	*	*	*	9
141	Fan et al, 2018	*	*	*	*	*	-	*	*	*	8
142	Roh et al, 2010	*	*	*	*	*	*	*	*	*	9
143	Jung et al, 2014	*	*	*	*	*	*	*	*	*	9
144	Shin and Lim, 2011	*	*	*	*	*	*	*	*	*	9
145	Hu et al, 2016	*	*	*	*	*	*	*	*	*	9
146	Asensio-Sánchez et al, 2015	*	*	*	*	*	*	*	*	*	9
147	Garweg et al, 2019	*	*	*	*	*	*	*	*	*	9
148	Kunikata et al, 2013	*	*	*	*	*	-	*	*	*	8
149	Yamamoto et al, 2003	*	*	*	*	*	-	*	*	*	8
150	Sato et al, 2009	*	*	*	*	*	*	*	*	*	9
151	Feng et al, 2017	*	*	*	*	*	-	*	*	*	8
152	Zandi et al, 2016	*	*	*	*	*	*	*	*	*	9
154	Jakobsson et al, 2015	*	*	*	*	*	*	*	*	*	9
155	Sauer et al., 2016	*	*	*	*	*	-	*	*	*	8
156	Suzuki et al, 2019	*	*	*	*	*	*	*	*	*	9
157	Zhu et al, 2016	*	*	*	*	*	*	*	*	*	9
158	Dong et al, 2015 (a)	*	*	*	*	*	*	*	*	*	9
159	Kramer et al, 2012	*	*	*	*	*	*	*	*	*	9

"-" means no star was aasigned

Supplemental Table S10. Checklist of items included in our systematic review according to the Preferred Reporting Items for Systematic Reviews and Meta-Analyses (PRISMA) statement recommendations[4]

Section/topic	#	Checklist item	Reported on page #
**TITLE**			
Title	1	Identify the report as a systematic review, meta-analysis, or both.	1
**ABSTRACT**			
Structured summary	2	Provide a structured summary including, as applicable: background; objectives; data sources; study eligibility criteria, participants, and interventions; study appraisal and synthesis methods; results; limitations; conclusions and implications of key findings; systematic review registration number.	2
**INTRODUCTION**			
Rationale	3	Describe the rationale for the review in the context of what is already known.	2-4
Objectives	4	Provide an explicit statement of questions being addressed with reference to participants, interventions, comparisons, outcomes, and study design (PICOS).	2-4
**METHODS**			
Protocol and registration	5	Indicate if a review protocol exists, if and where it can be accessed (e.g., Web address), and, if available, provide registration information including registration number.	-
Eligibility criteria	6	Specify study characteristics (e.g., PICOS, length of follow-up) and report characteristics (e.g., years considered, language, publication status) used as criteria for eligibility, giving rationale.	16-17
Information sources	7	Describe all information sources (e.g., databases with dates of coverage, contact with study authors to identify additional studies) in the search and date last searched.	16-17
Search	8	Present full electronic search strategy for at least one database, including any limits used, such that it could be repeated.	Appendix 1 (Search strategy) Suppl. p34
Study selection	9	State the process for selecting studies (i.e., screening, eligibility, included in systematic review, and, if applicable, included in the meta-analysis).	16-17
Data collection process	10	Describe method of data extraction from reports (e.g., piloted forms, independently, in duplicate) and any processes for obtaining and confirming data from investigators.	17
Data items	11	List and define all variables for which data were sought (e.g., PICOS, funding sources) and any assumptions and simplifications made.	17
Risk of bias in individual studies	12	Describe methods used for assessing risk of bias of individual studies (including specification of whether this was done at the study or outcome level), and how this information is to be used in any data synthesis.	17-18

Section/topic	#	Checklist item	Reported on page #
Summary measures	13	State the principal summary measures (e.g., risk ratio, difference in means).	17-18
Synthesis of results	14	Describe the methods of handling data and combining results of studies, if done, including measures of consistency (e.g., I^2) for each meta-analysis.	17-18
Risk of bias across studies	15	Specify any assessment of risk of bias that may affect the cumulative evidence (e.g., publication bias, selective reporting within studies).	17-18
Additional analyses	16	Describe methods of additional analyses (e.g., sensitivity or subgroup analyses, meta-regression), if done, indicating which were pre-specified.	17-18
**RESULTS**			
Study selection	17	Give numbers of studies screened, assessed for eligibility, and included in the review, with reasons for exclusions at each stage, ideally with a flow diagram.	4, 5, Fig. 1A, Fig. 1B
Study characteristics	18	For each study, present characteristics for which data were extracted (e.g., study size, PICOS, follow-up period) and provide the citations.	Suppl. Table 1 and 3
Risk of bias within studies	19	Present data on risk of bias of each study and, if available, any outcome level assessment (see item 12).	Suppl. Table 9
Results of individual studies	20	For all outcomes considered (benefits or harms), present, for each study: (a) simple summary data for each intervention group (b) effect estimates and confidence intervals, ideally with a forest plot.	Suppl. Table 2 and 4. Fig. 1C,D, 2-7.
Synthesis of results	21	Present results of each meta-analysis done, including confidence intervals and measures of consistency.	Suppl. Table 2 and 4. Fig. 1C,D, 2-7.
Risk of bias across studies	22	Present results of any assessment of risk of bias across studies (see Item 15).	Suppl. Table 2 and 4
Additional analysis	23	Give results of additional analyses, if done (e.g., sensitivity or subgroup analyses, meta-regression [see Item 16]).	Suppl. Table 2, 4-6, Suppl. Fig. 2-4, 8
**DISCUSSION**			
Summary of evidence	24	Summarize the main findings including the strength of evidence for each main outcome; consider their relevance to key groups (e.g., healthcare providers, users, and policy makers).	11-15
Limitations	25	Discuss limitations at study and outcome level (e.g., risk of bias), and at review-level (e.g., incomplete retrieval of identified research, reporting bias).	15
Conclusions	26	Provide a general interpretation of the results in the context of other evidence, and implications for future research.	16

Section/topic	#	Checklist item	Reported on page #
**FUNDING**			
Funding	27	Describe sources of funding for the systematic review and other support (e.g., supply of data); role of funders for the systematic review.	-

References:

[1]. Hozo, S.P., Djulbegovic, B., & Hozo, I. Estimating the mean and variance from the median, range, and the size of a sample. *BMC Med Res Methodol* **5**, 13 (2005).

[2]. Wan, X., Wang, W., Liu, J., & Tong, T. Estimating the sample mean and standard deviation from the sample size, median, range and/or interquartile range. *BMC Med Res Methodol* **14**, 135 (2014).

[3]. Wells, G.A., Shea, B., O'Connell, D., Peterson, J., Welch, V., Losos, M., *et al*. The Newcastle-Ottawa Scale (NOS) for assessing the quality of nonrandomized studies in meta-analysis. http://www.ohri.ca/programs/clinical_epidemiology/oxford.asp (2011).

[4]. Moher, D., Liberati, A., Tetzlaff, J., & Altman, D.G., PRISMA Group. Preferred reporting items for systematic reviews and meta-analyses: the PRISMA statement. *PLoS Med* **6**, e1000097 (2009).

Appendix 1

Search Strategy

We ran the following search strategy in PubMed until October 2019:

1. “Interleukin-6” [MeSH Terms] OR “Interleukin-6” [All Fields] OR “Interleukin-6” [Title/Abstract]

2. “Polymorphism” [MeSH Terms] OR “Polymorphism” [All Fields] OR “Polymorphism” [Title/Abstract]

3. “Eye disease” [All Fields] OR “Ocular disease” [All Fields] OR “Ophthalmology” [All Fields]

4. “Aqueous” [All Fields] OR “Aqueous” [Title/Abstract]

5. “Vitreous” [All Fields] OR “Vitreous” [Title/Abstract]

6. 1 AND 2 AND 3

7. 1 AND 3

8. 1 AND 4

9. 1 AND 5

10. 1 AND 3 AND 4

11. 1 AND 3 AND 5

Supplemental Figures

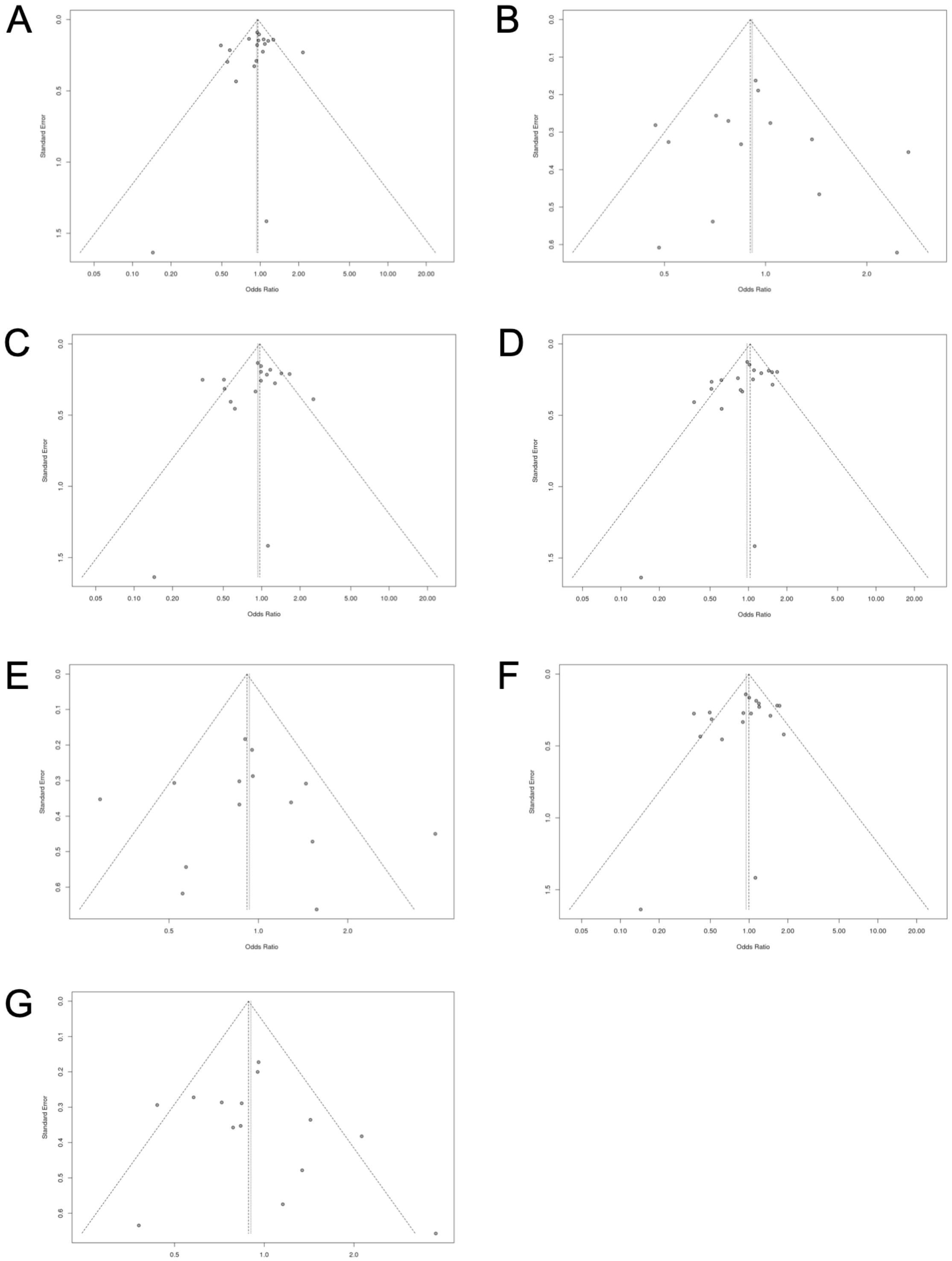

Supplemental Figure S1. A funnel plot analysis of publication bias for IL-6-174 G/C polymorphism and ocular disease. **(A)** Allele model (C vs. G); **(B)** Recessive model (CC vs. GC+GG); **(C)** Dominant model (CC+GC vs. GG); **(D)** Overdominant model (GC vs. CC+GG); **(E)** Homozygous model (CC vs. GG); **(F)** Hetereozygous model (GC vs. GG); **(G)** GC vs. CC model.

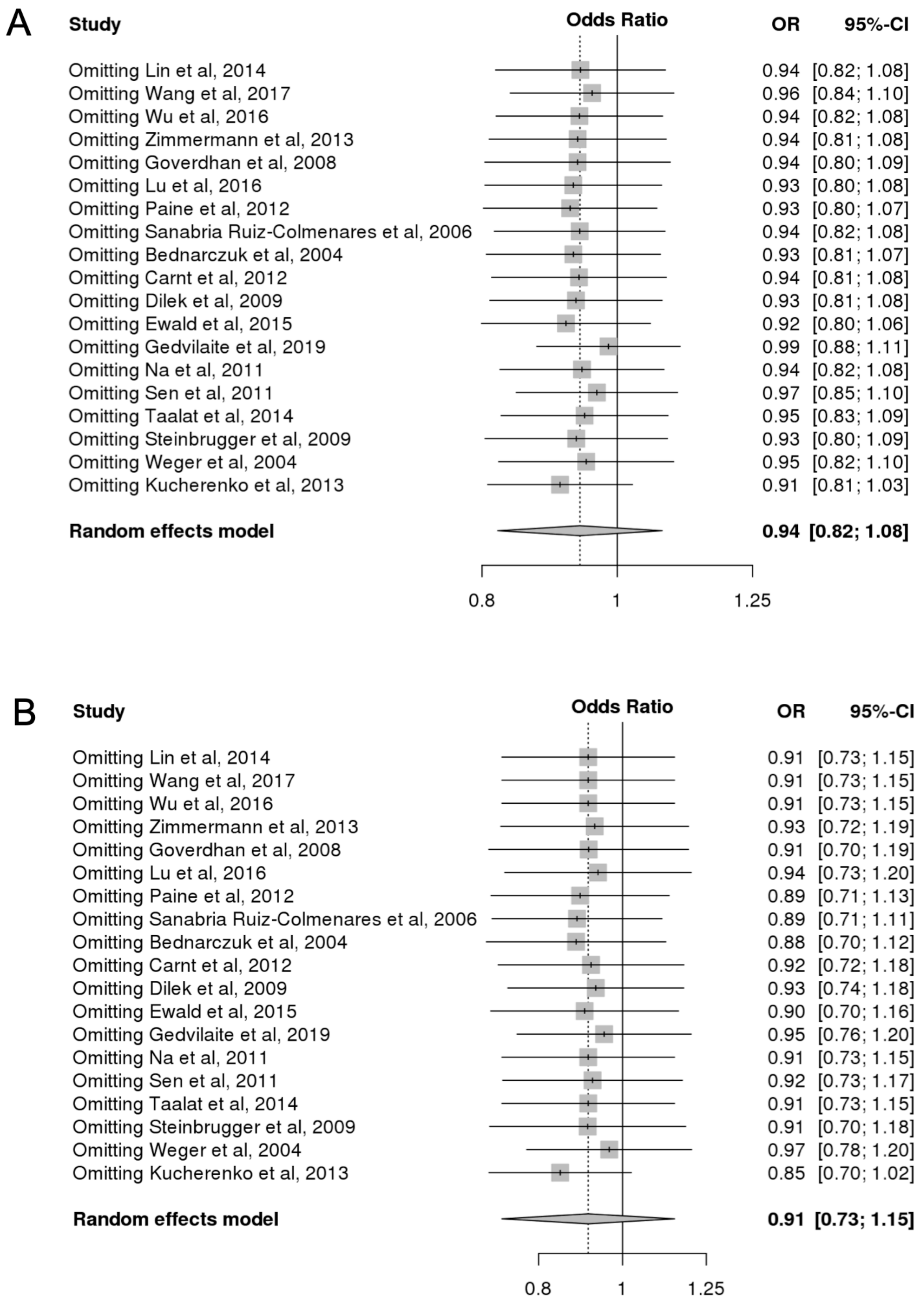

Supplemental Figure S2. A sensitivity analysis for IL-6-174 G/C polymorphism and ocular disease. **(A)** Allele model (C vs. G); **(B)** Recessive model (CC vs. GC+GG).

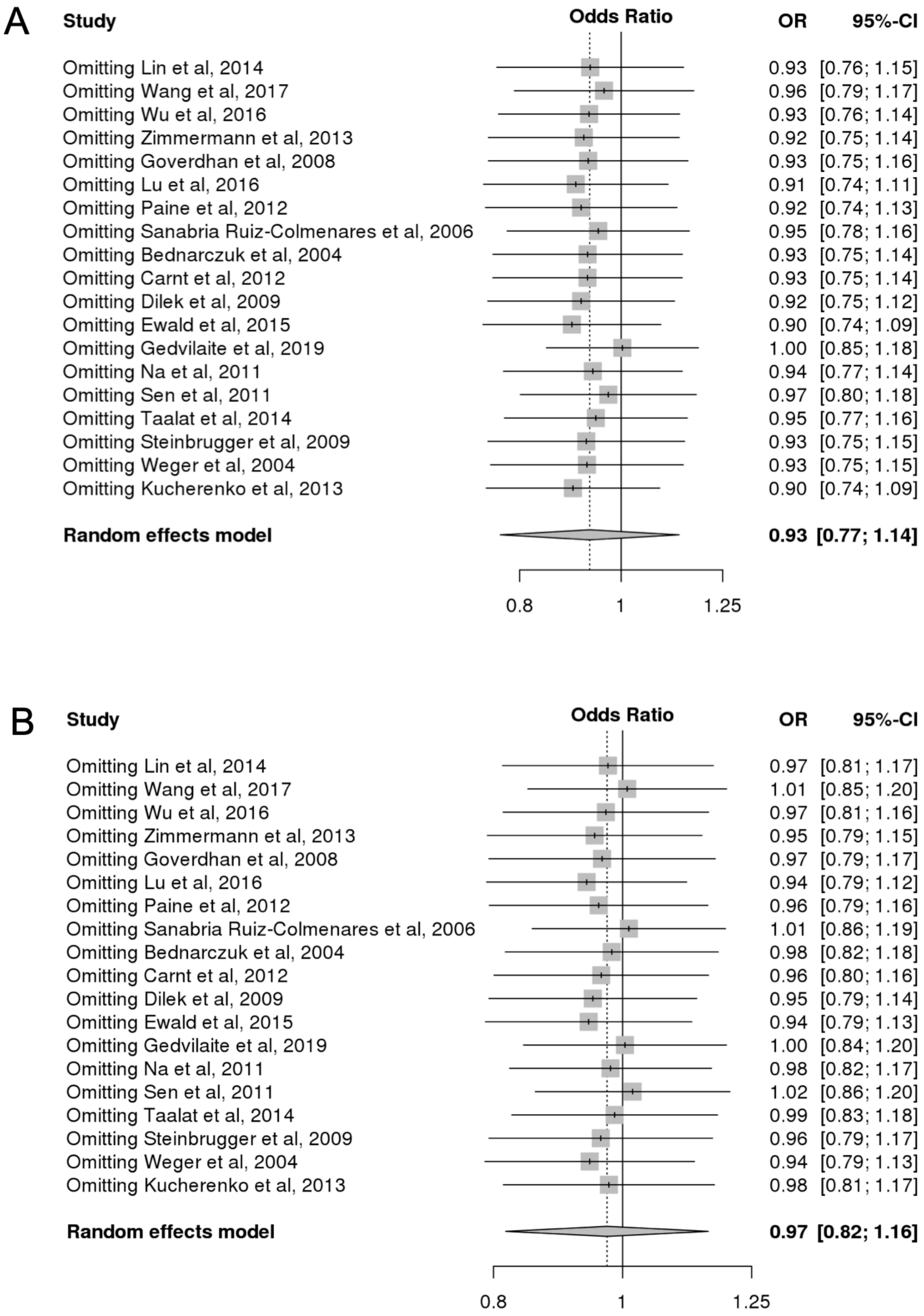

Supplemental Figure S3. A sensitivity analysis for IL-6-174 G/C polymorphism and ocular disease. **(A)** Dominant model (CC+GC vs. GG); **(B)** Overdominant model (GC vs. CC+GG).

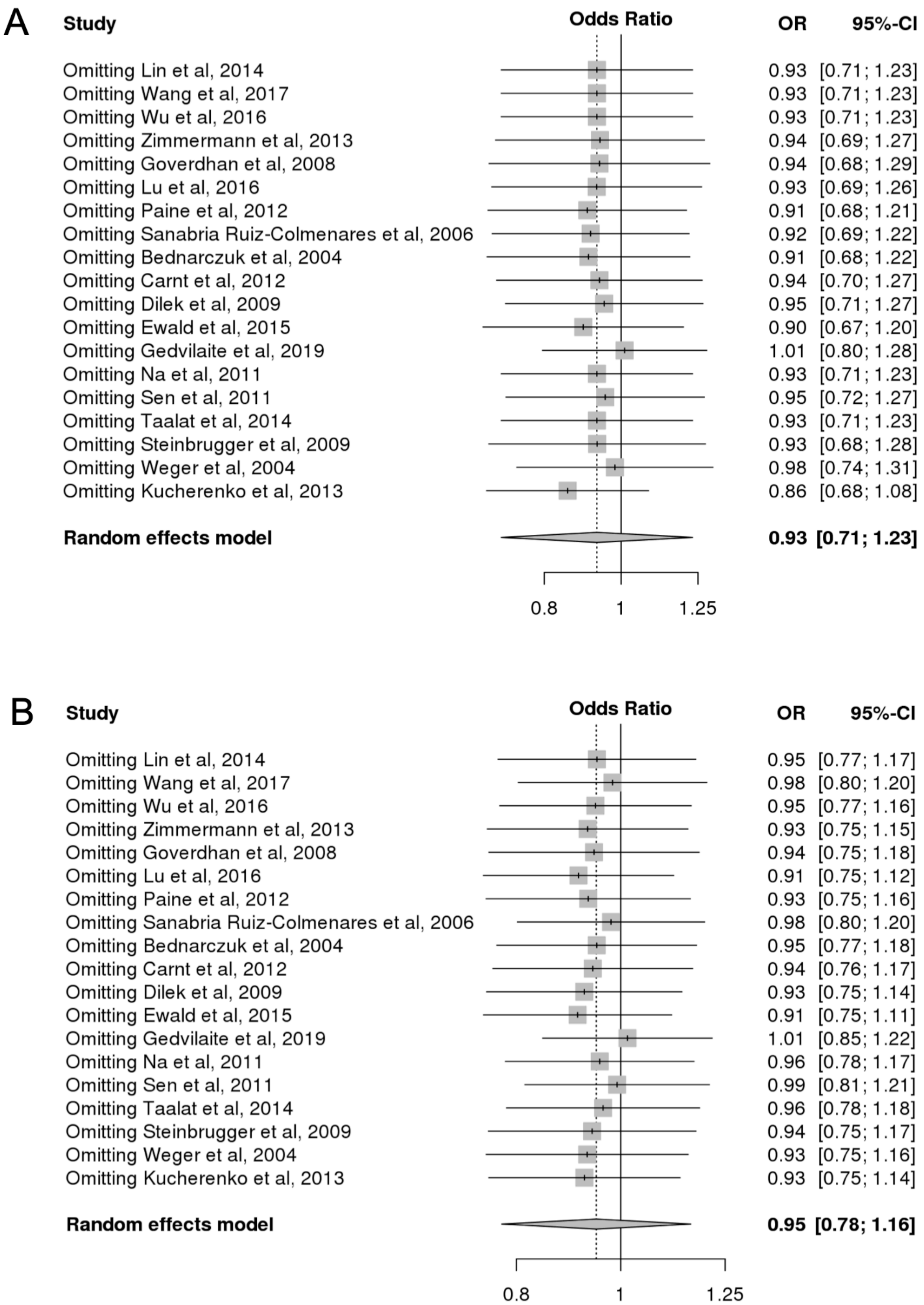

Supplemental Figure S4. A sensitivity analysis for IL-6-174 G/C polymorphism and ocular disease. **(A)** Homozygous model (CC vs. GG); **(B)** Hetereozygous model (GC vs. GG).

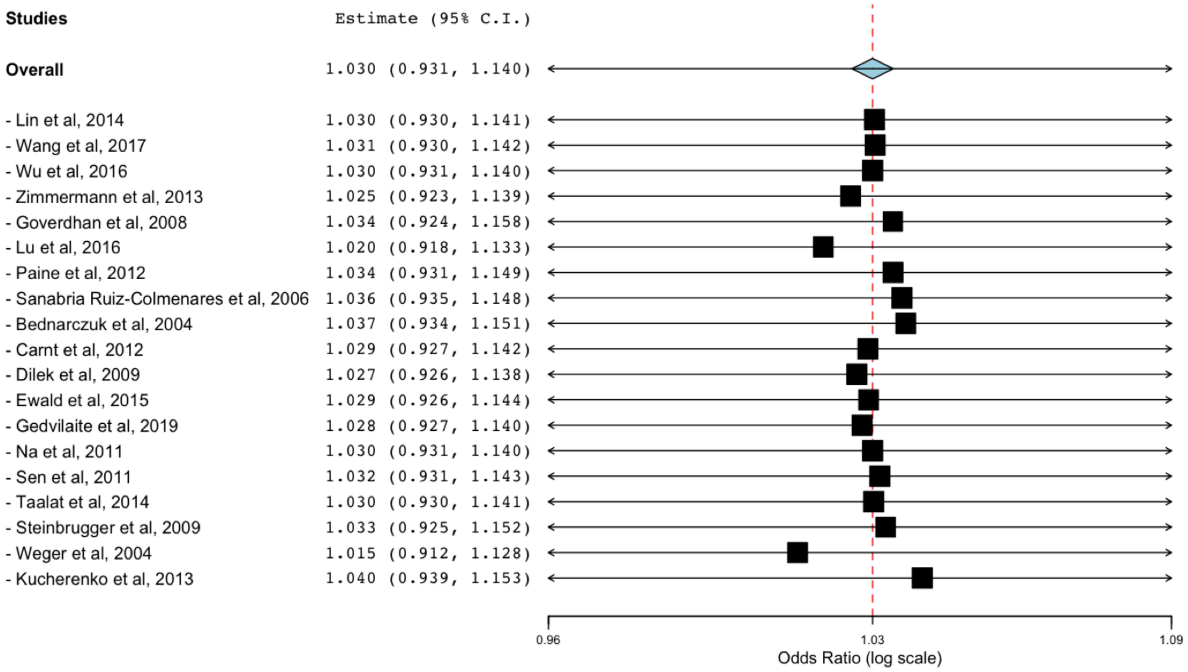

Supplemental Figure S5. A sensitivity analysis of IL-6-174 G/C polymorphism and ocular disease for GC vs. CC model.

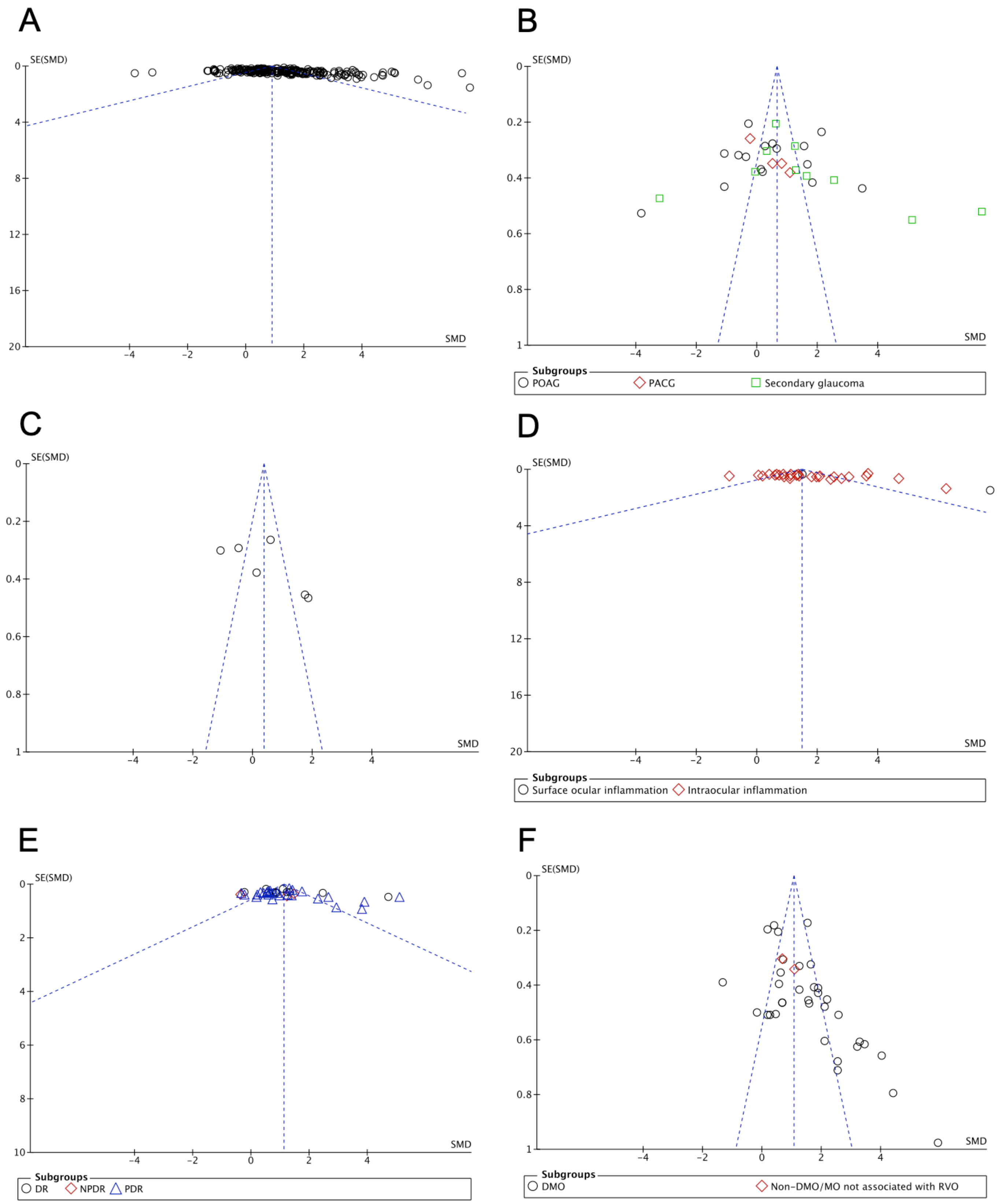

Supplemental Figure S6. A funnel plot analysis of publication bias for intraocular IL-6 levels and ocular diseases. **(A)** Overall; **(B)** Glaucoma; **(C)** PEX syndrome; **(D)** Ocular inflammation; **(E)** DR; **(F)** MO.

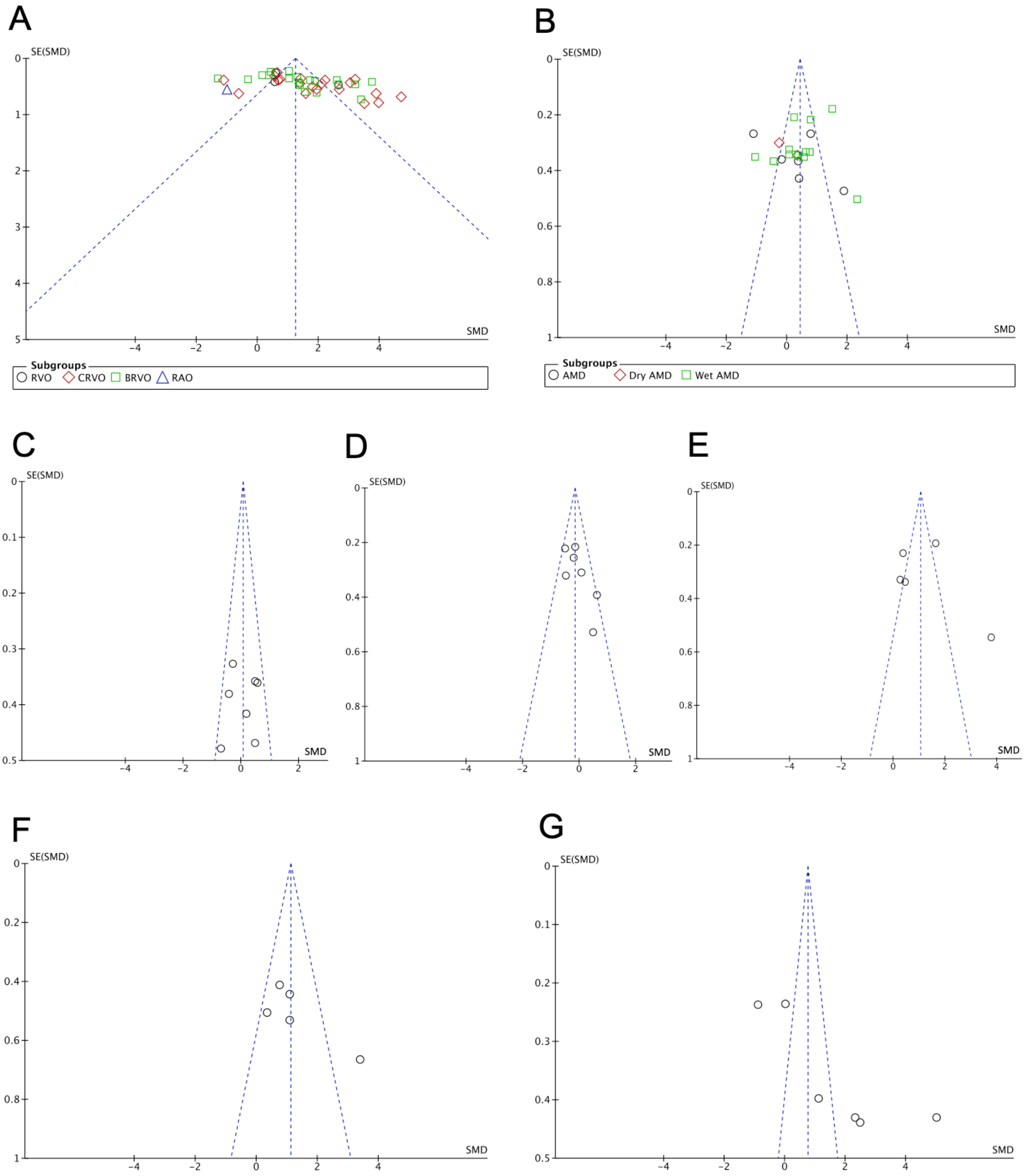

Supplemental Figure S7. A funnel plot analysis of publication bias for intraocular IL-6 levels and ocular diseases. **(A)** Retinal vascular occlusion; **(B)** AMD; **(C)** CNV; **(D)** Pachychoroid spectrum diseases; **(E)** RD; **(F)** PVR; **(G)** Others.

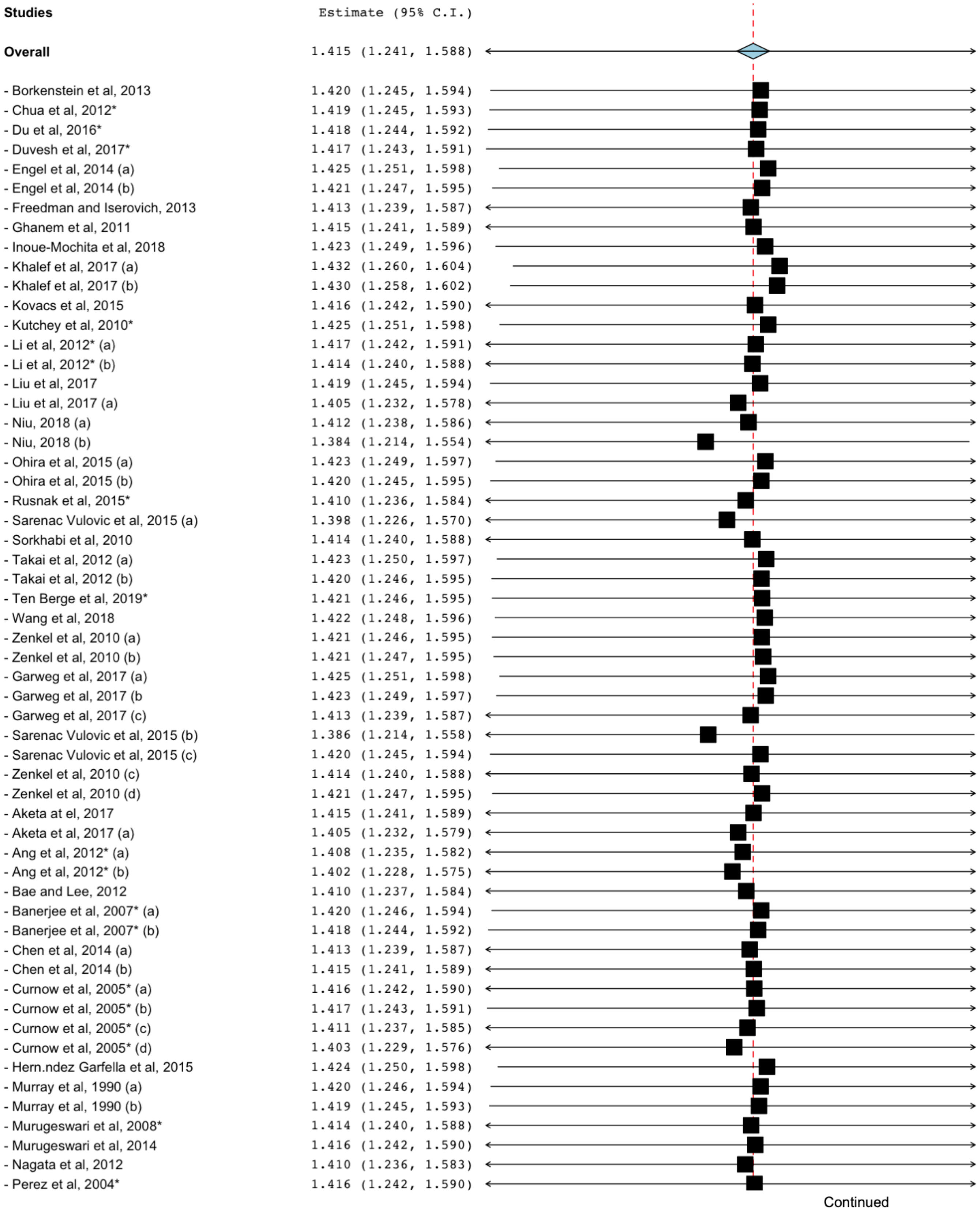

Supplemental Figure S8. A sensitivity analsysis for intraocular IL-6 levels and ocular diseases.

- Petrinovi-Doresi, et al 1999* (a)	1.413 (1.239, 1.587)
- Petrinovi-Doresi, et al 1999* (b)	1.419 (1.245, 1.593)
- Petrinovi-Doresi, et al 1999* (c)	1.412 (1.239, 1.586)
- Sauer et al, 2018*	1.403 (1.230, 1.575)
- Sijssens et al, 2007 (a)	1.421 (1.247, 1.595)
- Sijssens et al, 2007* (b)	1.417 (1.243, 1.591)
- Valentincic et al, 2011*	1.419 (1.245, 1.593)
- van Kooij et al, 2006*	1.418 (1.244, 1.592)
- Wakefield et al, 1995	1.410 (1.238, 1.583)
- Zenkel et al, 2010 (e)	1.416 (1.242, 1.590)
- Zhang et al, 2018	1.400 (1.227, 1.573)
- Abu el Asrar et al, 1992	1.405 (1.232, 1.579)
- Adamiec-Mroczek and Oficjalska-Myczak, 2008	1.410 (1.236, 1.583)
- Banerjee et al, 2007*	1.420 (1.246, 1.594)
- Boss et al, 2017	1.417 (1.242, 1.591)
- Bromberg-White et al, 2013	1.419 (1.245, 1.594)
- Canataroglu et al, 2005	1.415 (1.241, 1.590)
- Chen et al, 2017	1.419 (1.244, 1.594)
- Chernykh et al, 2015	1.410 (1.237, 1.584)
- Cheung et al, 2012*	1.416 (1.242, 1.590)
- Dong et al, 2013*	1.421 (1.246, 1.596)
- Feng et al, 2013 (a)	1.399 (1.227, 1.571)
- Feng et al, 2013 (b)	1.389 (1.217, 1.561)
- Feng et al, 2018 (c)	1.403 (1.231, 1.575)
- Feng et al, 2018 (d)	1.399 (1.227, 1.571)
- Feng et al, 2018 (e)	1.401 (1.229, 1.573)
- Feng et al, 2018 (f)	1.394 (1.222, 1.567)
- Funatsu et al, 2001	1.418 (1.244, 1.593)
- Gustavsson et al, 2012* (a)	1.419 (1.244, 1.593)
- Kauffmann et al, 1994	1.420 (1.246, 1.594)
- Koskela et al, 2013	1.420 (1.246, 1.595)
- Kovacs et al, 2015 (a)	1.420 (1.245, 1.594)
- Loukovaara et al, 2017	1.422 (1.249, 1.596)
- Mastropasqua et al, 2018	1.415 (1.241, 1.589)
- Mocan et al, 2006	1.407 (1.233, 1.580)
- Murugeswari et al, 2008* (a)	1.416 (1.242, 1.590)
- Murugeswari et al, 2014 (a)	1.418 (1.244, 1.592)
- Nakamura et al, 2003	1.369 (1.200, 1.538)
- Oh et al, 2010	1.422 (1.248, 1.596)
- Rusnak et al, 2015* (a)	1.416 (1.242, 1.591)
- Rusnak et al, 2015* (b)	1.419 (1.245, 1.593)
- Schoenberger et al, 2012	1.417 (1.243, 1.591)
- Shimura et al, 2009	1.419 (1.245, 1.593)
- Sin et al, 2013	1.418 (1.244, 1.592)
- Song et al, 2014 (a)	1.422 (1.248, 1.596)
- Song et al, 2014 (b)	1.422 (1.248, 1.596)
- Srividya et al, 2018	1.410 (1.236, 1.584)
- Suzuki et al, 2011	1.419 (1.245, 1.593)
- Takahashi et al, 2016	1.420 (1.245, 1.594)
- Tsai et al, 2018	1.420 (1.246, 1.594)
- Wu et al, 2017 (a)	1.412 (1.238, 1.585)
- Wu et al, 2017 (b)	1.416 (1.242, 1.590)
- Xu et al, 2015	1.397 (1.225, 1.570)
- Yoshida et al, 2015	1.381 (1.211, 1.552)
- Yoshimura et al, 2009*	1.419 (1.243, 1.594)
- Yuuki et al, 2001	1.399 (1.227, 1.572)
- Zeng et al, 2019* (a)	1.418 (1.244, 1.592)
- Zeng et al, 2019* (b)	1.419 (1.244, 1.593)
- Zhou et al, 2012*	1.414 (1.240, 1.588)
- Bandyopadhyay et al, 2018* (a)	1.413 (1.239, 1.587)
- Bandyopadhyay et al, 2018* (b)	1.413 (1.239, 1.587)

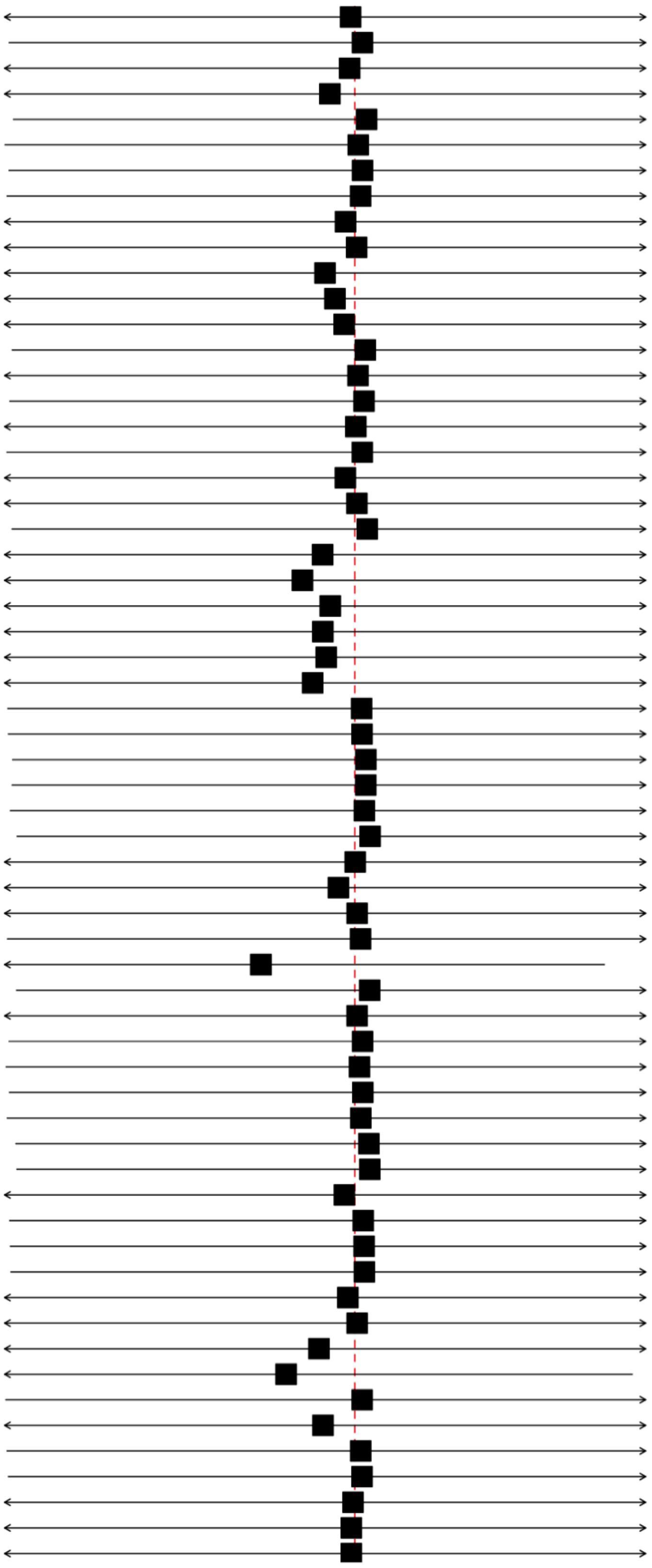

Continued

- Bandyopadhyay et al, 2018* (c)	1.404 (1.231, 1.578)
- Bayomy and Elgouhary, 2018* (a)	1.412 (1.238, 1.586)
- Bayomy and Elgouhary, 2018* (b)	1.407 (1.233, 1.580)
- Chen et al, 2016*	1.422 (1.247, 1.596)
- Chu et al, 2013*	1.419 (1.245, 1.593)
- Dong et al, 2015* (a)	1.420 (1.246, 1.595)
- Dong et al, 2015* (b)	1.421 (1.247, 1.596)
- Funatsu et al, 2002 (a)*	1.416 (1.242, 1.591)
- Funatsu et al, 2002 (b)*	1.412 (1.238, 1.586)
- Funatsu et al, 2003*	1.414 (1.240, 1.588)
- Funatsu et al, 2009*	1.415 (1.240, 1.589)
- Funk et al, 2010	1.418 (1.244, 1.592)
- Jonas et al, 2012	1.419 (1.245, 1.593)
- Lee et al, 2012	1.419 (1.245, 1.593)
- Noma et al, 2012* (a)	1.412 (1.238, 1.586)
- Noma et al, 2012* (b)	1.408 (1.234, 1.581)
- Noma et al, 2017*	1.411 (1.237, 1.584)
- Roh et al, 2009 (a)	1.418 (1.244, 1.592)
- Roh et al, 2009 (b)	1.412 (1.239, 1.586)
- Sohn et al, 2011* (a)	1.420 (1.246, 1.594)
- Sohn et al, 2011* (b)	1.420 (1.246, 1.594)
- Sonoda et al, 2013	1.419 (1.245, 1.593)
- Srividya et al, 2018 (a)	1.411 (1.237, 1.585)
- van Kooij et al, 2006* (a)	1.417 (1.243, 1.591)
- Wen et al, 2015	1.408 (1.234, 1.581)
- Yoshida et al, 2015 (a)	1.425 (1.252, 1.599)
- Yoshimura et al, 2009* (a)	1.416 (1.242, 1.591)
- Yu et al, 2018 (a)*	1.419 (1.245, 1.593)
- Yu et al, 2018 (b)*	1.421 (1.248, 1.595)
- Chen et al, 1999 (a)	1.422 (1.249, 1.596)
- Chen et al, 1999 (b)	1.401 (1.228, 1.574)
- Chen et al, 1999 (c)	1.414 (1.241, 1.588)
- Ehlken et al, 2015	1.415 (1.241, 1.589)
- Feng et al, 2013 (a)	1.410 (1.236, 1.584)
- Feng et al, 2013 (b)	1.410 (1.236, 1.584)
- Feng et al, 2013 (c)	1.407 (1.234, 1.581)
- Funk et al, 2009* (a)	1.413 (1.239, 1.587)
- Funk et al, 2009* (b)	1.415 (1.241, 1.589)
- Jung et al, 2014	1.420 (1.245, 1.594)
- Kaneda et al, 2011	1.404 (1.231, 1.577)
- Koss et al, 2012 (a)	1.419 (1.245, 1.593)
- Koss et al, 2012 (b)	1.418 (1.244, 1.593)
- Koss et al, 2012 (c)	1.420 (1.246, 1.594)
- Koss et al, 2013	1.419 (1.245, 1.594)
- Kramer et al, 2005	1.424 (1.250, 1.598)
- Lee et al, 2012 (a)	1.422 (1.248, 1.596)
- Lim, 2011	1.417 (1.243, 1.591)
- Noma et al, 2005*	1.415 (1.241, 1.589)
- Noma et al, 2006*	1.414 (1.240, 1.588)
- Noma et al, 2009*	1.412 (1.238, 1.586)
- Noma et al, 2010*	1.414 (1.240, 1.588)
- Noma et al, 2013* (a)	1.405 (1.231, 1.578)
- Noma et al, 2013* (b)	1.406 (1.232, 1.579)
- Noma et al, 2013* (c)	1.410 (1.236, 1.583)
- Noma et al, 2014* (a)	1.407 (1.234, 1.580)
- Noma et al, 2014* (b)	1.410 (1.236, 1.583)
- Noma et al, 2015*	1.408 (1.234, 1.581)
- Noma et al, 2016*	1.413 (1.239, 1.587)
- Park and Ahn, 2009	1.415 (1.241, 1.590)
- Pfister et al, 2013	1.420 (1.246, 1.595)
- Rezar-Dreindl et al, 2016* (a)	1.425 (1.251, 1.598)

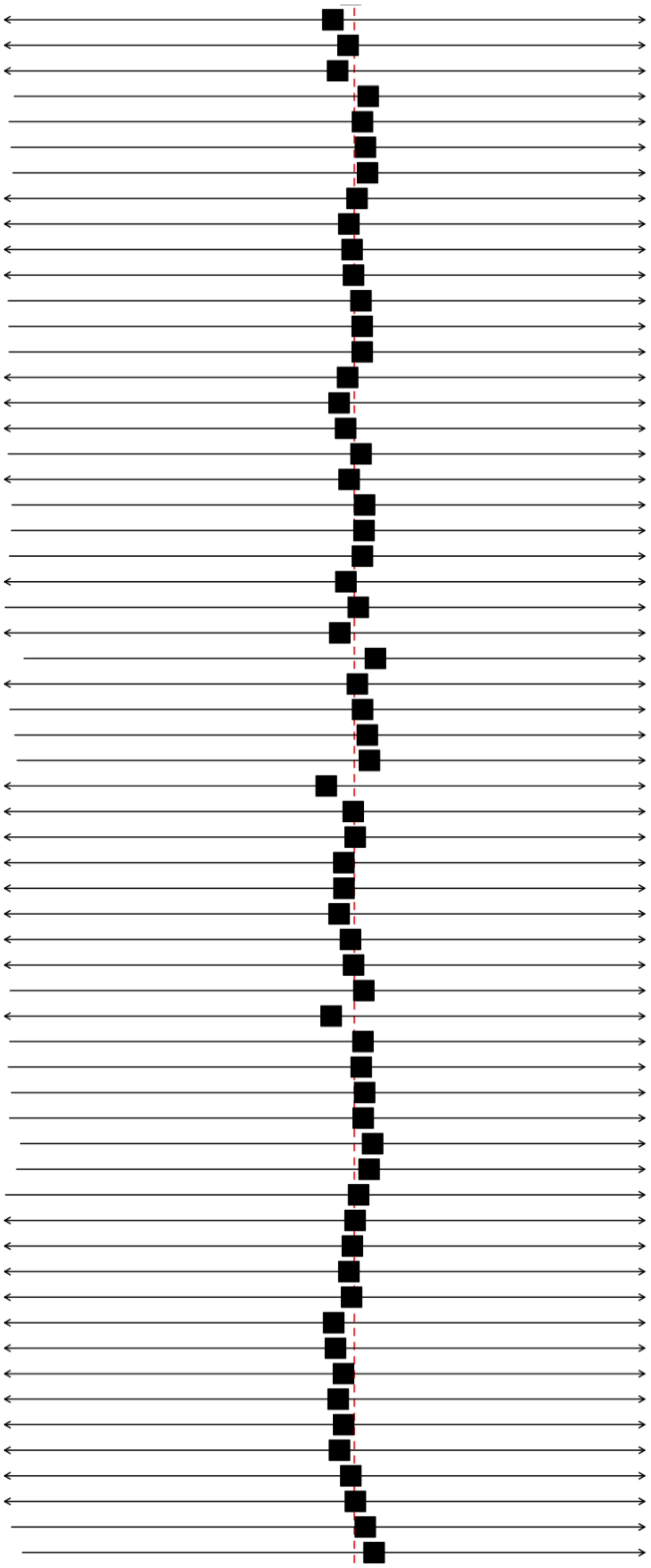

Continued

- Rezar-Dreindl et al, 2016* (b)	1.425 (1.252, 1.599)
- Shchuko et al, 2015 (a)	1.416 (1.241, 1.590)
- Shchuko et al, 2015 (b)	1.421 (1.247, 1.595)
- Shimura et al, 2008	1.416 (1.242, 1.590)
- Sohn et al, 2014* (a)	1.413 (1.239, 1.587)
- Sohn et al, 2014* (b)	1.400 (1.227, 1.573)
- Suzuki et al, 2011	1.419 (1.244, 1.593)
- Takahashi et al, 2016 (a)	1.419 (1.245, 1.593)
- Wen et al, 2015 (a)	1.402 (1.229, 1.575)
- Yoshimura et al, 2009* (b)	1.406 (1.233, 1.580)
- Yoshimura et al, 2009* (c)	1.418 (1.243, 1.593)
- Agawa et al, 2014	1.424 (1.251, 1.598)
- Chalam et al, 2014	1.419 (1.244, 1.593)
- Fauser et al, 2015	1.420 (1.246, 1.594)
- Feng et al, 2018	1.419 (1.245, 1.593)
- Jonas et al, 2012 (a)	1.419 (1.245, 1.593)
- Kramer et al, 2012 (a)	1.420 (1.246, 1.594)
- Kramer et al, 2012 (b)	1.419 (1.245, 1.593)
- Miao et al, 2012	1.423 (1.249, 1.597)
- Mimura et al, 2019*	1.413 (1.239, 1.587)
- Motohashi et al, 2017	1.420 (1.246, 1.594)
- Rezar-Dreindl et al, 2016 (a)	1.420 (1.246, 1.594)
- Rezar-Dreindl et al, 2016 (b)	1.421 (1.247, 1.595)
- Sakurada et al, 2015	1.421 (1.247, 1.595)
- Sato et al, 2018	1.425 (1.251, 1.598)
- Spindler et al, 2018 (a)	1.422 (1.248, 1.596)
- Spindler et al, 2018 (b)	1.418 (1.244, 1.593)
- Ten Berge et al, 2019* (a)	1.422 (1.248, 1.596)
- Terao et al, 2018*	1.411 (1.238, 1.585)
- Fan et al, 2018	1.423 (1.249, 1.596)
- Miao et al, 2012 (a)	1.423 (1.250, 1.597)
- Miao et al, 2012 (b)	1.420 (1.246, 1.594)
- Roh et al, 2009 (c)	1.422 (1.248, 1.596)
- Roh et al, 2009 (d)	1.419 (1.245, 1.593)
- Roh et al, 2009 (e)	1.419 (1.245, 1.594)
- Roh et al, 2010	1.419 (1.245, 1.593)
- Feng et al, 2018	1.419 (1.245, 1.593)
- Hu et al, 2016 (a)	1.422 (1.248, 1.597)
- Hu et al, 2016 (b)	1.423 (1.249, 1.597)
- Jung et al, 2014	1.422 (1.248, 1.596)
- Sakurada et al, 2015	1.421 (1.247, 1.595)
- Shin and Lim, 2011	1.423 (1.249, 1.597)
- Terao et al, 2018*	1.419 (1.245, 1.593)
- Asensio-S.nchez et al, 2015	1.405 (1.231, 1.578)
- Garweg et al, 2019	1.421 (1.246, 1.595)
- Kunikata et al, 2013	1.420 (1.245, 1.594)
- Takahashi et al, 2016	1.420 (1.246, 1.595)
- Yoshimura et al, 2009*	1.415 (1.241, 1.590)
- Banerjee et al, 2017	1.419 (1.245, 1.593)
- Canataroglu et al, 2005	1.417 (1.243, 1.591)
- Canataroglu et al, 2005 (a)	1.407 (1.234, 1.581)
- Kauffmann et al, 1994	1.418 (1.244, 1.592)
- Yamamoto et al, 2003	1.416 (1.243, 1.590)
- Sato et al, 2009* (a)	1.409 (1.235, 1.582)
- Sato et al, 2009* (b)	1.415 (1.241, 1.589)
- Feng et al, 2016* (a)	1.414 (1.241, 1.588)
- Feng et al, 2016* (b)	1.415 (1.241, 1.588)
- Ten Berge et al, 2019*	1.423 (1.249, 1.596)
- Yoshida et al, 2013	1.413 (1.239, 1.587)
- Takahashi et al, 2016	1.421 (1.247, 1.595)
- Zandi et al, 2016	1.414 (1.240, 1.588)

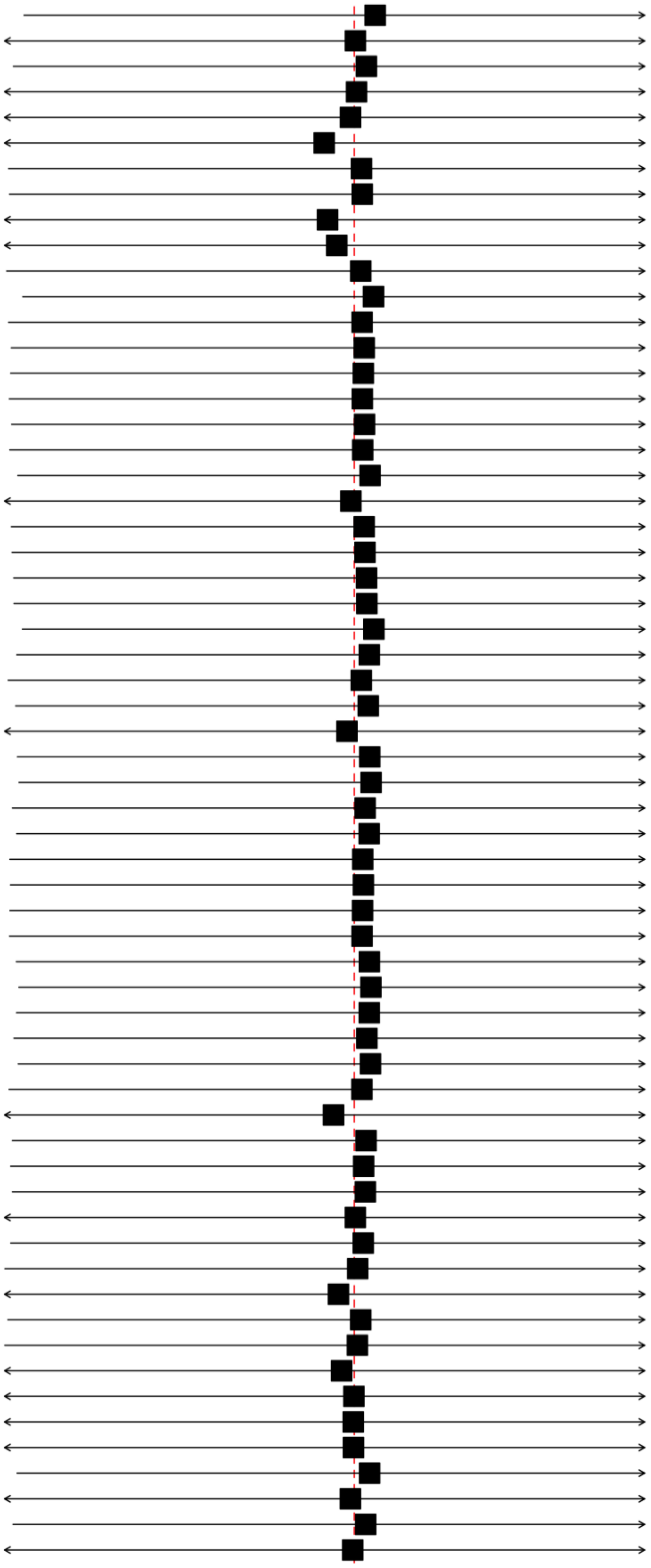

Continued

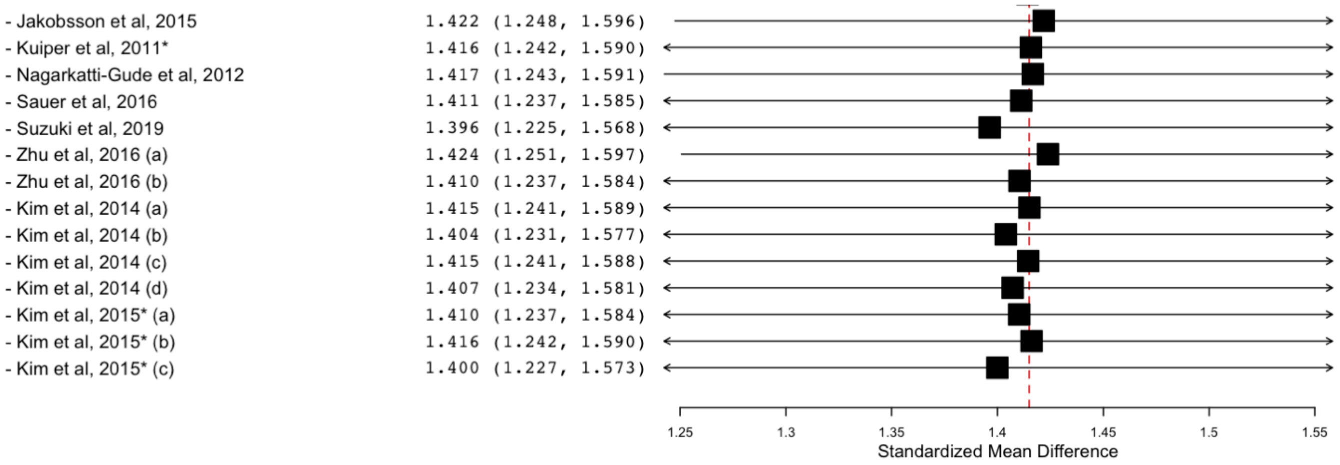

Supplemental Figure S8. A sensitivity analsysis for intraocular IL-6 levels and ocular diseases.

Study or Subgroup	Early PEX Mean	Early PEX SD	Early PEX Total	Late PEX Mean	Late PEX SD	Late PEX Total	Weight	Std. Mean Difference IV, Random, 95% CI
Garweg et al, 2017 (a)	2.2	0.8	33	3.1	1.5	30	25.6%	−0.75 [−1.26, −0.24]
Garweg et al, 2017 (b)	2.2	0.8	33	118.6	104.9	10	25.2%	−2.32 [−3.20, −1.45]
Sarenac Vulovic et al, 2015	627.05	65.69	30	162.86	38.37	30	24.0%	8.52 [6.86, 10.17]
Zenkel et al, 2010	186	99	14	57	32	14	25.2%	1.70 [0.82, 2.59]
**Total (95% CI)**			**110**			**84**	**100.0%**	**1.69 [−1.36, 4.74]**

Heterogeneity: $Tau^2 = 9.40$; $Chi^2 = 151.02$, df = 3 ($P < 0.00001$); $I^2 = 98\%$
Test for overall effect: Z = 1.09 (P = 0.28)

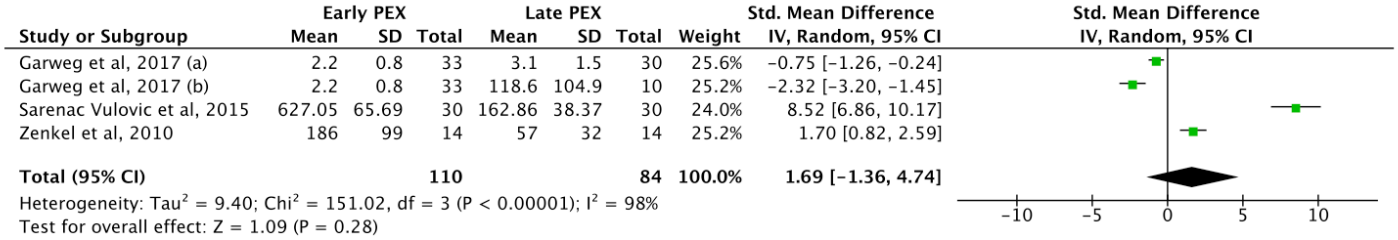

Supplemental Figure S9. Forest plot for pooled SMD and 95% CI for IL-6 levels between early and late PEX syndrome.

Study or Subgroup	Case Mean	Case SD	Case Total	Control Mean	Control SD	Control Total	Weight	Std. Mean Difference IV, Random, 95% CI
**1.1.1 Surface ocular disease**								
Acere et al, 2008 (c)	16.52	10.58	20	8.15	2.73	18	2.0%	1.04 [0.35, 1.72]
Acere et al, 2008 (d)	33.11	8.66	11	8.15	2.73	18	1.7%	4.26 [2.86, 5.65]
Fodor et al, 2006 (a)	109	72	7	119	142	52	2.0%	−0.07 [−0.86, 0.72]
Jun et al, 2011	282.7	467.7	18	89.5	83.5	11	2.0%	0.50 [−0.26, 1.26]
Matsumura et al, 2017	666	532	15	28	46.3	15	2.0%	1.64 [0.80, 2.49]
Oray and Toker, 2013	236.2	207.4	21	107.6	99.4	15	2.0%	0.73 [0.05, 1.42]
Saeki et al., 2017 (a)	15.8	15.8	22	8.3	8.3	13	2.0%	0.54 [−0.16, 1.24]
Saeki et al., 2017 (c)	21.2	22.8	14	8.3	8.3	13	2.0%	0.72 [−0.06, 1.50]
Saeki et al., 2017 (d)	48.2	38.3	21	8.3	8.3	13	2.0%	1.27 [0.51, 2.03]
Sakimoto et al, 2014	1,394	1,399	16	17.5	20.3	12	2.0%	1.26 [0.43, 2.09]
Tai et al, 2019*	1,018.62	373	25	74.36	82.06	20	2.0%	3.27 [2.35, 4.19]
Ujhelyi et al, 2012*	752.51	25.65	54	457.9	21.44	24	1.4%	11.93 [9.95, 13.91]
Van Acker et al, 2019 (a)	27	8	14	17	2.1	37	2.0%	2.19 [1.43, 2.95]
Van Acker et al, 2019 (b)	41.5	13.5	7	17	2.1	37	1.8%	4.41 [3.15, 5.66]
Van Acker et al, 2019 (c)	30.8	16	5	17	2.1	37	1.9%	2.49 [1.40, 3.58]
**Subtotal (95% CI)**			**270**			**335**	**29.0%**	**2.22 [1.38, 3.06]**
Heterogeneity: $Tau^2 = 2.53$; $Chi^2 = 202.75$, df = 14 (P < 0.00001); $I^2 = 93\%$								
Test for overall effect: Z = 5.15 (P < 0.00001)								
**1.1.2 KC**								
Lema and Durán, 2005	29	1.5	28	9.5	0.775	20	0.9%	15.31 [12.07, 18.56]
Lema et al, 2009 (a)	22.8	0.5	30	9.5	0.775	20	0.6%	21.00 [16.67, 25.32]
Lema et al, 2009 (b)	22.1	0.425	30	9.5	0.775	20	0.6%	21.06 [16.72, 25.39]
Shetty et al, 2015	4,050	730	12	2,650	890	10	1.9%	1.67 [0.67, 2.67]
Shetty et al, 2017	2,413	489	7	49	21	6	1.0%	6.08 [3.08, 9.09]
Sorkhabi et al, 2014	17.49	1.92	42	13.81	1.71	30	2.1%	1.98 [1.41, 2.56]
**Subtotal (95% CI)**			**149**			**106**	**7.1%**	**10.66 [6.33, 14.99]**
Heterogeneity: $Tau^2 = 26.87$; $Chi^2 = 210.31$, df = 5 (P < 0.00001); $I^2 = 98\%$								
Test for overall effect: Z = 4.83 (P < 0.00001)								
**1.1.3 CCh**								
Acere et al, 2008 (a)	51.78	81.53	25	8.15	2.73	18	2.1%	0.69 [0.06, 1.31]
Erdogan-Poyraz et al, 2009 (a)	14.9	2.3	16	13.9	1.1	10	2.0%	0.50 [−0.30, 1.30]
Erdogan-Poyraz et al, 2009 (b)	32.4	14	21	13.9	1.1	10	2.0%	1.55 [0.69, 2.41]
Erdogan-Poyraz et al, 2009 (c)	184.4	155.4	14	13.9	1.1	10	2.0%	1.38 [0.46, 2.29]
**Subtotal (95% CI)**			**76**			**48**	**8.0%**	**0.97 [0.49, 1.46]**
Heterogeneity: $Tau^2 = 0.09$; $Chi^2 = 4.59$, df = 3 (P = 0.20); $I^2 = 35\%$								
Test for overall effect: Z = 3.91 (P < 0.0001)								
**1.1.4 Conjungtivitis**								
Fodor et al, 2006 (b)	366	296	11	119	142	52	2.0%	1.38 [0.68, 2.08]
Leonardi et al, 1998 (a)	28	43.9	10	0.5	0.5	14	2.0%	0.95 [0.08, 1.81]
Leonardi et al, 1998 (b)	58.5	161.3	9	0.5	0.5	14	2.0%	0.56 [−0.29, 1.42]
Nivenius et al, 2011* (a)	778	25.5	11	286	10	5	0.2%	20.95 [12.52, 29.38]
Nivenius et al, 2011* (b)	613	15.25	5	286	10	5	0.1%	22.90 [9.95, 35.86]
Pflugfelder et al, 1999	3,690	3,200	10	290	500	10	1.9%	1.42 [0.42, 2.43]
Saeki et al., 2017 (b)	11.5	11.1	14	8.3	8.3	13	2.0%	0.31 [−0.45, 1.08]
**Subtotal (95% CI)**			**70**			**113**	**10.2%**	**1.39 [0.29, 2.49]**
Heterogeneity: $Tau^2 = 1.43$; $Chi^2 = 38.45$, df = 6 (P < 0.00001); $I^2 = 84\%$								
Test for overall effect: Z = 2.48 (P = 0.01)								
**1.1.5 Asymptomatic CL**								
Chao et al, 2018*	17.3	1.025	9	16.1	1.025	9	1.9%	1.11 [0.10, 2.13]
Elgin et al, 2015 (a)	2,643.7	259.49	9	2,634.99	251.99	9	2.0%	0.03 [−0.89, 0.96]
Elgin et al, 2015 (b)	2,387.43	58.74	9	2,634.99	251.99	9	1.9%	−1.29 [−2.33, −0.25]
Gonzalez-Perez et al, 2012 (a)	2.6	0.6	28	2.2	0.5	32	2.1%	0.72 [0.19, 1.24]
Gonzalez-Perez et al, 2012 (b)	4.7	1.2	32	2.2	0.5	32	2.0%	2.69 [2.00, 3.37]
**Subtotal (95% CI)**			**87**			**91**	**9.9%**	**0.68 [−0.53, 1.90]**
Heterogeneity: $Tau^2 = 1.73$; $Chi^2 = 47.04$, df = 4 (P < 0.00001); $I^2 = 91\%$								
Test for overall effect: Z = 1.10 (P = 0.27)								
**1.1.6 Dry eye releted disease**								
Acere et al, 2008 (b)	16.53	1.26	13	8.15	2.73	18	1.8%	3.64 [2.44, 4.84]
Benitez-del-Castillo et al, 2018	18	6.4	30	12.1	8.6	36	2.1%	0.76 [0.26, 1.26]
Jackson et al, 2016	52.8	18.1	62	45.1	6.2	15	2.1%	0.46 [−0.11, 1.03]
Jung et al, 2016	87.53	41.46	15	47.94	31.07	35	2.1%	1.13 [0.48, 1.78]
Lam et al, 2009	238	278.2	30	26.5	21.8	14	2.1%	0.90 [0.23, 1.56]
Landsend et al, 2018	6.5	6.6	35	1.7	1.8	21	2.1%	0.88 [0.32, 1.45]
Lee et al, 2013 (a)	19,220	20,110	40	6,970	6,730	35	2.1%	0.79 [0.32, 1.26]
Lee et al, 2013 (b)	12,120	13,540	40	6,970	6,730	35	2.1%	0.47 [0.01, 0.93]
Massingale et al, 2009	1,625.7	430.9	7	632.3	167.9	7	1.6%	2.84 [1.22, 4.47]
McDonnell et al, 2017	147.45	475.145	217	104.72	290.994	67	2.1%	0.10 [−0.18, 0.37]
Mrugacz et al, 2017	27,790	11,280	32	6,470	2,100	34	2.0%	2.64 [1.97, 3.30]
Tishler et al, 1999	88.6	16.2	12	42.1	10.6	12	1.8%	3.28 [1.99, 4.57]
Zhao et al, 2018 (a)	9.46	9.16	20	2.79	4.31	15	2.0%	0.87 [0.17, 1.57]
Zhao et al, 2018 (b)	6.78	7.63	20	2.79	4.31	15	2.0%	0.61 [−0.08, 1.29]
Zhao et al, 2018 (c)	9.19	9.98	15	2.79	4.31	15	2.0%	0.81 [0.06, 1.56]
**Subtotal (95% CI)**			**588**			**374**	**30.1%**	**1.18 [0.76, 1.60]**
Heterogeneity: $Tau^2 = 0.54$; $Chi^2 = 100.02$, df = 14 (P < 0.00001); $I^2 = 86\%$								
Test for overall effect: Z = 5.50 (P < 0.00001)								
**1.1.7 Keratitis**								
Santacruz et al, 2015 (a)	4,172	1,873	14	25	17	48	1.9%	4.70 [3.66, 5.74]
Santacruz et al, 2015 (b)	758	1,166	8	25	17	48	2.0%	1.72 [0.90, 2.54]
Santacruz et al, 2015 (c)	1,596	971	6	25	17	48	1.8%	5.13 [3.82, 6.45]
**Subtotal (95% CI)**			**28**			**144**	**5.7%**	**3.81 [1.54, 6.09]**
Heterogeneity: $Tau^2 = 3.75$; $Chi^2 = 28.69$, df = 2 (P < 0.00001); $I^2 = 93\%$								
Test for overall effect: Z = 3.28 (P = 0.001)								
**Total (95% CI)**			**1268**			**1211**	**100.0%**	**2.07 [1.67, 2.48]**
Heterogeneity: $Tau^2 = 1.97$; $Chi^2 = 770.75$, df = 54 (P < 0.00001); $I^2 = 93\%$								
Test for overall effect: Z = 10.02 (P < 0.00001)								
Test for subgroup differences: $Chi^2 = 30.26$, df = 6 (P < 0.0001), $I^2 = 80.2\%$								

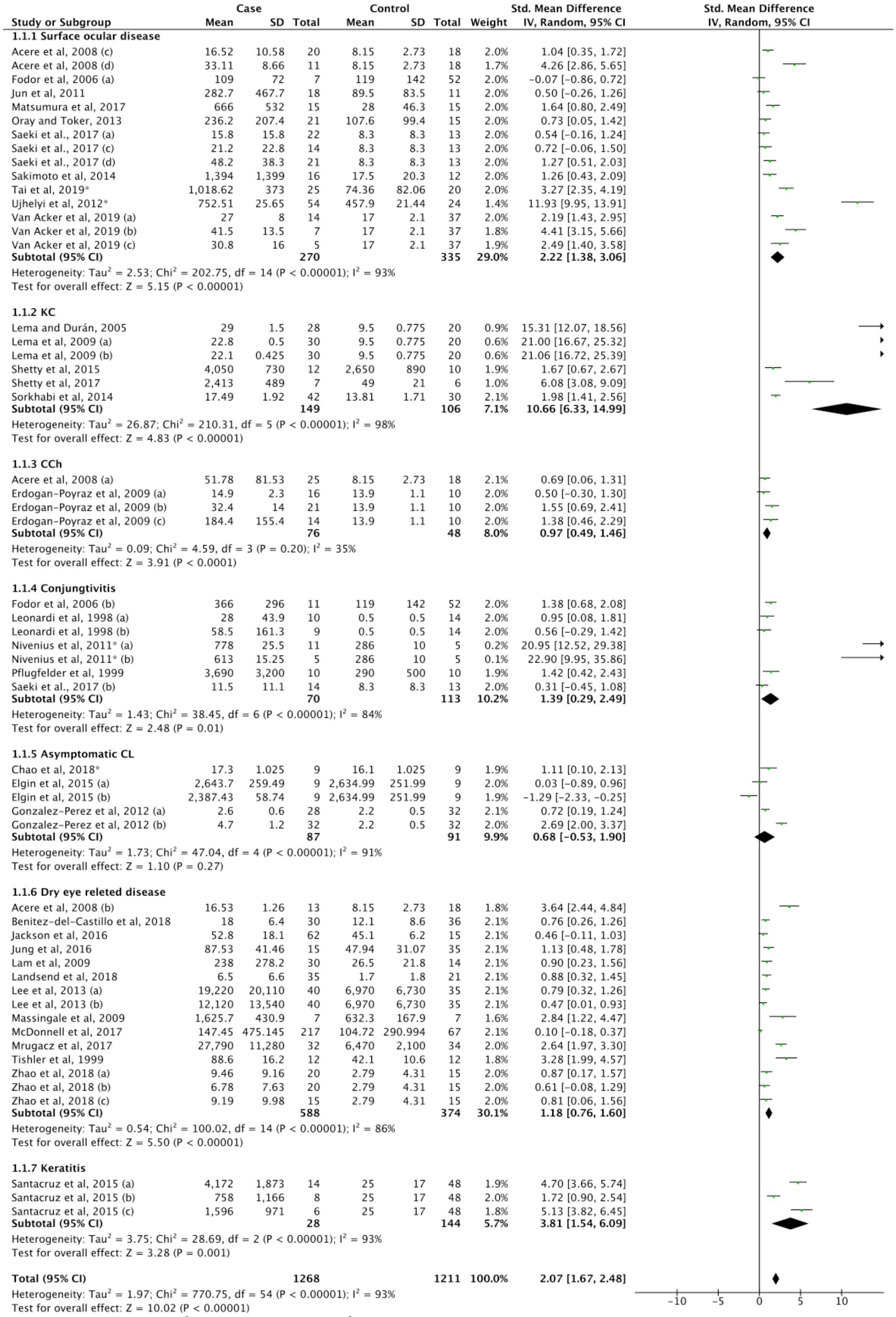

Supplemental Figure S10. Forest plot for pooled SMD and 95% CI for IL-6 levels between surface ocular disease and control.

Study or Subgroup	Case Mean	Case SD	Case Total	Control Mean	Control SD	Control Total	Weight	Std. Mean Difference IV, Random, 95% CI
**1.1.1 Uveitis**								
Banerjee et al, 2007* (a)	145	1,004.75	8	6	6.25	8	3.4%	0.18 [−0.80, 1.17]
Hernández Garfella et al, 2015	109.8	88.1	6	333.38	274.83	12	3.3%	−0.91 [−1.95, 0.12]
Petrinović-Doresić, et al 1999* (c)	2,081.95	1,197.2	14	88.43	63.33	10	3.3%	2.09 [1.05, 3.13]
Sijssens et al, 2007 (a)	106	2,454.75	25	6	1.5	6	3.5%	0.04 [−0.85, 0.93]
Sijssens et al, 2007* (b)	3,509.25	3,454.75	25	4.5	1.5	6	3.4%	1.09 [0.15, 2.02]
van Kooij et al, 2006*	2,783.5	3,445.5	31	87	52	11	3.6%	0.89 [0.17, 1.60]
Wakefield et al, 1995	135,000	2,000	10	0.5	0.5	7	0.0%	82.71 [50.98, 114.44]
**Subtotal (95% CI)**			**119**			**60**	**20.7%**	**0.67 [−0.45, 1.79]**
Heterogeneity: Tau^2 = 1.74; Chi^2 = 45.66, df = 6 (P < 0.00001); I^2 = 87%								
Test for overall effect: Z = 1.17 (P = 0.24)								
**1.1.2 Infectious**								
Ang et al, 2012* (a)	470.4	276.8	10	3.4	0.6	23	3.3%	3.05 [1.97, 4.14]
Curnow et al, 2005* (c)	8,082	5,796.5	5	324	279.5	12	2.9%	2.45 [1.05, 3.86]
Murray et al, 1990 (a)	543	1,775	16	0.5	0.5	13	3.6%	0.40 [−0.34, 1.14]
Sauer et al, 2018*	12,662.5	5,177.5	46	55.75	24.25	60	3.7%	3.67 [3.04, 4.31]
Zenkel et al, 2010 (e)	193	144	14	52	34	14	3.5%	1.31 [0.48, 2.14]
**Subtotal (95% CI)**			**91**			**122**	**17.1%**	**2.17 [0.78, 3.55]**
Heterogeneity: Tau^2 = 2.26; Chi^2 = 50.54, df = 4 (P < 0.00001); I^2 = 92%								
Test for overall effect: Z = 3.06 (P = 0.002)								
**1.1.3 Inflammatory**								
Ang et al, 2012* (b)	21.8	6.1	15	3.4	0.6	23	3.1%	4.70 [3.41, 5.99]
Bae and Lee, 2012	212.84	150.3	7	16.59	31.24	23	3.3%	2.55 [1.46, 3.64]
Chen et al, 2014 (a)	22.96	6.68	10	9.31	6.1	10	3.2%	2.04 [0.92, 3.17]
Chen et al, 2014 (b)	28.34	17.8	9	9.31	6.1	10	3.3%	1.40 [0.37, 2.43]
Curnow et al, 2005* (a)	230,054	199,997.5	23	324	279.5	12	3.6%	1.37 [0.60, 2.15]
Curnow et al, 2005* (d)	10,129	3,155	4	324	279.5	12	1.7%	6.26 [3.52, 9.00]
Murugeswari et al, 2008*	400.95	300	10	0.5	0.5	10	3.3%	1.81 [0.73, 2.88]
Murugeswari et al, 2014	74.48	86.05	5	0.5	0.5	5	3.0%	1.10 [−0.29, 2.48]
Nagata et al, 2012	736	276.5	19	13.8	4.2	5	3.0%	2.79 [1.48, 4.10]
Petrinović-Doresić, et al 1999* (a)	1,023.9	634.55	10	88.43	63.33	10	3.3%	1.99 [0.87, 3.10]
Petrinović-Doresić, et al 1999* (b)	157.1	134.65	13	88.43	63.33	10	3.5%	0.60 [−0.24, 1.45]
Valentincic et al, 2011*	6.6	3.75	36	4.33	1.38	10	3.6%	0.66 [−0.06, 1.37]
**Subtotal (95% CI)**			**161**			**140**	**38.0%**	**2.03 [1.35, 2.72]**
Heterogeneity: Tau^2 = 1.11; Chi^2 = 53.31, df = 11 (P < 0.00001); I^2 = 79%								
Test for overall effect: Z = 5.79 (P < 0.00001)								
**1.1.4 Pseudo-uveitis**								
Aketa et al, 2017 (a)	1,696	805	15	6.4	0.9	30	3.4%	3.61 [2.62, 4.61]
Banerjee et al, 2007* (b)	5,000	7,926.25	15	6	6.25	8	3.5%	0.74 [−0.15, 1.63]
Nagarkatti-Gude et al, 2012	107.89	66.25	33	38.53	28.46	9	3.6%	1.12 [0.34, 1.90]
**Subtotal (95% CI)**			**63**			**47**	**10.5%**	**1.81 [0.17, 3.45]**
Heterogeneity: Tau^2 = 1.90; Chi^2 = 20.63, df = 2 (P < 0.0001); I^2 = 90%								
Test for overall effect: Z = 2.16 (P = 0.03)								
**1.1.5 Ophthalmologic entities**								
Curnow et al, 2005* (b)	706.75	631.75	5	324	279.5	12	3.3%	0.90 [−0.20, 2.00]
Kuiper et al, 2011*	83.9	73.05	16	7.1	3.5	11	3.5%	1.32 [0.46, 2.17]
Murray et al, 1990 (b)	19.23	50.08	8	0.5	0.5	13	3.5%	0.59 [−0.31, 1.49]
Perez et al, 2004*	61.64	43.25	23	10.6	7	8	3.5%	1.31 [0.44, 2.19]
**Subtotal (95% CI)**			**52**			**44**	**13.8%**	**1.05 [0.59, 1.51]**
Heterogeneity: Tau^2 = 0.00; Chi^2 = 1.78, df = 3 (P = 0.62); I^2 = 0%								
Test for overall effect: Z = 4.48 (P < 0.00001)								
**Total (95% CI)**			**486**			**413**	**100.0%**	**1.62 [1.15, 2.09]**
Heterogeneity: Tau^2 = 1.45; Chi^2 = 212.23, df = 30 (P < 0.00001); I^2 = 86%								
Test for overall effect: Z = 6.74 (P < 0.00001)								
Test for subgroup differences: Chi^2 = 8.43, df = 4 (P = 0.08), I^2 = 52.6%								

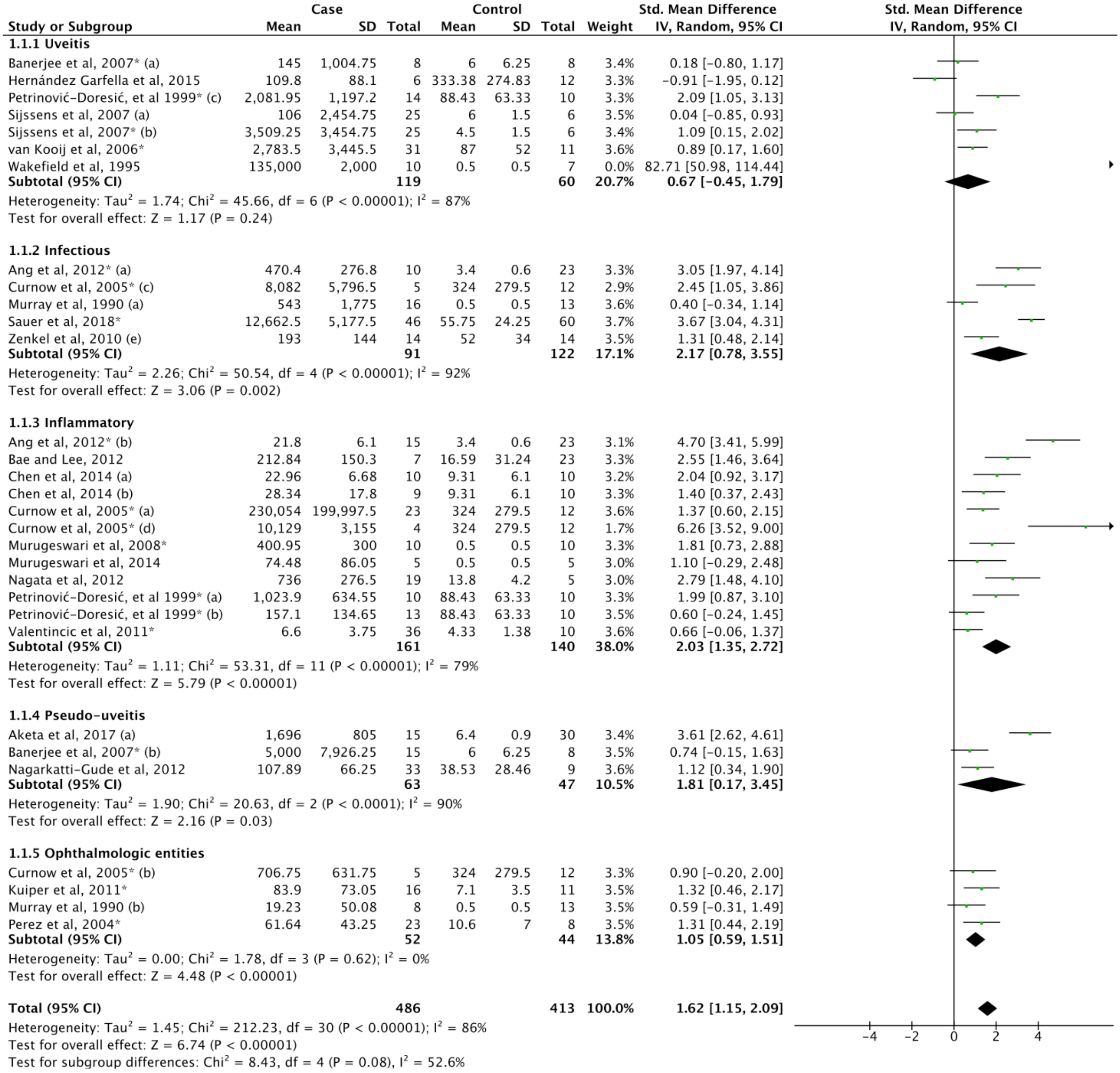

Supplemental Figure S11. Forest plot for pooled SMD and 95% CI for IL-6 levels between intraocular inflammation (uveitis) and control. Note: 1.1.1, unclassified uveitis subtype (refer to the Supplemental Table S4 (4b1).

A

Study or Subgroup	CRVO Mean	CRVO SD	CRVO Total	BRVO Mean	BRVO SD	BRVO Total	Weight	Std. Mean Difference IV, Random, 95% CI
Feng et al, 2013	0.96	0.24	18	0.96	0.2	11	13.9%	0.00 [−0.75, 0.75]
Funk et al, 2009*	83.2	66.2	8	20.5	21.9	5	9.2%	1.07 [−0.15, 2.29]
Koss et al, 2012 (a)	64.7	115.8	35	23.2	48.8	43	17.4%	0.48 [0.03, 0.93]
Koss et al, 2012 (b)	59.9	97.5	16	23.2	48.8	43	15.9%	0.56 [−0.03, 1.14]
Rezar-Dreindl et al, 2016*	14.25	12.45	15	13.4	13.4	25	15.2%	0.06 [−0.58, 0.70]
Shchuko et al, 2015	285.73	232.51	18	65.82	97.46	25	14.9%	1.29 [0.62, 1.96]
Yoshimura et al, 2009*	3,275	2,768.25	13	121.8	74	30	13.4%	2.06 [1.27, 2.86]
**Total (95% CI)**			**123**			**182**	**100.0%**	**0.75 [0.25, 1.25]**

Heterogeneity: $Tau^2 = 0.32$; $Chi^2 = 22.84$, df = 6 (P = 0.0009); $I^2 = 74\%$
Test for overall effect: Z = 2.92 (P = 0.003)

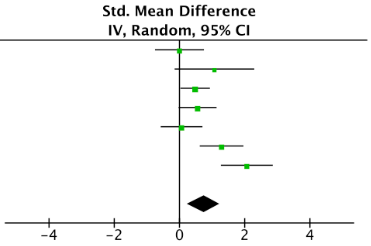

B

Study or Subgroup	Case Mean	Case SD	Case Total	Control Mean	Control SD	Control Total	Weight	Std. Mean Difference IV, Random, 95% CI
**1.1.1 CRVO+MO**								
Noma et al, 2009*	63.79	41.17	16	1.12	0.2	16	5.5%	2.10 [1.21, 2.98]
Noma et al, 2010*	65.79	41.17	16	2.32	0.76	8	5.4%	1.80 [0.79, 2.82]
Noma et al, 2013* (a)	57.35	20.65	15	0.92	0.36	17	5.1%	3.90 [2.66, 5.13]
Noma et al, 2013* (b)	18.45	8.7	6	0.92	0.36	17	4.6%	3.97 [2.40, 5.54]
Noma et al, 2015*	15.8	5.4	38	1.59	0.8	15	5.6%	3.03 [2.18, 3.88]
Wen et al, 2014 (a)	1.86	0.12	20	1.17	0.17	15	4.9%	4.70 [3.36, 6.05]
**Subtotal (95% CI)**			**111**			**88**	**31.1%**	**3.15 [2.28, 4.03]**
Heterogeneity: $Tau^2 = 0.86$; $Chi^2 = 18.90$, df = 5 (P = 0.002); $I^2 = 74\%$								
Test for overall effect: Z = 7.06 (P < 0.00001)								
**1.1.2 CRVO**								
Chen et al, 1999 (a)	15.6	0.9	5	26.4	21.8	6	5.1%	−0.61 [−1.84, 0.62]
Chen et al, 1999 (b)	1,532	221.1	10	26.4	21.8	6	2.4%	8.01 [4.66, 11.36]
Chen et al, 1999 (c)	234.3	154.6	9	26.4	21.8	6	5.1%	1.60 [0.37, 2.83]
Ehlken et al, 2015	43	38	13	4	3	13	5.6%	1.40 [0.53, 2.27]
Feng et al, 2013 (b)	0.96	0.24	18	0.41	0.08	10	5.3%	2.67 [1.59, 3.75]
Funk et al, 2009* (a)	83.2	66.2	8	2.1	2.7	13	5.3%	1.93 [0.84, 3.03]
Koss et al, 2012 (a)	64.7	115.8	35	6.2	3.4	14	5.8%	0.58 [−0.05, 1.22]
Koss et al, 2012 (b)	59.9	97.5	16	6.2	3.4	14	5.7%	0.73 [−0.01, 1.48]
Koss et al, 2013	64.7	115.8	35	6.2	3.4	28	5.9%	0.67 [0.16, 1.18]
Rezar-Dreindl et al, 2016* (a)	14.25	12.45	15	72.95	72.95	15	5.7%	−1.09 [−1.87, −0.32]
Shchuko et al, 2015 (a)	285.73	232.51	18	52.19	34.55	20	5.7%	1.41 [0.69, 2.13]
Suzuki et al, 2011	3,427.4	5,429.39	23	188.24	167.78	10	5.7%	0.69 [−0.07, 1.45]
Yoshimura et al, 2009* (b)	3,275	2,768.25	13	65.25	44.2	83	5.7%	3.22 [2.47, 3.96]
**Subtotal (95% CI)**			**218**			**238**	**68.9%**	**1.30 [0.60, 2.00]**
Heterogeneity: $Tau^2 = 1.37$; $Chi^2 = 104.56$, df = 12 (P < 0.00001); $I^2 = 89\%$								
Test for overall effect: Z = 3.64 (P = 0.0003)								
**Total (95% CI)**			**329**			**326**	**100.0%**	**1.91 [1.25, 2.57]**

Heterogeneity: $Tau^2 = 1.84$; $Chi^2 = 181.02$, df = 18 (P < 0.00001); $I^2 = 90\%$
Test for overall effect: Z = 5.68 (P < 0.00001)
Test for subgroup differences: $Chi^2 = 10.52$, df = 1 (P = 0.001), $I^2 = 90.5\%$

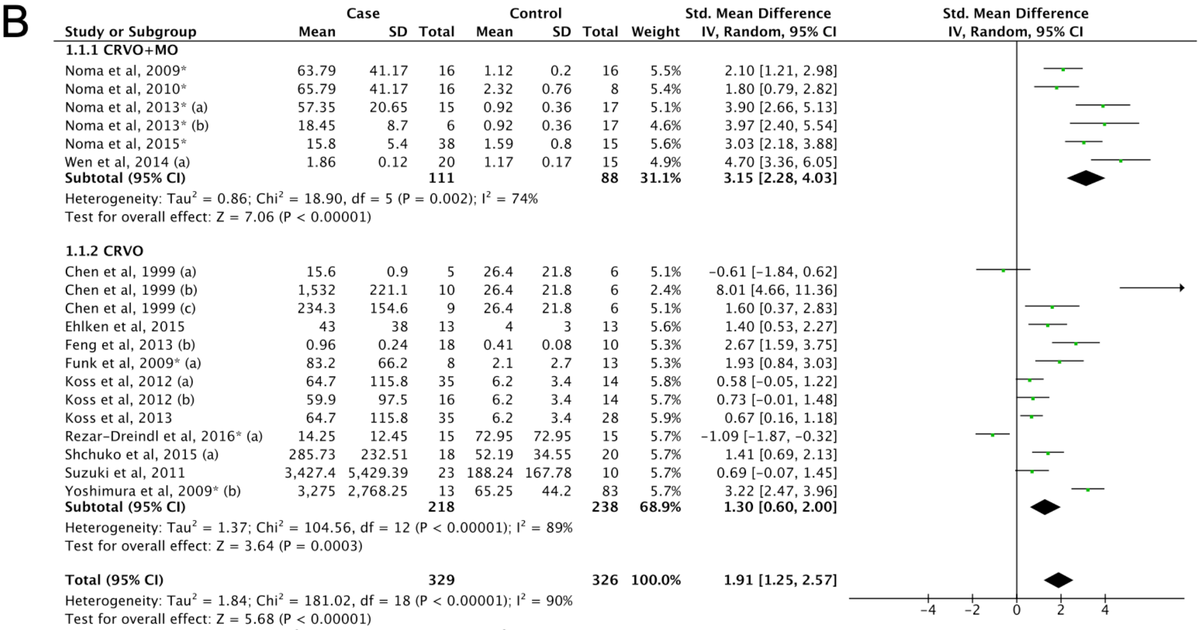

C

Study or Subgroup	Case Mean	Case SD	Case Total	Control Mean	Control SD	Control Total	Weight	Std. Mean Difference IV, Random, 95% CI
**1.1.1 BRVO+MO**								
Lee et al, 2012 (a)	17.7	26.9	12	28.8	39.2	16	5.3%	−0.31 [−1.07, 0.44]
Noma et al, 2005*	9.7	5.85	19	2.4	0.13	7	5.0%	1.40 [0.44, 2.35]
Noma et al, 2006*	69.29	47.76	25	1.64	0.85	14	5.3%	1.72 [0.95, 2.49]
Noma et al, 2013* (c)	13.98	5.87	39	0.92	0.36	17	5.3%	2.61 [1.85, 3.37]
Noma et al, 2014* (a)	11.98	4.27	28	0.96	0.14	17	5.1%	3.20 [2.28, 4.12]
Noma et al, 2014* (b)	8.62	1.88	41	3.53	1.95	9	5.1%	2.65 [1.75, 3.55]
Shimura et al, 2008	108.6	79.5	60	11.8	2.85	12	5.4%	1.31 [0.65, 1.97]
Sohn et al, 2014* (a)	23.97	11.65	12	4.23	0.56	6	4.6%	1.95 [0.73, 3.16]
Sohn et al, 2014* (b)	15.8	0.962	12	4.23	0.56	6	1.1%	12.86 [8.01, 17.71]
**Subtotal (95% CI)**			**248**			**104**	**42.3%**	**2.11 [1.20, 3.03]**
Heterogeneity: $Tau^2 = 1.58$; $Chi^2 = 69.57$, df = 8 (P < 0.00001); $I^2 = 89\%$								
Test for overall effect: Z = 4.54 (P < 0.00001)								
**1.1.2 BRVO**								
Feng et al, 2013 (c)	0.96	0.2	11	0.41	0.08	10	4.3%	3.40 [1.98, 4.83]
Funk et al, 2009* (b)	20.5	21.9	5	2.1	2.7	13	4.7%	1.57 [0.38, 2.75]
Kaneda et al, 2011	18.6	2.6	38	10.3	1.4	28	5.2%	3.77 [2.95, 4.59]
Koss et al, 2012 (c)	23.2	48.8	43	6.2	3.4	14	5.5%	0.39 [−0.21, 1.00]
Lim, 2011	7.5	5.6	46	2	1.3	10	5.4%	1.06 [0.34, 1.77]
Noma et al, 2016*	4.33	1.92	38	1	0.47	10	5.3%	1.89 [1.09, 2.69]
Park and Ahn, 2009	38	31	18	2.3	1.6	10	5.2%	1.38 [0.52, 2.25]
Pfister et al, 2013	23.3	48.8	43	6.2	3.4	28	5.6%	0.44 [−0.04, 0.93]
Rezar-Dreindl et al, 2016* (b)	13.4	13.4	25	72.95	72.95	15	5.4%	−1.28 [−1.99, −0.58]
Shchuko et al, 2015 (b)	65.82	97.46	26	52.19	34.55	20	5.5%	0.17 [−0.41, 0.76]
Yoshimura et al, 2009* (c)	121.8	74	30	65.25	44.2	83	5.7%	1.05 [0.61, 1.49]
**Subtotal (95% CI)**			**323**			**241**	**57.7%**	**1.19 [0.48, 1.90]**
Heterogeneity: $Tau^2 = 1.28$; $Chi^2 = 116.09$, df = 10 (P < 0.00001); $I^2 = 91\%$								
Test for overall effect: Z = 3.28 (P = 0.001)								
**Total (95% CI)**			**571**			**345**	**100.0%**	**1.58 [1.00, 2.15]**

Heterogeneity: $Tau^2 = 1.46$; $Chi^2 = 208.47$, df = 19 (P < 0.00001); $I^2 = 91\%$
Test for overall effect: Z = 5.40 (P < 0.00001)
Test for subgroup differences: $Chi^2 = 2.45$, df = 1 (P = 0.12), $I^2 = 59.2\%$

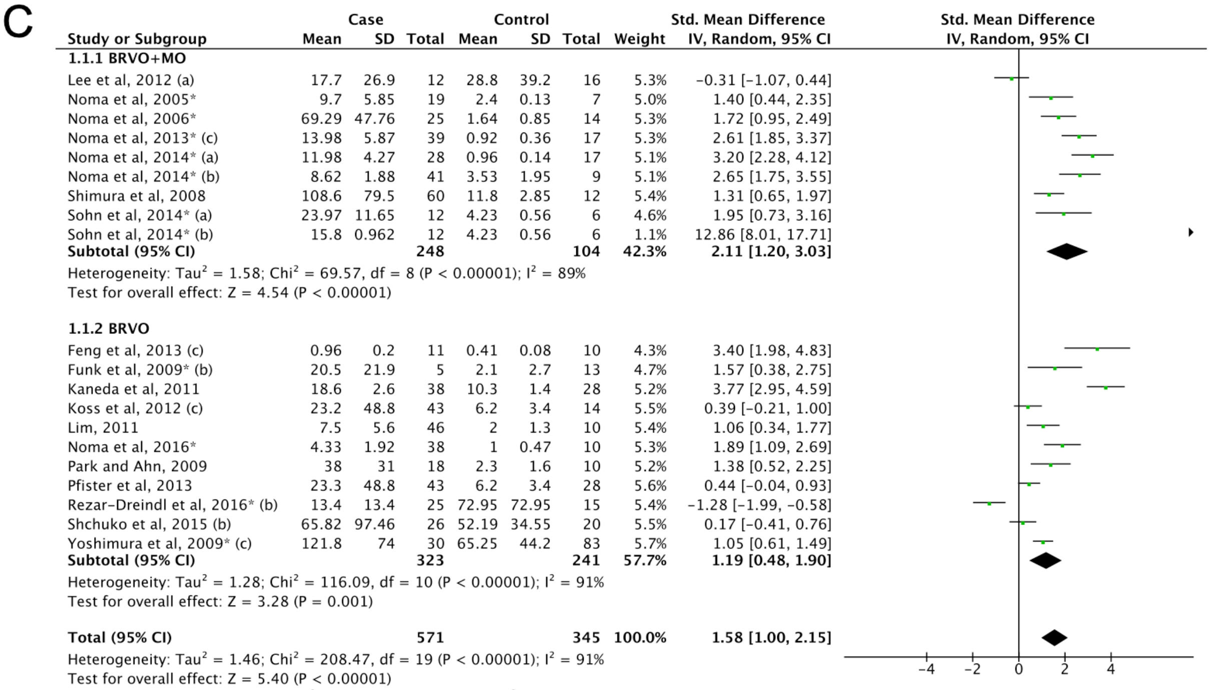

Supplemental Figure S12. (A) Forest plot for pooled SMD and 95% CI for IL-6 levels between CRVO and BRVO; **(B)** Forest plot for pooled SMD and 95% CI for IL-6 levels between CRVO and CRVO+MO; **(C)** Forest plot for pooled SMD and 95% CI for IL-6 levels between BRVO and BRVO+MO.

A

Study or Subgroup	Case Mean	Case SD	Case Total	Control Mean	Control SD	Control Total	Weight	Std. Mean Difference IV, Random, 95% CI
Fauser et al, 2015	53	168	17	5	12	17	7.3%	0.39 [−0.29, 1.07]
Jonas et al, 2012 (a)	154	326.25	18	6.42	17.28	20	7.5%	0.64 [−0.01, 1.30]
Kramer et al, 2012 (b)	886	1,201	16	351	516	18	7.3%	0.58 [−0.11, 1.27]
Miao et al, 2012	3.6	1.8	17	4.47	2.07	14	7.1%	−0.44 [−1.16, 0.28]
Rezar−Dreindl et al, 2016 (a)	3.5	8.9	20	1.4	1	15	7.4%	0.30 [−0.37, 0.98]
Rezar−Dreindl et al, 2016 (b)	1.8	5.4	20	1.4	1	15	7.4%	0.09 [−0.58, 0.76]
Roh et al, 2009 (c)	14.5	13.1	19	20.9	30.5	19	7.6%	−0.27 [−0.91, 0.37]
Roh et al, 2009 (d)	46.9	59.5	14	20.9	30.5	19	7.2%	0.56 [−0.14, 1.27]
Roh et al, 2009 (e)	46.4	70.5	14	20.9	30.5	19	7.2%	0.49 [−0.22, 1.19]
Roh et al, 2010	73.5	172.1	10	9.5	14.6	9	6.0%	0.49 [−0.43, 1.40]
Sakurada et al, 2015	10.9	13.8	18	9.9	12.6	20	7.6%	0.07 [−0.56, 0.71]
Sato et al, 2018	6.51	5.24	21	78.2	100	17	7.3%	−1.05 [−1.74, −0.36]
Spindler et al, 2018 (b)	17.1	21.3	19	4.7	7.1	20	7.5%	0.77 [0.12, 1.43]
Terao et al, 2018*	7.43	2.17	18	3.29	0.36	11	5.6%	2.32 [1.33, 3.31]
**Total (95% CI)**			**241**			**233**	**100.0%**	**0.32 [−0.03, 0.66]**

Heterogeneity: $Tau^2 = 0.30$; $Chi^2 = 43.54$, df = 13 ($P < 0.0001$); $I^2 = 70\%$
Test for overall effect: Z = 1.81 (P = 0.07)

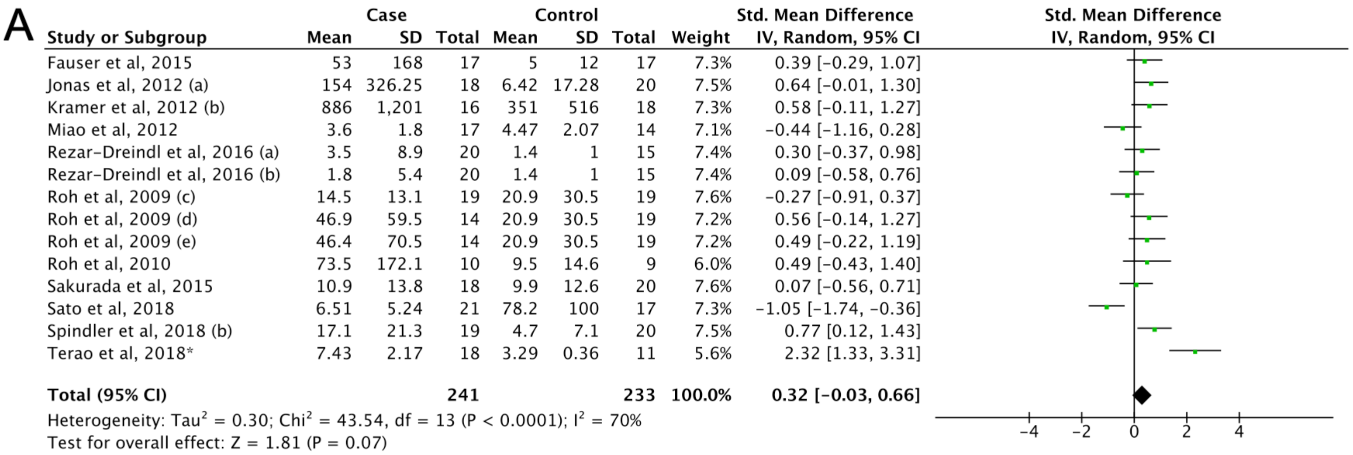

B

Study or Subgroup	Dry AMD Mean	Dry AMD SD	Dry AMD Total	Wet AMD Mean	Wet AMD SD	Wet AMD Total	Weight	Std. Mean Difference IV, Random, 95% CI
Kramer et al, 2012	637	1,010	16	886	1,201	16	48.0%	−0.22 [−0.91, 0.48]
Spindler et al, 2018	3.4	1.8	25	17.1	21.3	19	52.0%	−0.96 [−1.59, −0.33]
**Total (95% CI)**			**41**			**35**	**100.0%**	**−0.60 [−1.33, 0.12]**

Heterogeneity: $Tau^2 = 0.16$; $Chi^2 = 2.39$, df = 1 ($P = 0.12$); $I^2 = 58\%$
Test for overall effect: Z = 1.63 (P = 0.10)

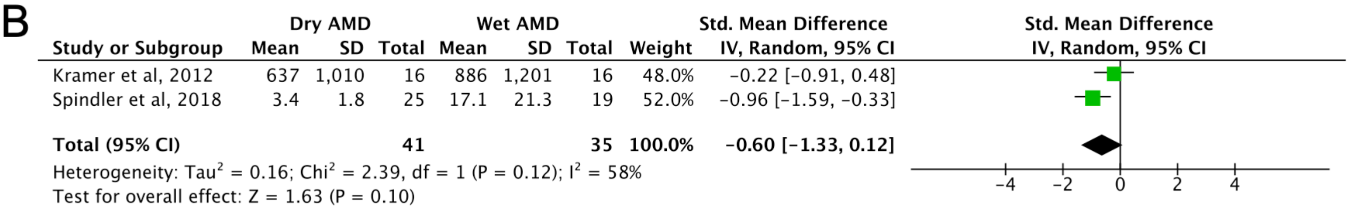

Supplemental Figure S13. (A) Forest plot for pooled SMD and 95% CI for IL-6 levels between AMD and control; **(B)** Forest plot for pooled SMD and 95% CI for IL-6 levels between dry and wet AMD.
